# Programmable hydrodynamics of active particles

**DOI:** 10.1038/s41467-026-77281-x

**Published:** 2026-09-02

**Authors:** Lisa Rohde, Gordei Anchutkin, Viktor Holubec, Frank Cichos

**Affiliations:** 1 Molecular Nanophotonics Group, Peter Debye Institute for Soft Matter Physics, Leipzig University, Leipzig, Germany; 2 Department of Macromolecular Physics, Faculty of Mathematics and Physics, Charles University, Prague, Czech Republic

**Keywords:** Statistical physics, Fluid dynamics, Fluids

## Abstract

Self-propelled microparticles create flow fields that determine how they interact with surfaces, external flows, and each other. These flow fields fall into distinct classes–pushers, pullers, and neutral swimmers–each exhibiting fundamentally different collective behaviors. In all existing synthetic systems, this hydrodynamic character is permanently set during fabrication. Here we demonstrate that the hydrodynamic identity of microswimmers can be programmed and switched on demand. Using patterned laser heating of surface-bound nanoparticles, we create tailored temperature gradients that drive controllable boundary flows. Real-time illumination control transforms the swimmer’s flow field from pusher to puller during motion. Flow measurements confirm quantitative agreement with theory and track changes in power consumption and efficiency across modes. This control over hydrodynamic modes provides an experimental platform to explore active matter behavior under external physical constraints, including adaptive swimmer responses to crowding or confinement and the emergence of collective states from tunable pusher-puller mixtures.

## Abstract (web summary)

The hydrodynamic class of a microswimmer- pusher, puller, or neutral is fixed during fabrication, limiting exploration of adaptive behaviors. The authors show that the hydrodynamic flow field of a synthetic microswimmer can be dynamically programmed and switched in real time using structured laser heating, enabling reversible transitions between different swimming modes.

## Introduction

Micron-sized self-propelled particles, from swimming bacteria and motile algae to synthetic colloidal swimmers, generate long-range hydrodynamic flow fields^1–3^. The morphology of these flow fields governs how efficiently swimmers propel and how they interact with surfaces, external flows, and each other to yield collective behaviors. Swimmers are classified by their far-field flow structure as pushers (bacteria like E. coli that push fluid from the rear^4–6^), pullers (algae like *Chlamydomonas* that pull fluid from the front^7^), or neutral squirmers^8–11^. These classes are distinguished by their leading hydrodynamic multipole: a slowly-decaying force dipole ( ∝ *r*^−2^) for pushers and pullers, and a faster-decaying source dipole ( ∝ *r*^−3^) for neutral swimmers, which sets the spatial reach of their hydrodynamic interactions.

Although the neutral squirmer is rarely realized in nature owing to asymmetries, time-dependent stroke cycles, and propulsion constraints, it serves as the canonical idealization for theories of efficient propulsion. A fundamental prediction of hydrodynamic theory is that a spherical neutral squirmer achieves optimal swimming efficiency by eliminating slowly-decaying force dipole contributions to viscous dissipation that do not contribute to propulsion^12–15^. This result has become a cornerstone of active matter theory and is widely invoked to explain performance optimization in biological and synthetic swimmers, although variations with shape have been shown^12^. It has, however, been derived under the assumption that surface boundary conditions can be prescribed at will.

Real swimming systems, whether biological or synthetic, operate under physical constraints that limit achievable surface flows. Bacteria cannot arbitrarily program their flagellar arrangements^16,17^ and have evolved those functional systems under constraints, chemically-powered Janus particles face asymmetric catalyst placement constraints^18–22^, and thermophoretic swimmers require specific heating distributions^23–25^. Although such constraints are theoretically expected to alter performance optima, no experimental system has tested this prediction. In existing synthetic swimmers the hydrodynamic mode is permanently fixed during fabrication^26–28^, and even recent advances in switching propulsion direction or activity states^25,29,30^ have not enabled dynamic reconfiguration of the flow field signature itself between pusher, puller, and neutral modes. Resolving this gap matters beyond efficiency optimization. Understanding how physical constraints shape morphological adaptation is essential for interpreting biological swimming strategies^7,9,11,31^ and for developing design principles for synthetic active matter^32–35^. Testing the prediction therefore requires precise, real-time control over surface boundary conditions, which also allows isolation of the swimming mode itself, a more robust basis for comparison than approaches that vary particle type, shape, or surface chemistry.

Here, we address this challenge by introducing a synthetic microswimmer platform whose hydrodynamic flow field can be reconfigured in real time. Using spatially structured laser heating of gold nanoparticle-decorated microparticles, we program the surface temperature distribution and thereby the thermo-osmotic boundary flow, allowing us to switch a single particle between pusher, puller, and neutral modes while it continues to swim. We map the resulting flow fields across the full squirmer parameter range and, because the absorbed optical power that drives propulsion is directly accessible in our system, simultaneously quantify the energetic cost of each mode. This combination of programmable boundary conditions and direct access to the input power makes it possible to experimentally chart how swimming efficiency varies with hydrodynamic signature under the physical constraints imposed by a realistic propulsion mechanism. In the following, we describe the experimental realization of programmable surface heating, demonstrate the on-demand reconfiguration of the flow field, and analyze how the interplay between hydrodynamic mode and mechanism-specific constraints shapes the performance of the swimmer.

## Results

### Precise control of surface temperature patterns

**Fig. 1:**
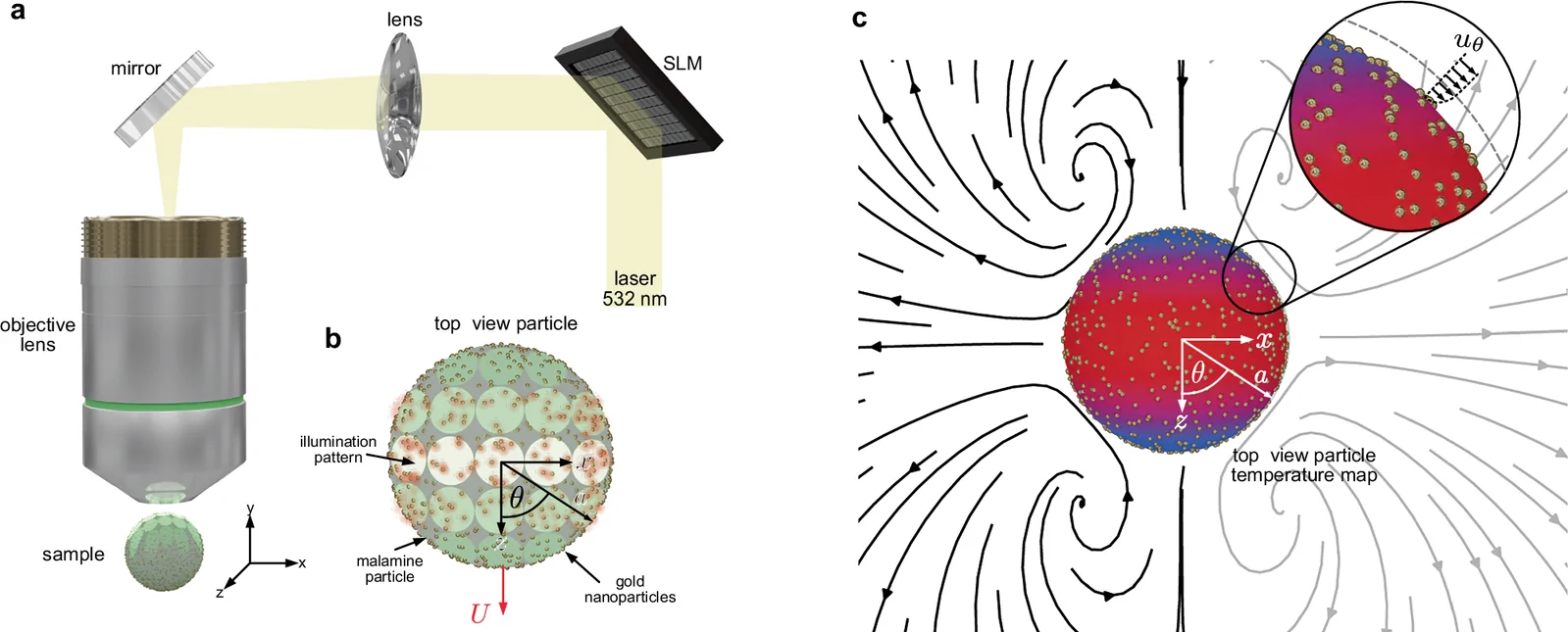
Experimental realization of hydrodynamic reconfiguration. **a** We control microswimmer flow fields by patterning laser intensity on the particle surface using a spatial light modulator (SLM). **b** Top view of the microswimmer consisting of a melamine resin sphere with radius *a* = 2.2 μm, decorated with 10 nm gold nanoparticles and illuminated with laser light of wavelength 532 nm. The schematic structured light pattern is generated by multiple laser spots of different intensities focused on the microparticle surface obeying axial symmetry with *θ* defined as the polar angle to the positive *z*-axis, which also provides the propulsion direction with particle speed *U*. **c** The illumination pattern results in stronger heating (red color, equator of the particle at *θ* = *π*/2) in the high intensity regions as compared to the poles (blue color). The corresponding interfacial temperature gradients generate a thermo-osmotic boundary flow *u*_*θ*_ (top right) indicated by the black arrows. This boundary flow creates a hydrodynamic flow field that is characteristic of the selected swimming mode obeying force-dipole characteristics, as shown by the streamline plot.

Reconfiguring the hydrodynamic flow morphology of a microswimmer requires precise manipulation of the hydrodynamic boundary conditions on the particle’s surface. We achieve this by using a special type of thermophoretic swimmer^24^ consisting of melamine resin microspheres decorated with 10 nm diameter gold nanoparticles. The interfacial flow fields of the microparticles are programmed through structured laser illumination using a spatial light modulator (SLM, Fig. 1a). When illuminated at 532 nm near their plasmon resonance, the nanoparticles act as local point-like heat sources^36^. The sparse coverage (approximately 10% surface coverage^25^) maintains a low thermal conductivity along the surface, enabling the creation of well-defined temperature patterns by selectively illuminating different surface regions. Figure 1b displays this principle with a particular illumination pattern on the particle’s surface that generates a heated region at its equator on the illumination side of the particle. The resulting temperature distribution is indicated in Fig. 1c, with temperature gradients leading to thermo-osmotic boundary flows *u*_*θ*_ as indicated by the black arrows. These boundary flows generate a bulk hydrodynamic flow field sketched by the black streamlines in Fig. 1c.

To induce a specific hydrodynamic flow morphology we can follow the squirmer framework^1,2^, parameterizing the tangential quasi-slip velocity *u*_*θ*_(*θ*) at the particle surface (*r* = *a*) as

1 $${u}_{\theta }(\theta )={B}_{1}\sin \theta+{B}_{2}\sin \theta \cos \theta \,,$$

where *B*_1_ drives propulsion and *B*_2_ sets the far-field flow character. The dimensionless squirmer parameter *β* = *B*_2_/∣*B*_1_∣ classifies swimmers as pushers (*β* < 0, extensile), pullers (*β* > 0, contractile), or neutral (*β* = 0, pure source dipole). Equation (1) corresponds to the truncated Legendre polynomial expansion of the interfacial flow field *u*_*θ*_(*θ*) ^23^ (see Supplementary Note S1).

These interfacial flows are realized as thermo-osmotic boundary flows that are themselves driven by interfacial temperature fields^37^. The temperature fields arise from a surface heat source density *q*(*θ*) determined by the laser intensity pattern heating the gold nanoparticles. Exploiting the axial symmetry, we expand *q*(*θ*) in Legendre polynomials and truncate at second order to capture the essential physics:

2 $$q(\theta )={q}_{0}+{q}_{1}\cos \theta+{q}_{2}\frac{1}{2}(3{\cos }^{2}\theta -1)\,.$$

Since the heat source density scales linearly with the incident intensity/power, the expansion coefficients directly determine the illumination pattern geometry. With $\cos \theta=z/a$ relating the polar angle to the axial coordinate *z*, these coefficients correspond to constant (*q*_0_), linear (*q*_1_), and quadratic (*q*_2_) intensity profiles along the propulsion axis *z*. These heat source densities create temperature gradients that drive a thermo-osmotic slip velocity *u*_*s*_(*θ*) = (*χ*/*T*_0_)(1/*a*)∂*T*(*θ*)/∂*θ*, where *χ* is the thermo-osmotic mobility characterizing the liquid-solid interfacial interaction^38,39^. Solving the heat diffusion equation and matching to the desired squirmer modes (Eq. (1)) establishes a direct correspondence: *q*_1_ ∝ *B*_1_ and *q*_2_ ∝ *B*_2_, enabling programming of any target squirmer parameter *β* by adjusting the relative amplitudes *q*_2_/*q*_1_ (Supplementary Note S1 for detailed derivation).

**Fig. 2:**
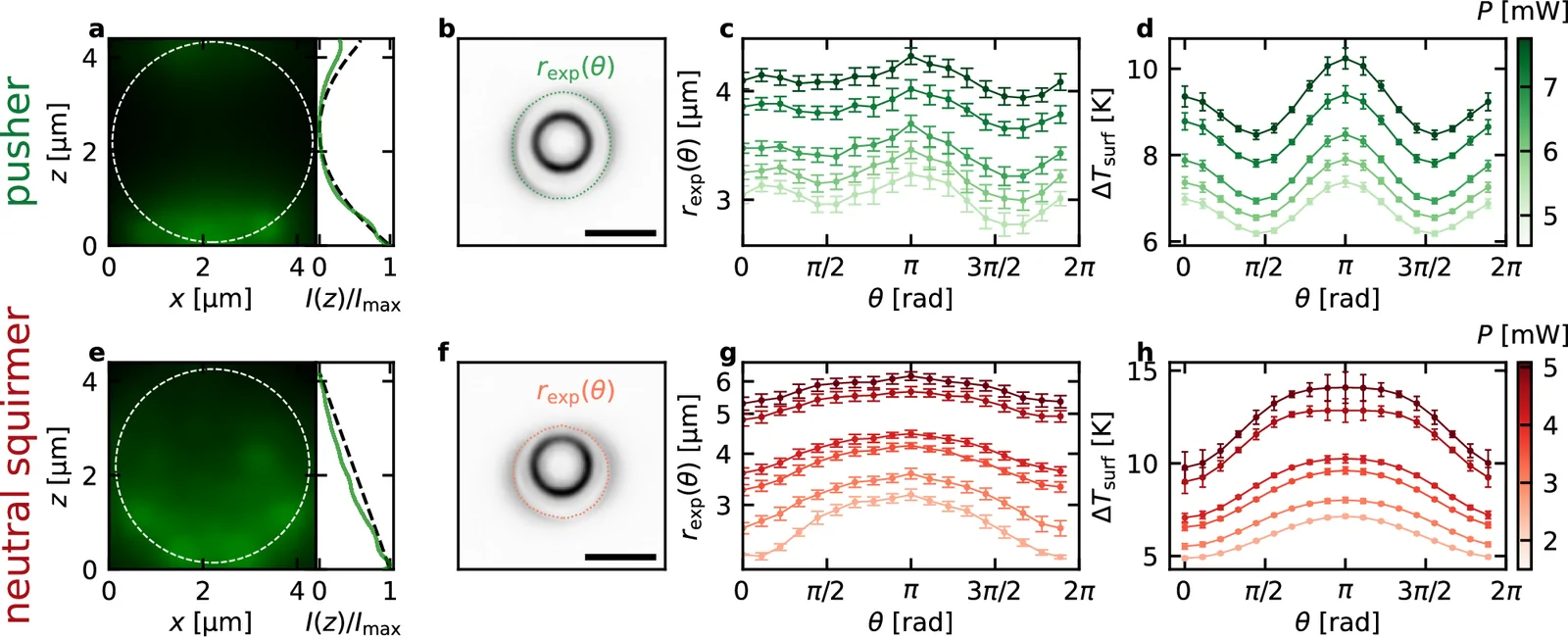
Controlled surface heating of microswimmers via structured illumination. For both pushers (*β* = − 1, **a–d**) and neutral squirmers (*β* = 0, **e–h**), we demonstrate precise control of surface temperature profiles. **a**, **e** Spatial light modulator (SLM) generates target illumination patterns (image: measured intensity using a fluorescence dye; green line: normalized experimental profile, dashed: theory from Eq. (2)). **b**, **f** Resulting asymmetric temperature fields visualized via liquid crystal phase boundary (*T*_*c*_ = 308 K); dashed lines show isotherms ${r}_{\exp }(\theta )$. Scale bar corresponds to 5 μm. **c**, **g** Power-dependent isotherm radii as a function of the polar angle *θ* confirm accurate spatial control. **d**, **h** Surface temperature difference Δ*T*_surf_(*θ*) reconstructed from isotherm asymmetry for different incident laser power *P*. Error bars represent the standard error of the mean calculated from measurements of 5 different particles for each swimming mode.

Figure 2a,e show this programmable control for two representative modes: a pusher (*β* = − 1) and neutral squirmer (*β* = 0). The intensity patterns, measured using fluorescence of a dye excited at *λ* = 532 nm, reveal the expected linear and quadratic profiles along the propulsion axis. Achieving such precise spatial control across the 4.4 μm diameter particle, only ~ 8 wavelengths, demonstrates the remarkable optical precision of our approach. In Fig. 2a,e the intensity line profiles (green curves) match theoretical predictions (black curves) within 10%, highlighting reproducible control over illumination patterns (Supplementary Figs. S2 and S3, Supplementary Note S2). A ray-optics analysis and generalized Lorenz–Mie theory (GLMT) simulations nevertheless show that the overall spatial structure of the imposed illumination profile remains well preserved across the particle and is only reduced in size. Importantly, these changes do not affect the symmetry of the resulting heating profile on the level of our experimental resolution (see Supplementary Note S2.4 for details).

We validate the resulting surface temperature distribution (Eq. (S17)) by immobilizing particles in a liquid crystal (5CB) and exploiting the nematic-isotropic phase transition at the critical temperature (Supplementary Note S3). The phase boundary, visible under dark field illumination due to the refractive index contrast between nematic and isotropic phase^40^, reveals the asymmetric temperature profile as shown in Fig. 2b, f for the pusher and neutral squirmer (inverted dark field intensity). The isotherm at the phase transition temperature *T*_PT_ = 308 K (colored dashed line) defines a radius ${r}_{\exp }(\theta )$ that maps the asymmetric heating. Figure 2c,g show ${r}_{\exp }(\theta )$ for different laser powers, confirming high-precision control (see also Supplementary Fig. S4). From ${r}_{\exp }(\theta )$, we directly extract the heat source coefficients *q*_0_, *q*_1_, and *q*_2_ from Eq. (2), enabling quantitative reconstruction of the surface temperature difference Δ*T*_surf_(*θ*) (Supplementary Note S3 and Supplementary Figs. S5, S6). Figure 2d, h shows Δ*T*_surf_(*θ*) for various laser input powers measured in the sample plane for the two swimming modes. While the neutral squirmer reaches a maximum temperature increment of 14 K at a laser power of *P* = 1.5 mW, the pusher’s maximum surface temperature increment remains below 10 K even at substantially higher laser powers, indicating differences in absorbed power between the swimming modes. The ability to precisely control surface temperature distributions is thus validated through both intensity measurements and direct liquid crystal thermography.

### Experimental demonstration of hydrodynamic mode reconfiguration

**Fig. 3:**
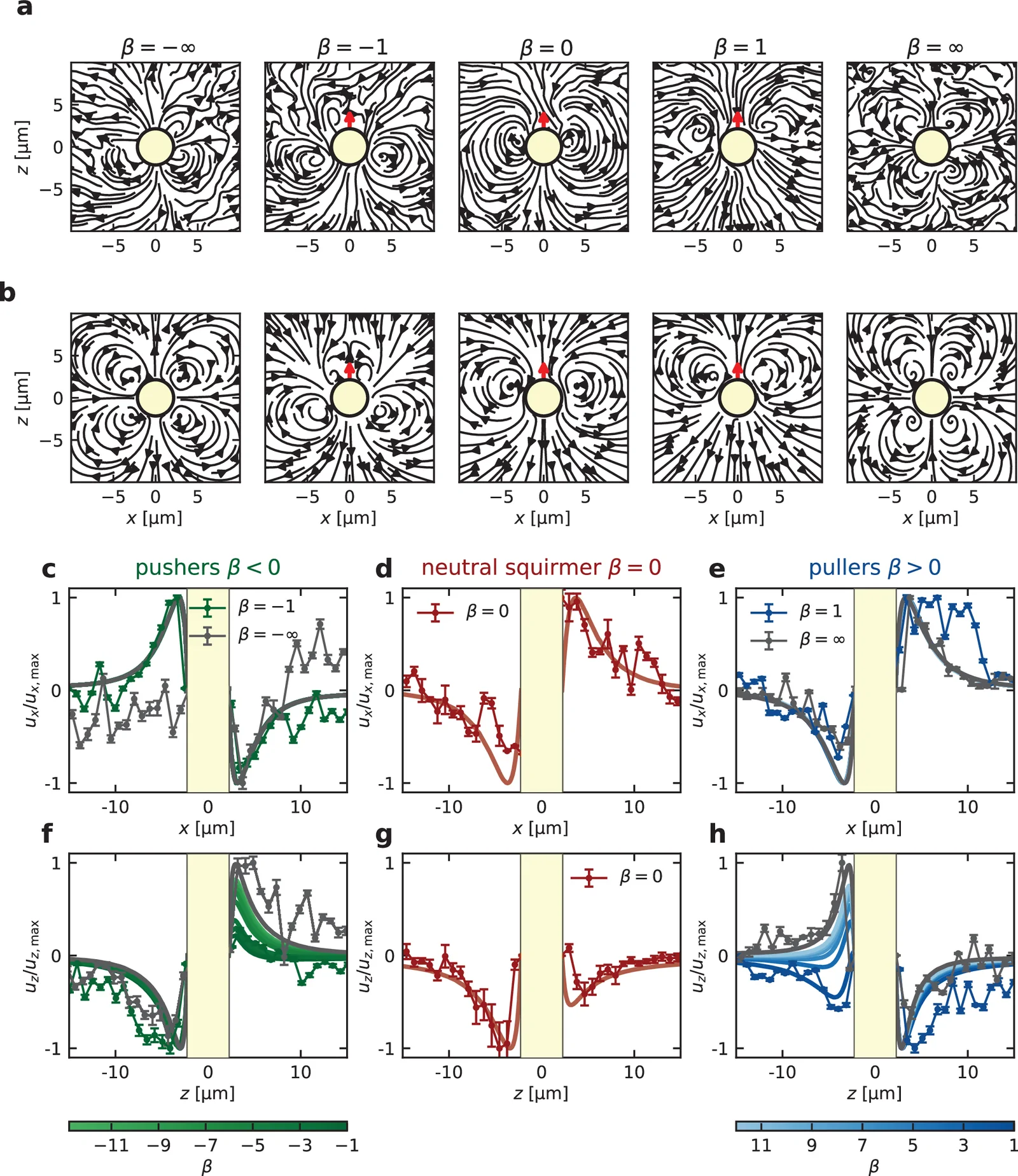
Reconfiguration of propulsion modes through programmable boundary hydrodynamics. **a** Experimental flow fields for various swimming modes characterized by the squirmer parameter *β* as determined from particle image velocimetry (PIV) of tracers around an immobilized microparticle of *a* = 2.2 μm when illuminated with different intensity patterns. The red arrows indicate the predefined propulsion direction. **b** Finite element simulation results for the hydrodynamic flow fields in the experimental configurations, including substrate boundaries. **c**–**e** Normalized experimental *x*-component of the velocity ${u}_{x}/{u}_{x,\max }$ (lines with markers) of pushers (*β* = − *∞*, *β* = − 1), neutral squirmers (*β* = 0) and pullers (*β* = *∞*, *β* = 1) along the *x*-direction as compared to simulation results (solid lines). The flow field structure is anti-symmetric along the *x-* direction as expected from theory. **f–h** Normalized experimental *z*-component ${u}_{z}/{u}_{z,\max }$ (lines with symbols) as compared to simulation data (solid lines) along the *z*-direction. Shaded regions denote the particle. Error bars represent the standard error of the mean calculated from flow field measurements of 5–11 different particles for each squirmer parameter.

The programmable illumination patterns translate directly into reconfigurable hydrodynamic signatures. Figure 3a shows flow fields for immobilized microswimmers spanning the complete range of propulsion modes, from negative shaker (*β* = − *∞*) through pusher (*β* = − 1), neutral squirmer (*β* = 0), and puller (*β* = 1) to positive shaker (*β* = *∞*) visualized using particle image velocimetry (PIV) with gold nanoparticle tracers with a diameter of 250 nm (see Supplementary Note S4 for details on PIV). The neutral squirmer exhibits a pure source dipole flow field. At *β* = ± *∞*, where the propulsive mode amplitude vanishes, only the force dipole signature remains.

Across all measured values of *β*, the experimental flow fields qualitatively match simulated results that explicitly account for the confinement of our experimental geometry (Fig. 3b; Supplementary Note S5). Three distinct effects distinguish the confined flow fields from those of an idealized squirmer in an unbounded fluid. First, holding the particle in place during the flow-field measurement requires an external force which, together with the particle-substrate interaction, exerts a net force on the surrounding fluid and thereby adds a slowly-decaying Stokeslet (force-monopole) contribution that is absent for a force-free swimmer. Second, the no-slip boundaries of the thin water film (*h* = 4.4 μm) hydrodynamically screen the flow, suppressing the far field on the scale of the film thickness. Third, the laser heating also raises the temperature of the confining walls, generating additional thermo-osmotic boundary flows there that further reshape the field. Together these effects alter the measured profiles relative to the unbounded case (see Supplementary Fig. S7). Most visibly, the pure source-dipole flow of the neutral squirmer (*β* = 0) acquires a finite *x*-component of the velocity at *z* = 0 under confinement, whereas it would vanish in free space (Supplementary Fig. S7). For the pusher shaker (*β* = − *∞*), small deviations between experiment and simulation arise mainly from the experimental difficulty of resolving the four vortices characteristic of a pure force-dipole flow field on a length scale of 1 μm. Nevertheless, the measured flow fields reproduce the characteristic signature of each mode, most reliably along the positive *x*-direction, confirming that varying only the illumination pattern reconfigures the swimmer’s complete hydrodynamic signature without any mechanical modification.

The velocity field components (Fig. 3c–h) reveal the underlying flow structure. The flow profiles shown in Fig. 3c–e are evaluated along the *x*-axis at *z* = 0, while Fig. 3f–h correspond to profiles along the *z*-axis at *x* = 0. Along the *x*-direction, the velocity component remains anti-symmetric about the swimmer center and exhibits only weak variations with increasing ∣*β*∣ within the same swimmer class (Fig. 3c–e). In contrast, the *z*-component shows a much stronger dependence on ∣*β*∣ (Fig. 3f–h). In particular, for increasing pusher strength, the magnitude of the *z*-component increases, reflecting enhanced fluid expulsion along the swimming axis and the formation of larger flow vortices. The experimental velocity profiles (symbols with error bars) are in good agreement with the simulated results (solid lines) and reproduce the characteristic spatial dependence of the different squirmer modes.

### Moving swimmers and their efficiencies

**Fig. 4:**
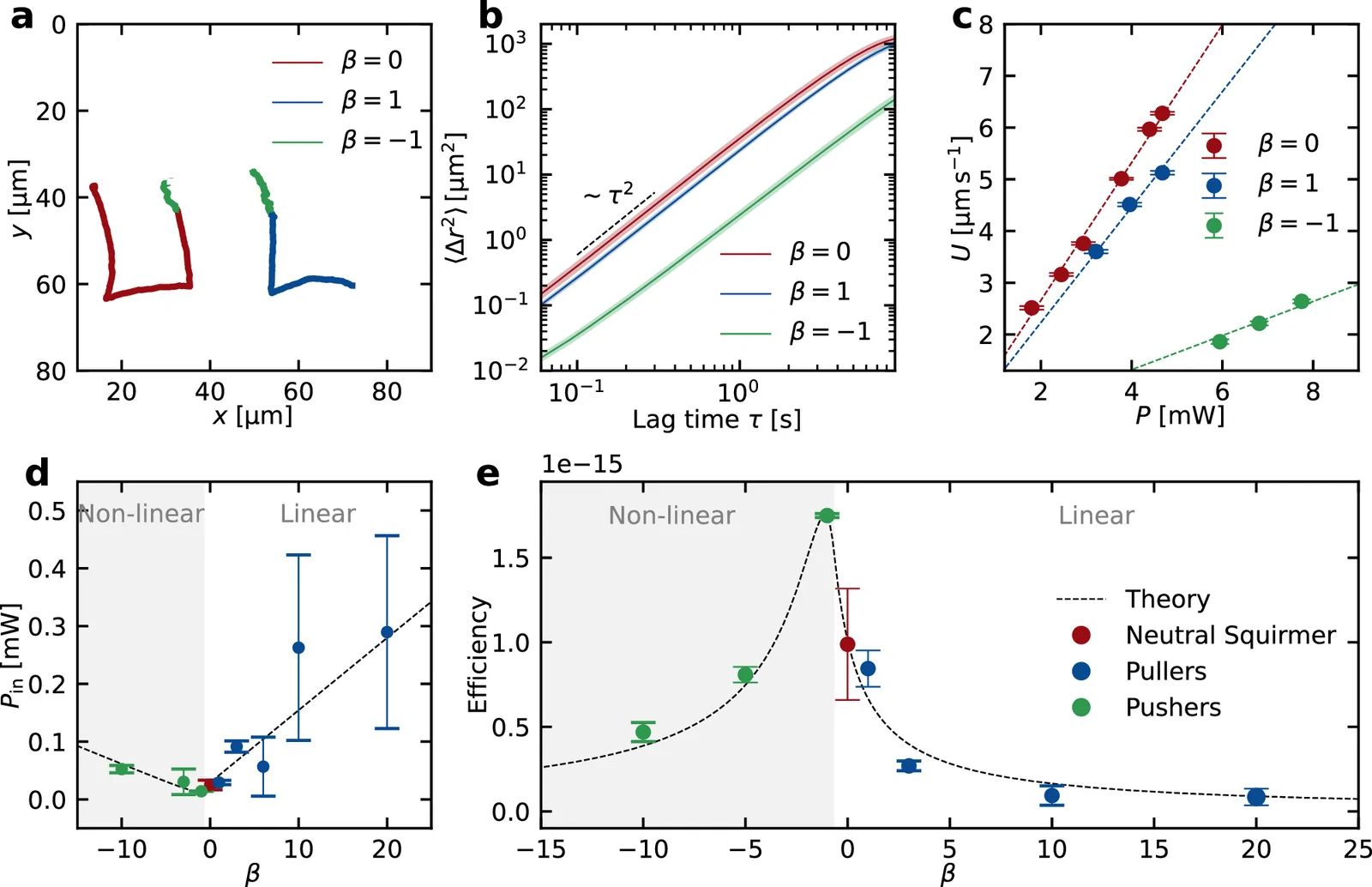
Reconfigurable swimming and efficiencies. **a** The trajectory of an active particle undergoing sequential transitions between distinct propulsion modes–from neutral squirmer (red) to pusher (green), and subsequently to puller (blue)–demonstrating the capability for fast hydrodynamic reconfiguration. **b** Mean squared displacement (MSD) as a function of lag time *τ* for a neutral squirmer (*β* = 0), puller (*β* = 1), and pusher (*β* = − 1) at the same incident laser power of *P* = 4.7 mW. The MSD scales as *τ*^2^ (dashed line), indicating quasi-ballistic motion of the active particle. **c** The velocity of the swimmers increases linearly with incident laser power *P*. The neutral squirmer reaches velocity values three times larger than the pusher. The dashed lines show fits of the form *U* = *mP* (*β* = 0: m=1.33 μm s^−1^mW^−1^, *β* = 1: m = 1.16 μm s^−1^mW^−1^, *β* = − 1: m = 0.33 μm s^−1^mW^−1^). **d** The input power *P*_in_ as a function of squirmer parameter *β* for constant speed *U* (*q*_1_ = − 0.4 μWm^−2^) indicating that the pusher with *β* ≈ − 1 exhibits the lowest input power. **e** Experimentally determined swimming efficiencies (symbols) for different swimming modes. The values align well with the theoretical model (dashed line), demonstrating that the pusher with *β* ≈ − 1 is the most efficient swimmer. The non-linear and linear regimes are indicated by the gray and white backgrounds, respectively, denoting the non-linear/linear relation between *q*_0_ and *q*_1_, *q*_2_ (see text). Error bars represent the standard error of the mean. Measurements were performed on 3 − 5 independent particles (see “Methods”).

The reconfigurable flow fields translate into programmable swimming behavior for freely moving particles. Suspending swimmers in a thin liquid film and dynamically varying the illumination pattern enables real-time switching between propulsion modes. We therefore track the position of the swimmers within each frame and reposition and reorient the pattern correspondingly^25^. Figure 4a shows a single swimmer transitioning from neutral squirmer (red) to pusher (green) to puller (blue) during continuous motion, demonstrating instantaneous reconfigurability without mechanical modification (Supplementary Movies 1 and 2).

The three modes exhibit at first glance strikingly different swimming characteristics. All show quasi-ballistic motion with a mean squared displacement scaling as *τ*^2^ (Fig. 4b), confirming active self-propulsion^18^. The rotational diffusion of the particle is irrelevant for the dynamics as the symmetry of the pattern defines the propulsion direction not the geometry. However, the propulsion velocities differ dramatically. At a constant incident laser power (*P* = 4.7 mW), the neutral squirmer reaches 6 μms^−1^, three times faster than the pusher. Velocities scale linearly with incident laser power for all modes (Fig. 4c), but the velocity-to-power ratios vary significantly, suggesting mode-dependent efficiencies as expected from the literature^12,14^.

These strong velocity differences raise the question which mode swims most efficiently? While the neutral squirmer moves fastest and is expected to be the most efficient for spherical particles^12,14^, speed alone may not determine efficiency. We calculate the swimming efficiency as the ratio of the power required to drag a swimmer at a given speed *U* (*P*_out_ = *F*_stokes_*U* = 6*πηaU*^2^) to the input power capturing all energetic contributions associated with propulsion and slip generation^41^:

3 $$\epsilon=\frac{{P}_{{{{\rm{out}}}}}}{{P}_{{{{\rm{in}}}}}}=\frac{6\pi \eta a{U}^{2}}{{\iint }_{{{{\rm{S}}}}}q(\theta )dS}=\frac{6\pi \eta a{U}^{2}}{4\pi {a}^{2}{q}_{0}}\,,$$

Here *q*(*θ*) is the heat source density over the sphere’s surface *S* responsible for generating the slip and thus activity (see Supplementary Note S6). Unlike virtually all other phoretic swimmer experiments, our experimental system allows direct determination of the input power *P*_in_, i.e. the power that generates the temperature field.

Extracting the heat source coefficients *q*_0_, *q*_1_, and *q*_2_ from the measured temperature profiles (Fig. 2c), we determine *P*_in_ for each mode at constant propulsion velocity *U* (i.e., fixed *q*_1_ = − 0.4 μWμm^−2^). Figure 4d reveals that the input power exhibits a pronounced minimum at *β* ≈ − 1 (pusher mode), not at the fastest-swimming neutral squirmer. This finding is independently confirmed by surface temperature measurements showing the pusher achieves the lowest Δ*T*_surf_ (Supplementary Fig. S5d).

The corresponding efficiencies (Fig. 4e), evaluated at a constant propulsion speed *U* (i.e., fixed *q*_1_ = − 0.4 μWμm^−2^), display a sharp maximum at *β* ≈ − 1. The efficiencies are as expected for many phoretic swimmers quite low and on the order of 10^−15^, indicating that a substantial amount of energy is used to maintain the gradients rather than propelling the particle. Since *U* is held fixed, the output power *P*_out_ = 6*πηaU*^2^ is constant across all modes, so maximizing efficiency is equivalent to minimizing the input power *P*_in_ (see Supplementary Note S6.2 and S6.3). Remarkably, the pusher, despite producing the lowest swimming speed at constant incident laser power (Fig. 4c), emerges as the most efficient mode at constant propulsion velocity, requiring the smallest thermal power input (or minimal conversion of light into heat) to sustain a given speed. This counterintuitive result points to fundamental physical constraints governing thermophoretic propulsion efficiency that differ from purely hydrodynamic considerations.

## Discussion

In this study, we demonstrate real-time reconfiguration of hydrodynamic flow fields at microswimmers’ surfaces, enabling reversible switching between puller, pusher, and neutral squirmer modes for adaptivity studies. Through precise manipulation of these boundary flows, we find that pusher swimmers achieve the highest swimming efficiency with *β* ≈ − 1 emerging as the optimal configuration. Our finding does not contradict conventional hydrodynamic predictions but reflects a different energetic accounting. Standard squirmer theory identifies the spherical neutral squirmer (*β* = 0) as optimal because it minimizes the bulk viscous dissipation while still producing propulsion^12–14,42^; Sabass and Seifert further showed that for surface-driven swimmers the dominant fraction of this dissipation localizes in the interfacial boundary layer where the slip is generated^43^. Our efficiency instead takes the total absorbed power *P*_in_ = 4*πa*^2^*q*_0_ as the energetic cost, which by energy conservation captures all dissipative channels, namely bulk viscous, boundary-layer, and thermal relaxation to the bath. An analogous total-input perspective underlies the chemical-swimmer efficiency of Kreissl et al. ^44^, who likewise found that localized rear activity (a pusher-like mode) is optimal. The squirmer optimum at *β* = 0 and the absorbed-power optimum at *β* ≈ − 1 therefore minimize different energetic quantities and should be read as complementary rather than in direct conflict. Similarly, size and shape-optimization studies focus on geometric considerations to tune efficiency while treating surface activity distributions as independently tunable parameters^13,41,43^.

The resolution of this apparent paradox lies in recognizing that physical constraints fundamentally alter the optimization landscape. For thermophoretic swimmers, the requirement that heat source density remains positive everywhere on the particle surface, i.e. *q*(*θ*)≥0 for all *θ*, introduces a constraint that couples all higher multipole coefficients to the monopole in the surface heat distribution. This coupling is absent in idealized models where surface slip velocities can be prescribed arbitrarily. Conversely, only propulsion mechanisms that allow sign-changing surface activity and require globally balanced activity recover the unconstrained *β* = 0 optimum (for example self-electrophoresis with a net-zero ion flux). While previous work has explored how geometric shape affects efficiency through modified resistance tensors^42^, our results reveal that realizability constraints on the driving fields themselves impose a more fundamental limitation: they determine not only the magnitude of efficiency but also which swimming mode is optimal. The optimal swimmer at β^*^ ≈ − 1 does not imply that the pusher flow field is hydrodynamically optimal in the given setting. Rather, the pusher minimizes the monopole heating contribution *q*_0_, thereby reducing energy dissipated into background heating that does not contribute to propulsion. The pusher mode thus represents the energetically least expensive way to generate a prescribed propulsion velocity.

In the quadrupole approximation, the constraint *q*(*θ*)≥0 forces a specific relationship between the monopole heating *q*_0_, the propulsion-driving dipole *q*_1_, and the mode-selecting quadrupole *q*_2_. To maintain positive heating everywhere, the monopole term must compensate for the minimum of the combined dipole-quadrupole distribution, creating two distinct regimes (linear: *q*_2_ < − *q*_1_/3 with *q*_0_ = − *q*_1_ − *q*_2_; nonlinear: *q*_2_ > − *q*_1_/3 with ${q}_{0}=\frac{{q}_{2}}{2}+\frac{{q}_{1}^{2}}{6{q}_{2}}$). The resulting swimming efficiencies in these regimes are (see Supplementary Note S6):

4 $${\epsilon }_{{{{\rm{lin}}}}}=\frac{2\eta {\chi }^{2}}{3a{T}_{0}^{2}{({\kappa }_{{{{\rm{in}}}}}+2{\kappa }_{{{{\rm{out}}}}})}^{2}}\frac{{q}_{1}^{2}}{(-{q}_{1}-{q}_{2})}\,,$$

5 $${\epsilon }_{{{{\rm{non-lin}}}}}=\frac{4\eta {\chi }^{2}}{3a{T}_{0}^{2}{({\kappa }_{{{{\rm{in}}}}}+2{\kappa }_{{{{\rm{out}}}}})}^{2}}\frac{{q}_{1}^{2}}{\left(\frac{{q}_{1}^{2}}{3{q}_{2}}+{q}_{2}\right)}\,,$$

where *η* is the fluid viscosity, *χ* is the thermo-osmotic mobility, *T*_0_ is the ambient temperature, *a* is the particle radius, and *κ*_in_ and *κ*_out_ are the thermal conductivities inside and outside the particle. These expressions reveal how the constraint-induced coupling manifests: efficiency depends not on *q*_1_ and *q*_2_ independently, but on *q*_1_ and their ratio, which determines *β* = *B*_2_/∣*B*_1_∣ ∝ *q*_2_/*q*_1_. Optimizing efficiency with respect to this ratio for a given swimming speed (fixed *q*_1_) yields ${\beta }^{*}=-\sqrt{3}\frac{{\kappa }_{{{{\rm{in}}}}}+2{\kappa }_{{{{\rm{out}}}}}}{2{\kappa }_{{{{\rm{in}}}}}+3{\kappa }_{{{{\rm{out}}}}}}\approx -1$ for typical material parameters (see Supplementary Note S6.2 for parameters), precisely where we observe maximum experimental efficiency (Fig. 4e).

Importantly, the pusher optimum (β^*^ ≈ − 1) is not an artifact of our second-order Legendre truncation. When we extend the theoretical analysis to arbitrary multipole orders while maintaining the positivity constraint *q*(*θ*)≥0, the optimal efficiency is achieved in the limit of delta-function-like localized heating at the rear pole of the particle (leading to ${\beta }_{\infty }^{*}\approx -3$ and an efficiency increase by a factor of $\sqrt{3}$, Supplementary Note S6.3) analogous to the optimal source found in reference^44^ for chemical swimmers. This point-like heating naturally corresponds to a pusher mode, confirming that the constraint-induced selection of β^*^ ≈ − 1 (${\beta }_{\infty }^{*}\approx -3$) is a fundamental feature of thermophoretic propulsion under physical realizability constraints, independent of the mathematical representation. More generally, we expect that any physical constraint restricting the realizable surface activity can modify the efficiency landscape. The specific optimum will depend on the nature of the constraint; for example, charge-conservation constraints in chemically powered phoretic swimmers have likewise been shown to alter the optimal activity distribution and swimming state^44^. Our second-order truncation captures this essential physics while remaining experimentally accessible, since thermal diffusion and optical resolution limit the creation of perfectly localized heating in practice.

This finding suggests a broader principle: physical realizability constraints may universally shift optimal swimming modes away from hydrodynamic or thermodynamic predictions. At the same time, swimmer optimality is not universal but inherently context-dependent, shaped by the propulsion mechanism, environmental rheology, swimmer interactions, confinement, and the chosen efficiency measure^45^. Previous studies have shown that viscoelastic environments can modify swimmer efficiencies^46^ and that biological swimmers are not necessarily optimized solely with respect to hydrodynamic dissipation^7^. These results emphasize that the propulsion mechanism itself plays a central role in determining energetic costs and therefore the notion of optimality.

An intriguing open question is whether this β^*^ ≈ − 1 optimum is specific to spherical thermophoretic swimmers, or represents a more general feature. For non-spherical swimmers, three scenarios are possible: (1) *Universal selection*, all phoretic swimmers under realizability constraints converge to β^*^ ≈ − 1 regardless of shape, with geometry affecting only absolute efficiency through resistance tensors; (2) *Shape-dependent optima*, different geometries yield different optimal *β* values as constraint-induced coupling competes with geometric effects; or (3) *Constraint relaxation*, highly asymmetric shapes possess sufficient degrees of freedom to approach neutral swimming while satisfying *q*≥0. Our optical manipulation platform enables direct experimental testing of these scenarios by creating “effective shape asymmetries” through illumination pattern variations. Extending our analytical framework to arbitrary axisymmetric shapes requires solving the coupled optimization of power, temperature field, and shape under the combined constraints of heat equation, positivity, and fixed volume, an important direction for future theoretical work.

Beyond efficiency optimization, the demonstrated real-time reconfigurability enables functional versatility, as the microswimmer can reversibly switch between acting as a propeller, a pump (generating force-dipole flows for shakers with *β* = ± *∞*), or a stealth swimmer (with source-dipole-dominated flows for *β* = 0). This ability to select the hydrodynamic mode could be useful for tasks in specific environments, for example swimming in viscoelastic media^8^. Depending on the selected mode, the swimmer exhibits distinct hydrodynamic interactions with surfaces or other active components^47–49^, with pushers naturally clearing paths by leaving empty space behind while pullers create trailing structures. In the present work the hydrodynamic mode is selected by the operator, and the external feedback loop detects only the position of the particle and updates the position and orientation of the projected illumination pattern accordingly. A natural extension is to bring the hydrodynamic mode under the same closed-loop, reinforcement-learning control recently demonstrated for this thermoplasmonic swimmer platform^50^, so that the autonomous system rather than the operator selects the mode in response to its environment, while on-board control of the kind found in biological swimmers remains for future work.

Our work opens the possibility of experimentally exploring the adaptation of microswimmers in complex environments, their efficiencies and morphological developments which are highly dependent on external physical constraints. By demonstrating that physical constraints on the propulsion mechanism rather than geometry alone determine optimal swimming efficiency, our findings reveal a distinct pathway for evolutionary optimization in synthetic active matter: iterative refinement of programmable driving field patterns offers experimental access to constraint-guided adaptive dynamics without requiring morphological changes, complementing traditional shape-optimization approaches with a mechanism-optimization paradigm that may be more readily accessible in synthetic systems. More broadly, our results establish a framework for morphological optimization in active matter, where performance landscapes are determined by the interplay between hydrodynamic efficiency and mechanism-specific constraints. This perspective connects naturally to the concept of morphological intelligence, the idea that an agent’s body and physical interaction with its environment can offload computation to the underlying physics. Of its many facets, our work addresses the hydrodynamic morphology: the effective shape of the swimmer is given by the surface flow it generates rather than by its fixed geometric form, so that adaptive function can emerge from controlling the flow field rather than from changing the particle’s material structure, opening new directions for embodied, constraint-shaped adaptive strategies in active matter.

## Methods

### Sample preparation

We use melamine formaldehyde (MF) particles (microParticles GmbH, radius *a* = 2.2 μm) coated with 10 nm diameter gold nanoparticles on 10 % of the particle surface. The sample consists of a thin water film (*h* = 5 μm for swimming, and *h* = 4.4 μm for flow field measurements) confined between two microscopy slides. We seal the sample with polydimethylsiloxane to prevent evaporation. For tracing the flow fields, we add gold nanoparticles (Sigma-Aldrich, diameter 250 nm) to the sample solution.

### Optical microscopy

We observe particle motion with an inverted microscope (Olympus IX71) under dark field illumination using an oil-immersion dark field condenser (Olympus, NA = 1.2) and an oil-immersion objective (Olympus UPlanApo  × 100/0.6). We record images with a sCMOS camera (Hamamatsu ORCA Flash) at an exposure time of 33 ms.

Particle image velocimetry (PIV) is used to extract velocities and to calculate streamlines (see Supplementary Note S4 for details). For the flow field measurements shown in Fig. 3, each squirmer parameter was measured using 5–11 different particles. For every individual measurement, tracer trajectories were recorded over approximately 4000 consecutive images. With an inverse frame time of 33 ms, this corresponds to a recording duration of about 132 s ( ≈ 2.2 min) per measurement.

For the swimming speed measurements, data were recorded from five different particles for the neutral squirmer and from three different particles for both the pusher and puller modes at each laser power. Each measurement consisted of approximately 5 000 − 10, 000 recorded images, corresponding to trajectory durations of approximately 3 − 6 min per measurement. Error bars represent the standard error obtained from measurements on different particles.

For the temperature measurements, data were recorded from five different particles for each squirmer parameter. The error bars represent the standard error obtained from the independently measured temperature profiles.

### Generation of laser light pattern

We create distinct laser light profiles by shaping the laser light wavefront using a spatial light modulator (SLM). A 532 nm single-mode DPSS laser beam passing through a lambda half plate is expanded to illuminate the entire field of view of the SLM (512 × 512 px). The SLM modulates the phase of the incoming linearly polarized light between 0 and 2*π*. A phase image for *N* spatially distributed Gaussian spots is projected onto the SLM, which is positioned in the Fourier plane of the optical setup. We optimize both the number *N* and the spatial arrangement of these Gaussian spots using a custom algorithm. During a Monte Carlo optimization, the goal is to generate an intensity profile that closely approximates the theoretical prediction shown in Supplementary Figs. S2 and S3. To quantify the match, we compute the mean squared error between the experimentally measured laser field and the theoretical intensity profile. The optimization terminates when this deviation falls below a predefined threshold of 0.001 or after a maximum of 40,000 iterations. More details on the algorithm are provided in Supplementary Note S2.

After scattering from the SLM surface, the light passes through a beam block positioned in the Fourier plane of the optical setup. The beam block removes the zeroth diffraction order originating from the pixelated structure of the SLM. An iris placed in the image plane crops the beam and suppresses unwanted illumination outside the target region. The pattern is then focused into the back focal plane of the oil-immersion objective (Olympus UPlanApo  × 100/0.6), illuminating the sample plane.

To generate a moving thermo-phoretic microswimmer, we apply a steering method following previous studies^25,36,51^. A custom LabVIEW program integrates all hardware components into a real-time feedback system. Camera-acquired images are directly fed into a Python program embedded in LabVIEW within approximately 20 ms. An intensity-based feature detection module analyzes the images and returns the *x*- and *y*-coordinates of detected particles within 5 ms. We compare the particle’s coordinates to a predefined target position, which dictates the position and orientation of the light pattern projected onto the particle’s surface to generate thermo-phoretic motion. In the next step, the phase mask of the light pattern is computed and sent to the SLM. This step also allows selection of the specific light pattern and thus the desired propulsion mode. In the consecutive camera frame, the particle’s coordinates are reanalyzed and compared to the target, forming a feedback loop that updates the position and orientation of the projected light pattern.

## Supplementary information

## Data Availability

The data for the figures is made available on figshare https://figshare.com/s/b349d86e3d83e45266f4.

## Code availability

The codes used in this study are available on figshare via https://figshare.com/s/b349d86e3d83e45266f4.

## References

[1] Lighthill, M. J. On the squirming motion of nearly spherical deformable bodies through liquids at very small Reynolds numbers. *Commun. Pure Appl. Math.* **5**, 109–118 (1952).

[2] Blake, J. R. A spherical envelope approach to ciliary propulsion. *J. Fluid Mech.* **46**, 199–208 (1971).

[3] Bechinger, C. et al. Active particles in complex and crowded environments. *Rev. Mod. Phys.* **88**, 045006 (2016).

[4] Drescher, K., Dunkel, J., Cisneros, L. H., Ganguly, S. & Goldstein, R. E. Fluid dynamics and noise in bacterial cell–cell and cell–surface scattering. *PNAS* **108**, 10940–10945 (2011). 21690349 10.1073/pnas.1019079108 PMC3131322

[5] Hu, J., Yang, M., Gompper, G. & Winkler, R. G. Modelling the mechanics and hydrodynamics of swimming e. coli. *Soft Matter* **11**, 7867–7876 (2015). 26256240 10.1039/c5sm01678a

[6] Lopez, D. & Lauga, E. Dynamics of swimming bacteria at complex interfaces. *Phys. Fluids* **26**, 071902 (2014).

[7] Klindt, G. S. & Friedrich, B. M. Flagellar swimmers oscillate between pusher-and puller-type swimming. *Phys. Rev. E* **92**, 063019 (2015). 10.1103/PhysRevE.92.063019 26764816

[8] Qi, K., Zhou, Y., Corato, M. D., Stratford, K. & Pagonabarraga, I. Unravel the rotational and translational behavior of a single squirmer in flexible polymer solutions at different Reynolds numbers. *Commun. Phys.* **8**, 487 (2025). 41341223 10.1038/s42005-025-02391-9 PMC12669044

[9] Zayed, R. M. A., Ardekani, A. M. & Nourhani, A. Swimmer types of optimum surface-driven active particles. *J. Fluid Mech.* **1009**, R1 (2025).

[10] Osterman, N. & Vilfan, A. Finding the ciliary beating pattern with optimal efficiency. *PNAS* **108**, 15727–15732 (2011). 21896741 10.1073/pnas.1107889108 PMC3179098

[11] Drescher, K. et al. Dancing volvox: hydrodynamic bound states of swimming algae. *Phys. Rev. Lett.* **102**, 168101 (2009). 19518757 10.1103/PhysRevLett.102.168101 PMC4833199

[12] Daddi-Moussa-Ider, A., Nasouri, B., Vilfan, A. & Golestanian, R. Optimal swimmers can be pullers, pushers or neutral depending on the shape. *J. Fluid Mech.* **922**, R5 (2021).

[13] Michelin, S. & Lauga, E. Efficiency optimization and symmetry-breaking in a model of ciliary locomotion. *Phys. Fluids* **22**, 111901 (2010).

[14] Nasouri, B., Vilfan, A. & Golestanian, R. Minimum dissipation theorem for microswimmers. *Phys. Rev. Lett.* **126**, 034503 (2021). 33543965 10.1103/PhysRevLett.126.034503

[15] Stone, H. A. & Samuel, A. D. Propulsion of microorganisms by surface distortions. *Phys. Rev. Lett.* **77**, 4102 (1996). 10062388 10.1103/PhysRevLett.77.4102

[16] Constantino, M. A. et al. Bipolar lophotrichous helicobacter suis combine extended and wrapped flagella bundles to exhibit multiple modes of motility. *Sci. Rep.* **8**, 14415 (2018). 30258065 10.1038/s41598-018-32686-7 PMC6158295

[17] Ishikawa, T. Suspension biomechanics of swimming microbes. *J. R. Soc. Interface* **6**, 815–834 (2009). 19674997 10.1098/rsif.2009.0223 PMC2838254

[18] Howse, J. R. et al. Self-motile colloidal particles: From directed propulsion to random walk. *Phys. Rev. Lett.* **99**, 048102 (2007). 17678409 10.1103/PhysRevLett.99.048102

[19] Zhang, T. et al. Janus particles with tunable patch symmetry and their assembly into chiral colloidal clusters. *Nat. Commun.* **14**, 8494 (2023). 38129397 10.1038/s41467-023-44154-6 PMC10739893

[20] Ibrahim, Y., Golestanian, R. & Liverpool, T. B. Multiple phoretic mechanisms in the self-propulsion of a pt-insulator janus swimmer. *J. Fluid Mech.* **828**, 318–352 (2017).

[21] Popescu, M. N., Uspal, W. E. & Dietrich, S. Self-diffusiophoresis of chemically active colloids. *Eur. Phys. J. Spec. Top.* **225**, 2189–2206 (2016).

[22] Theurkauff, I., Cottin-Bizonne, C., Palacci, J., Ybert, C. & Bocquet, L. Dynamic clustering in active colloidal suspensions with chemical signaling. *Phys. Rev. Lett.* **108**, 268303 (2012). 23005020 10.1103/PhysRevLett.108.268303

[23] Bickel, T., Majee, A. & Würger, A. Flow pattern in the vicinity of self-propelling hot Janus particles. *Phys. Rev. E* **88**, 012301 (2013). 10.1103/PhysRevE.88.012301 23944457

[24] Jiang, H.-R., Yoshinaga, N. & Sano, M. Active motion of a Janus particle by self-thermophoresis in a defocused laser beam. *Phys. Rev. Lett.* **105**, 268302 (2010). 21231718 10.1103/PhysRevLett.105.268302

[25] Fränzl, M., Muinos-Landin, S., Holubec, V. & Cichos, F. Fully steerable symmetric thermoplasmonic microswimmers. *ACS Nano* **15**, 3434–3440 (2021). 33556235 10.1021/acsnano.0c10598

[26] Shields, C. W. & Velev, O. D. The evolution of active particles: toward externally powered self-propelling and self-reconfiguring particle systems. *Chem* **3**, 539–559 (2017).

[27] Yan, J. et al. Reconfiguring active particles by electrostatic imbalance. *Nat. Mater.* **15**, 1095–1099 (2016). 27400388 10.1038/nmat4696

[28] Ketzetzi, S. et al. Active colloidal molecules with dynamic configurational freedom. *ACS Nano* **19**, 29430–29439 (2025). 40787951 10.1021/acsnano.5c07142 PMC12369011

[29] Vutukuri, H. R., Lisicki, M., Lauga, E. & Vermant, J. Light-switchable propulsion of active particles with reversible interactions. *Nat. Commun.* **11**, 2628 (2020). 32457438 10.1038/s41467-020-15764-1 PMC7251099

[30] Lee, J. G., Brooks, A. M., Shelton, W. A., Bishop, K. J. & Bharti, B. Directed propulsion of spherical particles along three dimensional helical trajectories. *Nat. Commun.* **10**, 2575 (2019). 31189873 10.1038/s41467-019-10579-1 PMC6561940

[31] Singh, A. V. et al. Mechanical coupling of puller and pusher active microswimmers influences motility. *Langmuir* **36**, 5435–5443 (2020). 32343587 10.1021/acs.langmuir.9b03665 PMC7304893

[32] Grober, D. et al. Unconventional colloidal aggregation in chiral bacterial baths. *Nat. Phys.* **19**, 1680–1688 (2023).

[33] Chakraborty, I. et al. Self-assembly dynamics of reconfigurable colloidal molecules. *ACS Nano* **16**, 2471–2480 (2022). 35080387 10.1021/acsnano.1c09088 PMC8867909

[34] Grauer, J. et al. Active droploids. *Nat. Commun.* **12**, 6005 (2021). 34650040 10.1038/s41467-021-26319-3 PMC8516867

[35] Ben Zion, M. Y., Fersula, J., Bredeche, N. & Dauchot, O. Morphological computation and decentralized learning in a swarm of sterically interacting robots. *Sci. Robot.* **8**, eabo6140 (2023). 36812334 10.1126/scirobotics.abo6140

[36] Khadka, U., Holubec, V., Yang, H. & Cichos, F. Active particles bound by information flows. *Nat. Commun.* **9**, 3864 10.1038/s41467-018-06445-1 (2018). 30242284 PMC6154969

[37] Bregulla, A. P., Würger, A., Günther, K., Mertig, M. & Cichos, F. Thermo-osmotic flow in thin films. *Phys. Rev. Lett.* **116**, 188303 (2016). 27203347 10.1103/PhysRevLett.116.188303

[38] Bregulla, A. P. & Cichos, F. Flow fields around pinned self-thermophoretic microswimmers under confinement. *J. Chem. Phys.* **151**, 044706 (2019). 31370563 10.1063/1.5088131

[39] Fränzl, M. & Cichos, F. Hydrodynamic manipulation of nano-objects by optically induced thermo-osmotic flows. *Nat. Commun.* **13**, 656 (2022). 35115502 10.1038/s41467-022-28212-z PMC8813924

[40] Horn, R. G. Refractive indices and order parameters of two liquid crystals. *J. de. Phys.* **39**, 105–109 (1978).

[41] Bregulla, A. P. & Cichos, F. Size dependent efficiency of photophoretic swimmers. *Faraday Discuss.* **184**, 381–391 (2015). 26402861 10.1039/c5fd00111k

[42] Daddi-Moussa-Ider, A., Golestanian, R. & Vilfan, A. Minimum entropy production by microswimmers with internal dissipation. *Nat. Commun.* **14**, 6060 (2023). 37770449 10.1038/s41467-023-41280-z PMC10539332

[43] Sabass, B. & Seifert, U. Efficiency of surface-driven motion: Nanoswimmers beat microswimmers. *Phys. Rev. Lett.* **105**, 218103 (2010). 21231358 10.1103/PhysRevLett.105.218103

[44] Kreissl, P., Holm, C. & De Graaf, J. The efficiency of self-phoretic propulsion mechanisms with surface reaction heterogeneity. *J. Chem. Phys*. **144**, 204902 (2016). 10.1063/1.4951699 27250326

[45] Bailey, M. R. et al. Low efficiency of janus microswimmers as hydrodynamic mixers. *Phys. Rev. E* **110**, 044601 (2024). 39562881 10.1103/PhysRevE.110.044601

[46] Zhu, L., Lauga, E. & Brandt, L. Self-propulsion in viscoelastic fluids: Pushers vs. pullers. *Phys. Fluids* **24**, 051902 (2012).

[47] Shen, Z., Würger, A. & Lintuvuori, J. S. Hydrodynamic interaction of a self-propelling particle with a wall: Comparison between an active Janus particle and a squirmer model. *Eur. Phys. J. E* **41**, 39 (2018). 29594924 10.1140/epje/i2018-11649-0

[48] Zöttl, A. & Stark, H. Hydrodynamics determines collective motion and phase behavior of active colloids in quasi-two-dimensional confinement. *Phys. Rev. Lett.* **112**, 118101 (2014). 24702421 10.1103/PhysRevLett.112.118101

[49] Rohde, L., Quinn, D., Paul, D. & Cichos, F. Regulated polarization of active particles in local osmotic flow fields. *Commun. Phys.* **8**, 213 (2025).

[50] Paul, D., Milosevic, N., Scherf, N. & Cichos, F. Physical embodiment enables information processing beyond explicit flow sensing in active matter. *Sci. Adv.* **12**, eaec0783 (2026). 41824563 10.1126/sciadv.aec0783 PMC12985729

[51] Wang, X., Chen, P.-C., Kroy, K., Holubec, V. & Cichos, F. Spontaneous vortex formation by microswimmers with retarded attractions. *Nat. Commun.* **14**, 56 (2023). 36599830 10.1038/s41467-022-35427-7 PMC9813373

