# Supplementary material for: Programmable hydrodynamics of active particles
Source: Nat Commun. 2026 Sep 2;17:9406. doi: 10.1038/s41467-026-77281-x (PMC13538562; doi:10.1038/s41467-026-77281-x)
Supplement: Supplementary file 1 [file 41467_2026_77281_MOESM1_ESM.pdf]

Supplementary Notes

Programmable Hydrodynamics of Active Particles

Lisa Rohde[1], Gordei Anchutkin[1], Viktor Holubec[2], and Frank Cichos[1,*]

[1] *Molecular Nanophotonics Group, Peter Debye Institute for Soft Matter Physics, Leipzig University, 04103 Leipzig, Germany.*
[2] *Department of Macromolecular Physics, Faculty of Mathematics and Physics,Charles University, 18000 Prague, Czech Republic.* * *cichos@physik.uni-leipzig.de*

Contents

S1 Squirmer temperature profiles **2**
S1.1 Analytical derivation of temperature profiles . . . 2
S1.1.1 Quadrupole approximation of heat flux density and temperature fields . . . 3
S1.2 Connection to Squirmer model . . . 4
S1.2.1 Thermo-osmotic slip velocity . . . 4
S1.2.2 Squirmer surface velocities . . . 5
S1.3 Positivity constraints in the quadrupole approximation . . . 6
S1.3.1 Temperature fields for constrained heat sources in the quadrupole approximation . . . 7

S2 Experimental implementation **8**
S2.1 Experimental setup . . . 8
S2.2 Intensity pattern generation . . . 8
S2.3 Experimental intensity profiles . . . 10
S2.4 Refraction of illumination patterns . . . 10

S3 Experimental measurements of surface temperatures **13**
S3.1 Measurement principle . . . 13
S3.2 Heating contributions and surface temperatures . . . 14

S4 Particle Image Velocimetry **14**

S5 Simulation of hydrodynamic flow fields **16**

S6 Swimming efficiency **18**
S6.1 Constraints to swimming efficiency in the quadrupole approximation . . . 19
S6.2 Maximum swimming efficiency in the quadrupole approximation . . . 20
S6.3 Optimal heating and maximum efficiency beyond the quadrupole approximation . . . 20
S6.3.1 Maximum efficiency at fixed output power . . . 21
S6.3.2 Maximum efficiency at fixed input power . . . 22
S6.3.3 Optimality when a cooling is possible . . . 22

S1 Squirmer temperature profiles

The microswimmer is illuminated with a spatially varying light pattern. The particle absorbs the light and creates a non-uniform heat source density $q(\theta)$ that establishes temperature gradients on the surface. These gradients create thermo-osmotic flows that propel the particle through the fluid. The swimming mechanism lies in the interplay between the imposed surface heating and the resulting temperature distribution. The full description of the temperature field inside the particle ($r < a$) with thermal conductivity κ_{in} and outside of the particle ($r > a$) with thermal conductivity κ_{out} will be analytically derived.

S1.1 Analytical derivation of temperature profiles

The temperature distribution in both the sphere and the surrounding medium obeys the steady-state heat equation, also known as the Laplace's equation:

$$\nabla^2 T = 0\,. \tag{S1}$$

This equation follows from the balance between heat conduction and the absence of internal heat sources or sinks within the bulk materials. Without loss of generality, we assume that the particle swims with a speed $U > 0$ along the positive z-axis. Owing to the symmetry of the particle, the temperature profile must be symmetric with respect to the propulsion axis. Consequently, the temperature can depend only on the azimuthal angle θ (measured from the z-axis) and on the distance r from the particle center. In spherical coordinates, the Laplace equation then reads:

$$\frac{1}{r^2}\frac{\partial}{\partial r}\left(r^2\frac{\partial T}{\partial r}\right) + \frac{1}{r^2\sin\theta}\frac{\partial}{\partial\theta}\left(\sin\theta\frac{\partial T}{\partial\theta}\right) = 0\,, \tag{S2}$$

which can be solved by using the separation of variables method. Assuming a solution of the form $T(r,\theta) = R(r)P(\theta)$, two ordinary differential equations are obtained:

$$r^2\frac{d^2R}{dr^2} + 2r\frac{dR}{dr} - l(l+1)R = 0\,, \tag{S3}$$

$$\sin\theta\frac{d}{d\theta}\left(\sin\theta\frac{dP}{d\theta}\right) + l(l+1)\sin^2\theta P = 0\,. \tag{S4}$$

The second equation yields Legendre polynomials $P_l(\cos\theta)$ as solutions. The radial equation has the general solutions:

$$R(r) = Ar^l + Cr^{-(l+1)}\,. \tag{S5}$$

Inside the sphere, $r < a$, the temperature must remain finite as $r \to 0$. This boundary condition constraints the solution to

$$T_{\text{in}} = T_0 + \sum_{l=0}^{\infty} A_l r^l P_l(\cos(\theta))\,, \tag{S6}$$

where the temperature at the particle center is given by $T_0 + A_0$. Outside the sphere, $r > a$, the temperature approaches the ambient temperature T_0 as $r \to \infty$ which gives the solution

$$T_{\text{out}} = T_0 + \sum_{l=0}^{\infty} C_l r^{-(l+1)} P_l(\cos(\theta))\,. \tag{S7}$$

The surface of the particle imposes boundary conditions that ensure both temperature continuity and heat flux balance. The temperature must be continuous across the interface,

$$T_{\text{in}}(a,\theta) = T_{\text{out}}(a,\theta)\,, \tag{S8}$$

and the heat fluxes inside and outside the particle must be balanced according to

$$\kappa_{\text{in}} \frac{\partial T_{\text{in}}}{\partial r} - \kappa_{\text{out}} \frac{\partial T_{\text{out}}}{\partial r} = q(\theta) \,. \tag{S9}$$

Here $q(\theta)$ represents the heat generated at the surface, and κ_{in} and κ_{out} are the thermal conductivities of the particle and the surrounding medium, respectively. We expand the surface heat flux density in terms of Legendre polynomials:

$$q(\theta) = \sum_{l=0}^{\infty} q_l P_l(\cos(\theta)) \,. \tag{S10}$$

The expansion coefficients q_l are constrained by the fact that our experimental setup allows only for heating and not cooling, i.e., $q(\theta) \geq 0$ for all θ. Additionally, the integral $2\pi a^2 \int_0^{\pi} q(\theta) \sin\theta \, d\theta = 4\pi a^2 q_0$ gives the overall heating power absorbed by the particle (for details, see Sec. S6), implying $q_0 > 0$.
Applying the boundary conditions yields:

$$A_l a^l = C_l a^{-(l+1)} \,, \tag{S11}$$

$$\kappa_{\text{in}} l A_l a^{l-1} + \kappa_{\text{out}} (l+1) C_l a^{-(l+2)} = q_l \,. \tag{S12}$$

Solving for the coefficients then yields:

$$A_l = \frac{q_l a^{-l+1}}{\kappa_{\text{in}} l + \kappa_{\text{out}}(l+1)} \,, \tag{S13}$$

$$C_l = \frac{q_l a^{l+2}}{\kappa_{\text{in}} l + \kappa_{\text{out}}(l+1)} \,. \tag{S14}$$

The complete temperature solutions are thus: Inside the particle ($r < a$):

$$T_{\text{in}}(r,\theta) = T_0 + \sum_{l=0}^{\infty} \frac{q_l a^{-l+1}}{\kappa_{\text{in}} l + \kappa_{\text{out}}(l+1)} r^l P_l(\cos\theta) \,. \tag{S15}$$

Outside the particle ($r > a$):

$$T_{\text{out}}(r,\theta) = T_0 + \sum_{l=0}^{\infty} \frac{q_l a^{l+2}}{\kappa_{\text{in}} l + \kappa_{\text{out}}(l+1)} r^{-(l+1)} P_l(\cos\theta) \,. \tag{S16}$$

Note that the coefficients A_l and C_l are temperature expansion coefficients, distinct from the squirmer mode amplitudes B_n introduced later. At the particle surface ($r = a$), we have:

$$T(a,\theta) = T_0 + \sum_{l=0}^{\infty} \frac{q_l a}{\kappa_{\text{in}} l + \kappa_{\text{out}}(l+1)} P_l(\cos\theta) \,. \tag{S17}$$

S1.1.1 Quadrupole approximation of heat flux density and temperature fields

For practical microswimmers, we truncate the expansion at $l = 2$. This captures the monopole term ($l = 0$) which corresponds to uniform heating and does not contribute to self-propulsion (q_0); the dipole term ($l = 1$) which presents a linear heating that creates the propulsion velocity (q_1); and the quadrupole term ($l = 2$) which sets the hydrodynamic signatures of the microswimmer (q_2). The quadrupole approximation of heat flux density then equals

$$q(\theta) = q_0 P_0(\cos\theta) + q_1 P_1(\cos\theta) + q_2 P_2(\cos\theta) = q_0 + q_1 \cos\theta + \frac{q_2}{2}(3\cos\theta^2 - 1) \,. \tag{S18}$$

For the temperature inside the particle ($r < a$), we obtain:

$$T_{\text{in}}(r,\theta) = T_0 + \frac{q_0 a}{\kappa_{\text{out}}} + \frac{q_1}{\kappa_{\text{in}} + 2\kappa_{\text{out}}} r\cos\theta + \frac{q_2}{2\kappa_{\text{in}} + 3\kappa_{\text{out}}} \frac{r^2}{a} \frac{1}{2}(3\cos^2\theta - 1)\,. \tag{S19}$$

For the temperature outside the particle ($r > a$), we obtain:

$$T_{\text{out}}(r,\theta) = T_0 + \frac{q_0 a^2}{\kappa_{\text{out}}}\left(\frac{1}{r}\right) + \frac{q_1 a^3}{\kappa_{\text{in}} + 2\kappa_{\text{out}}}\left(\frac{1}{r}\right)^2 \cos\theta + \frac{q_2 a^4}{2\kappa_{\text{in}} + 3\kappa_{\text{out}}}\left(\frac{1}{r}\right)^3 \frac{1}{2}(3\cos^2\theta - 1)\,. \tag{S20}$$

The temperature on the particle surface ($r = a$) is then equal to

$$T(a,\theta) = T_0 + \frac{q_0 a}{\kappa_{\text{out}}} + \frac{q_1 a}{\kappa_{\text{in}} + 2\kappa_{\text{out}}}\cos\theta + \frac{q_2 a}{2\kappa_{\text{in}} + 3\kappa_{\text{out}}}\frac{1}{2}(3\cos^2\theta - 1)\,. \tag{S21}$$

S1.2 Connection to Squirmer model

S1.2.1 Thermo-osmotic slip velocity

A thermo-phoretic active particle swims due to spatially varying temperate patterns on its surface. These gradients induce a slip velocity at the particle-fluid interface through thermo-phoresis. This slip velocity is directly proportional to the tangential temperature gradient:

$$u_s(\theta) = \frac{\chi}{T_0}\nabla_{\parallel} T(\theta) = \frac{\chi}{T_0}\frac{1}{a}\frac{\partial T}{\partial \theta}\bigg|_{r=a}, \tag{S22}$$

with a being the radius of the microswimmer and T_0 the ambient temperature. The thermo-osmotic mobility coefficient χ describes the interfacial iterations between the solid and the liquid.
From the general surface temperature in Eq. (S17), we obtain:

$$\frac{\partial T}{\partial \theta}\bigg|_{r=a} = \sum_{l=0}^{\infty} \frac{q_l a}{\kappa_{\text{in}} l + \kappa_{\text{out}}(l+1)} \frac{\partial P_l(\cos\theta)}{\partial \theta}\,. \tag{S23}$$

Considering $\frac{\partial P_l(\cos\theta)}{\partial\theta} = -\sin\theta \frac{dP_l(\cos\theta)}{d(\cos\theta)}$, the slip velocity thus becomes:

$$u_s(\theta) = -\sum_{l=0}^{\infty} \frac{\chi}{T_0} \frac{q_l}{\kappa_{\text{in}} l + \kappa_{\text{out}}(l+1)} \sin\theta \frac{dP_l(\cos\theta)}{d(\cos\theta)}\,. \tag{S24}$$

Quadrupole approximation of thermo-osmotic slip velocities Truncating the expansion at $l = 2$, we obtain the first few Legendre polynomials as:

- $P_1(\cos\theta) = \cos\theta$ gives $\frac{\partial P_1}{\partial\theta} = -\sin\theta$,
- $P_2(\cos\theta) = \frac{1}{2}(3\cos^2\theta - 1)$ gives $\frac{\partial P_2}{\partial\theta} = -3\cos\theta\sin\theta$.

The tangential temperature gradient at the particle surface (r=a) then yields:

$$\frac{\partial T}{\partial \theta}\bigg|_{r=a} = -\frac{q_1 a}{\kappa_{\text{in}} + 2\kappa_{\text{out}}}\sin\theta - \frac{q_2 a}{2\kappa_{\text{in}} + 3\kappa_{\text{out}}}(3\cos\theta\sin\theta)\,. \tag{S25}$$

Using the identity $\cos\theta\sin\theta = \frac{1}{2}\sin(2\theta)$, we obtain:

$$\frac{1}{a}\frac{\partial T}{\partial \theta}\bigg|_{r=a} = -\frac{q_1}{\kappa_{\text{in}} + 2\kappa_{\text{out}}}\sin\theta - \frac{3q_2}{2(2\kappa_{\text{in}} + 3\kappa_{\text{out}})}\sin(2\theta)\,. \tag{S26}$$

The slip velocity then follows from:

$$u_s(\theta) = \frac{\chi}{T_0}\frac{1}{a}\left(-\frac{q_1 a}{\kappa_{\text{in}} + 2\kappa_{\text{out}}}\sin\theta - \frac{q_2 a}{2\kappa_{\text{in}} + 3\kappa_{\text{out}}}(3\cos\theta\sin\theta)\right)\,. \tag{S27}$$

S1.2.2 Squirmer surface velocities

The squirmer model, introduced by Lighthill (1952) [1], describes swimming motion through prescribed surface velocities. The boundary conditions for a spherical swimmer are:

$$u_\theta(r=a,\theta) = \sum_{n=1}^{\infty} B_n V_n(\cos\theta)\,, \tag{S28}$$

$$u_r(r=a,\theta) = 0\,, \tag{S29}$$

where B_n are the squirmer mode amplitudes and $V_n(\cos\theta) = \frac{-2}{n(n+1)}\frac{\partial P_n(\cos\theta)}{\partial\theta} = \frac{2\sin\theta}{n(n+1)}\frac{dP_n(\cos\theta)}{d(\cos\theta)}$.
Comparing the thermo-osmotic slip velocity in Eq. (S24) with the squirmer model, we need:

$$B_n V_n(\cos\theta) = -\frac{\chi}{T_0}\frac{q_n}{\kappa_{\text{in}}n + \kappa_{\text{out}}(n+1)}\sin\theta\frac{dP_n(\cos\theta)}{d(\cos\theta)}\,. \tag{S30}$$

Substituting the expression for V_n:

$$B_n\frac{2\sin\theta}{n(n+1)}\frac{dP_n(\cos\theta)}{d(\cos\theta)} = -\frac{\chi}{T_0}\frac{q_n}{\kappa_{\text{in}}n + \kappa_{\text{out}}(n+1)}\sin\theta\frac{dP_n(\cos\theta)}{d(\cos\theta)}\,. \tag{S31}$$

This yields:

$$B_n = -\frac{\chi}{T_0}\frac{q_n}{\kappa_{\text{in}}n + \kappa_{\text{out}}(n+1)}\frac{n(n+1)}{2}\,. \tag{S32}$$

This establishes the general connection between heat source modes q_n and squirmer modes B_n, with the negative sign arising from the derivatives of the Legendre polynomials.

Quadrupole approximation of squirmer slip velocities Truncating the expansion at $n = 2$ yields the surface velocity of the squirmer model:

$$u_s(\theta) = B_1\sin(\theta) + \frac{1}{2}B_2\sin(2\theta)\,, \tag{S33}$$

with the coefficients

$$B_1 = -\frac{\chi}{T_0}\frac{q_1}{\kappa_{\text{in}} + 2\kappa_{\text{out}}}\,, \tag{S34}$$

$$B_2 = -\frac{3\chi}{T_0}\frac{q_2}{2\kappa_{\text{in}} + 3\kappa_{\text{out}}}\,. \tag{S35}$$

By using the connection between the heat source modes q_1, q_2 and the squirmer modes B_1, B_2 we can rewrite the surface temperature from Eq. (S21) as:

$$\begin{aligned} T(a,\theta) &= T_0 + \frac{T_0 a}{\chi}\left(-B_1\cos\theta - \frac{B_2}{3}\frac{1}{2}(3\cos^2 - 1)\right) + c \\ &= T_0 + \frac{T_0 a}{\chi}\left(-B_1\cos\theta - \frac{B_2}{2}\cos\theta^2 + \frac{B_2}{6}\right) + c\,, \end{aligned} \tag{S36}$$

with $c = \frac{q_0 a}{\kappa_{\text{out}}}$ setting the baseline heating.
The squirmer parameter β is then:

$$\beta = \frac{B_2}{|B_1|} = -\frac{3q_2}{|q_1|}\frac{\kappa_{\text{in}} + 2\kappa_{\text{out}}}{2\kappa_{\text{in}} + 3\kappa_{\text{out}}}\,. \tag{S37}$$

This relationship allows us to control the squirmer behavior by tuning the heat source coefficients q_1 and q_2. With appropriate thermal conductivities, we can achieve desired swimming characteristics.
The swimming speed of the particle follows from the squirmer theory as $U = (2/3)B_1$ which can be rewritten

as

$$U = -\frac{2}{3}\frac{\chi}{T_0}\frac{q_1}{\kappa_{\text{in}} + 2\kappa_{\text{out}}} \,. \tag{S38}$$

The dipole heating contribution, characterized by q_1, is therefore determining the swimming speed.

S1.3 Positivity constraints in the quadrupole approximation

The temperature profile in the quadrupole approximation depends on the multipole heating coefficients q_0, q_1, and q_2. Since the experimental setup only allows for heating and not cooling, the heat flux density $q(\theta)$ and, consequently, the temperature variation $\Delta T(r,\theta) = T(r,\theta) - T_0$ must be positive. The heat flux density can be expanded as

$$q(\theta) = q_0 + q_1 \cos\theta + \frac{q_2}{2}(3\cos\theta^2 - 1) \,. \tag{S39}$$

Let us now locate the minimum of $q(\theta)$. We assume forward propulsion along the positive z-axis, and thus $U > 0$ in Eq. (S38), which implies $q_1 < 0$. The derivative of the heat source density,

$$\frac{dq}{d\theta} = -q_1 \sin\theta - 3q_2 \cos\theta \sin\theta = 0 \,, \tag{S40}$$

thus yields critical points at $\theta_{\text{cr}} = 0, \pi$, and at $\theta_{\text{cr}} = \cos^{-1}\left(\frac{-q_1}{3q_2}\right)$, where θ is measured from the positive z-direction.
At $\theta_{\text{cr}} = 0$, the heat source density reads

$$q(\theta_{\text{cr}} = 0) = q_0 + q_1 + q_2 \geq 0 \,. \tag{S41}$$

Maximizing the swimming efficiency requires the overall heating q_0 is minimal for given q_1 and q_2 (for details, see Sec. S6), and thus we set $q(\theta_{\text{cr}} = 0) = 0$. This yields the constraint

$$q_0 = -q_1 - q_2 \,. \tag{S42}$$

If the position of minimal heating shifts away from $\theta_{\text{cr}} = 0$, it occurs at

$$\theta_{\text{cr}} = \cos^{-1}\left(\frac{-q_1}{3q_2}\right) \,, \tag{S43}$$

for which the corresponding heat source density is given by

$$q\left(\theta_{\text{cr}} = \cos^{-1}\left(\frac{-q_1}{3q_2}\right)\right) = q_0 - \frac{1}{6}\frac{q_1^2}{q_2} - \frac{q_2}{2} . \tag{S44}$$

Imposing $q(\theta_{\text{cr}}) = 0$ yields the relation

$$q_0 = \frac{q_2}{2} + \frac{1}{6}\frac{q_1^2}{q_2} \,, \tag{S45}$$

which implies $q_2 > 0$.
The second derivative of the heat flux density reads:

$$\frac{d^2 q}{d\theta^2} = 3q_2 - q_1 \cos\theta - 6q_2 \cos^2\theta \,. \tag{S46}$$

The condition $\left.\frac{d^2 q}{d\theta^2}\right|_{\theta=\theta_{\text{cr}}} > 0$ for a critical point θ_{cr} to be a minimum holds for $\theta_{\text{cr}} = 0$ when $q_2 < -q_1/3$, and for $\theta_{\text{cr}} = \cos^{-1}\left(\frac{-q_1}{3q_2}\right)$ when $q_2 > -q_1/3$.
Hence, two distinct regimes emerge, connecting the monopole (q_0), dipole (q_1), and quadrupole (q_2) contributions to the heat source density:

$$q_2 < \frac{-q_1}{3} \; : \; q_0 = -q_1 - q_2 \,, \tag{S47}$$

$$q_2 > \frac{-q_1}{3} \; : \; q_0 = \frac{q_2}{2} + \frac{1}{6}\frac{q_1^2}{q_2} \,. \tag{S48}$$

According to the relation between the coefficients q_0, q_1, and q_2, we call these regimes linear and non-linear, respectively. The corresponding heat source densities are

$$q_{\text{lin}}(\theta) = (-q_1 - q_2) + q_1 \cos\theta + \frac{q_2}{2}(3\cos\theta^2 - 1) \,, \tag{S49}$$

$$q_{\text{non-lin}}(\theta) = (\frac{q_2}{2} + \frac{1}{6}\frac{q_1^2}{q_2}) + q_1 \cos\theta + \frac{q_2}{2}(3\cos\theta^2 - 1) \,. \tag{S50}$$

In terms of the squirmer coefficients B_1 and B_2, we get:

$$B_2 > -\frac{\kappa_{\text{in}} + 2\kappa_{\text{out}}}{2\kappa_{\text{in}} + 3\kappa_{\text{out}}} B_1 \; : \; q_0 = -q_1 - q_2 \,, \tag{S51}$$

$$B_2 < -\frac{\kappa_{\text{in}} + 2\kappa_{\text{out}}}{2\kappa_{\text{in}} + 3\kappa_{\text{out}}} B_1 \; : \; q_0 = \frac{q_2}{2} + \frac{q_1^2}{6q_2} \,. \tag{S52}$$

S1.3.1 Temperature fields for constrained heat sources in the quadrupole approximation

With the heat source density constrained by the positivity conditions and by setting $q_1 < 0$ to ensure propulsion in the positive z-direction, explicit expressions for the resulting temperature fields can be derived. The corresponding solutions depend on the specific operating regime considered.

Linear regime: $\mathbf{q_2} < \frac{-\mathbf{q_1}}{\mathbf{3}}$: In the linear regime, the monopole heating q_0 is replaced by $q_0 = -q_1 - q_2$. Substitution fo this expression into the temperature solutions from Eqs. (S19), (S20) and (S21) yields the following results:

$$\begin{aligned}
T_{\text{in}}(r,\theta) &= T_0 + \frac{(-q_1 - q_2)a}{\kappa_{\text{in}}} + \frac{q_1}{\kappa_{\text{out}} + 2\kappa_{\text{out}}} r\cos\theta + \frac{q_2}{2\kappa_{\text{in}} + 3\kappa_{\text{out}}}\frac{r^2}{a}\frac{1}{2}(3\cos^2\theta - 1) \,, \\
T_{\text{out}}(r,\theta) &= T_0 + \frac{(-q_1 - q_2)a^2}{\kappa_{\text{out}}}\left(\frac{1}{r}\right) + \frac{q_1 a^3}{\kappa_{\text{in}} + 2\kappa_{\text{out}}}\left(\frac{1}{r}\right)^2 \cos\theta + \frac{q_2 a^4}{2\kappa_{\text{in}} + 3\kappa_{\text{out}}}\left(\frac{1}{r}\right)^3 \frac{1}{2}(3\cos^2\theta - 1) \,, \\
T(a,\theta) &= T_0 + \frac{(-q_1 - q_2)a}{\kappa_{\text{out}}} + \frac{q_1 a}{\kappa_{\text{in}} + 2\kappa_{\text{out}}}\cos\theta + \frac{q_2 a}{2\kappa_{\text{in}} + 3\kappa_{\text{out}}}\frac{1}{2}(3\cos^2\theta - 1) \,.
\end{aligned} \tag{S53}$$

Non-linear regime: $\mathbf{q_2} > \frac{-\mathbf{q_1}}{\mathbf{3}}$: In the non-linear regime, the monopole heating q_0 is replaced by $q_0 = \frac{q_2}{2} + \frac{q_1^2}{6q_2}$. Substitution fo this expression into the general temperature solutions from Eqs. (S19), (S20) and (S21) yields the following results:

$$\begin{aligned}
T_{\text{in}}(r,\theta) &= T_0 + \frac{(\frac{q_2}{2} + \frac{q_1^2}{6q_2})a}{\kappa_{\text{out}}} + \frac{q_1}{\kappa_{\text{in}} + 2\kappa_{\text{out}}} r\cos\theta + \frac{q_2}{2\kappa_{\text{in}} + 3\kappa_{\text{out}}}\frac{r^2}{a}\frac{1}{2}(3\cos^2\theta - 1) \,, \\
T_{\text{out}}(r,\theta) &= T_0 + \frac{(\frac{q_2}{2} + \frac{q_1^2}{6q_2})a^2}{\kappa_{\text{out}}}\left(\frac{1}{r}\right) + \frac{q_1 a^3}{\kappa_{\text{in}} + 2\kappa_{\text{out}}}\left(\frac{1}{r}\right)^2 \cos\theta + \frac{q_2 a^4}{2\kappa_{\text{in}} + 3\kappa_{\text{out}}}\left(\frac{1}{r}\right)^3 \frac{1}{2}(3\cos^2\theta - 1) \,, \\
T(a,\theta) &= T_0 + \frac{(\frac{q_2}{2} + \frac{q_1^2}{6q_2})a}{\kappa_{\text{out}}} + \frac{q_1 a}{\kappa_{\text{in}} + 2\kappa_{\text{out}}}\cos\theta + \frac{q_2 a}{2\kappa_{\text{in}} + 3\kappa_{\text{out}}}\frac{1}{2}(3\cos^2\theta - 1) \,.
\end{aligned} \tag{S54}$$

S2 Experimental implementation

S2.1 Experimental setup

Our experimental setup comprises an inverted optical microscope (Olympus IX71). The particles are observed using dark field illumination with an oil-immersion dark-field condenser (Olympus, NA 1.2) and an oil-immersion objective (Olympus UPlanApo ×100/0.6). A laser with a wavelength of $\lambda = 532$ nm is built into the optical path. Its wavefront is modulated by a spatial light modulator (SLM) to generate tailored illumination patterns creating the required heat source distributions $q(\theta)$. Figure S**1** shows the optical setup and the sample configuration. Gold nanoparticles (250 nm) trace the hydrodynamic flow field.

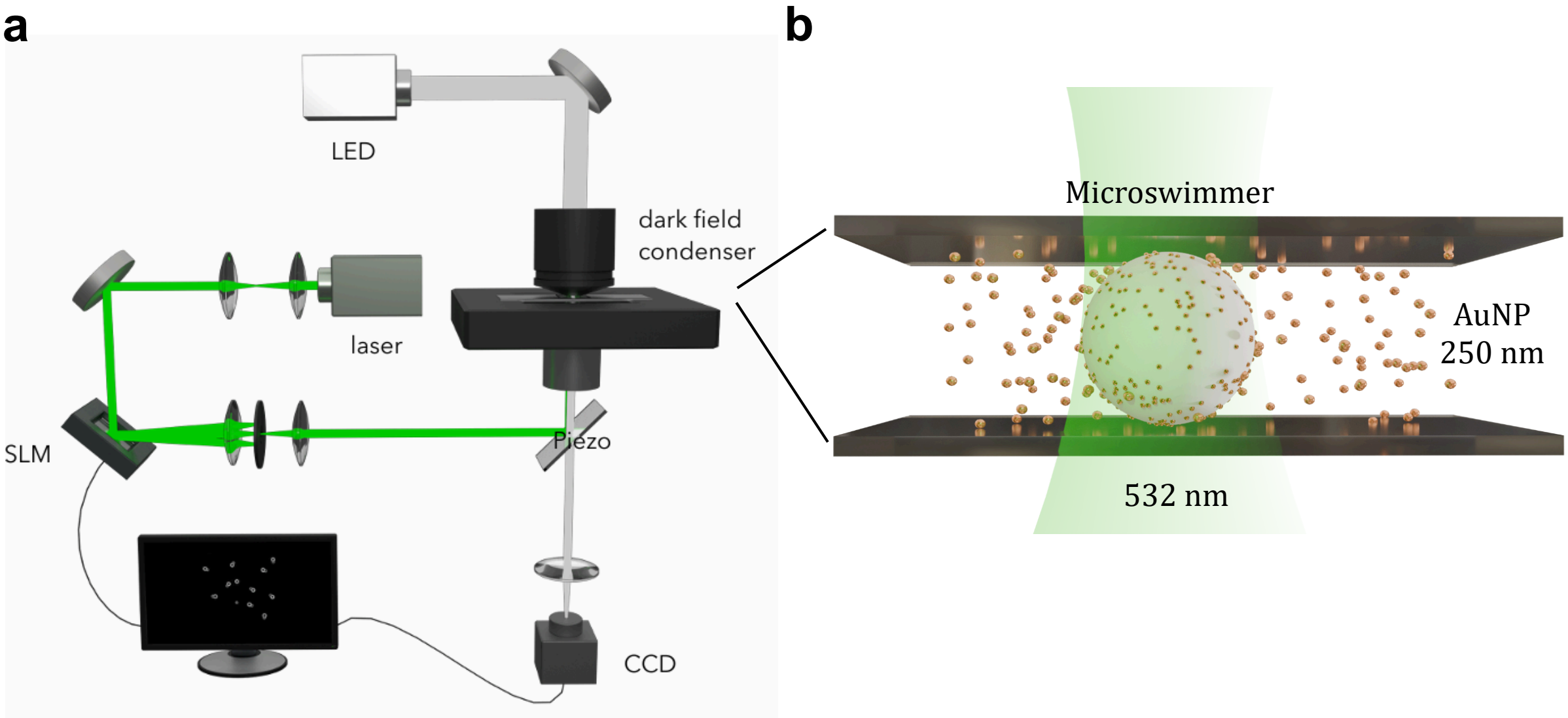

Fig. S1 Experimental setup. **a** Particle motion is observed with an inverted microscope under dark field illumination. The laser's wavefront is modulated with a spatial light modulator (SLM) to generate distinct illumination patterns. **b** The microswimmer is immobilized between two glass microscope slides. Gold nanoparticles (250 nm) trace the hydrodynamic flow field.

S2.2 Intensity pattern generation

The spatial light modulator (SLM) generates customized illumination patterns to produce the required heat source distribution. Since the absorbed laser intensity is proportional to local heat source density, we must create intensity patterns matching the theoretical $q(\theta)$.
The SLM projects a phase pattern that produces N Gaussian laser spots on the particle surface. To determine an optimized arrangement of these spots, we use a custom algorithm, illustrated in Fig. S**2**. A Monte Carlo optimization algorithm iteratively adjusts spot positions to minimize the mean squared error between realized and target intensity patterns:

$$\mathrm{MSE} = \frac{1}{N_{\mathrm{pix}}} \sum_{i=1}^{N_{\mathrm{pix}}} \left(I_i - I_{\mathrm{target},i}\right)^2 , \tag{S55}$$

where $I_{\mathrm{target},i} \propto q(\theta_i)$. The optimization terminates when MSE < 0.001 or after 40,000 iterations. The optimized phase image is sent to the SLM. An experimental image of the profile is recorded by utilizing the fluorescence of the dye (Nile Blue A, Sigma-Aldrich) at a wavelength of 532 nm. The MSE of this experimental image is then compared with that of the optimized algorithm-generated image. Next, the number N of Gaussian spots is increased, and the Monte Carlo algorithm is rerun. The value of N that yields an experimental image with the smallest deviation (MSE $< 10\,\%$) is ultimately selected for the measurements.

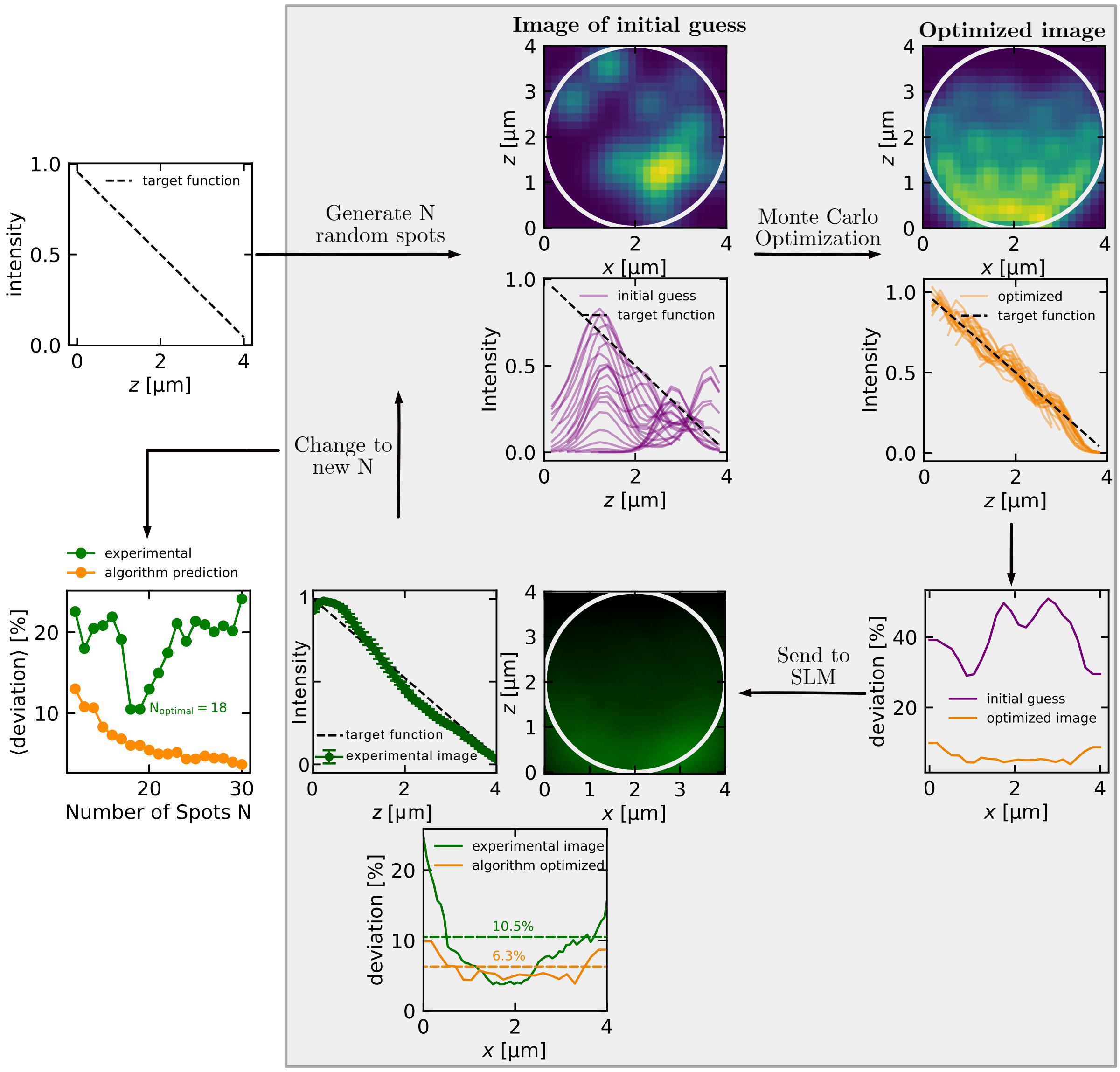

Fig. S2 Intensity pattern generation The SLM projects a phase pattern that produces N Gaussian laser spots on the particle surface. To obtain an optimized spot arrangement, a Monte Carlo algorithm is executed. The resulting optimized phase pattern is then displayed on the SLM, and an experimental image is recorded and analyzed. By varying N, we identify the phase pattern that reproduces the theoretical prediction within a margin of 10 %.

S2.3 Experimental intensity profiles

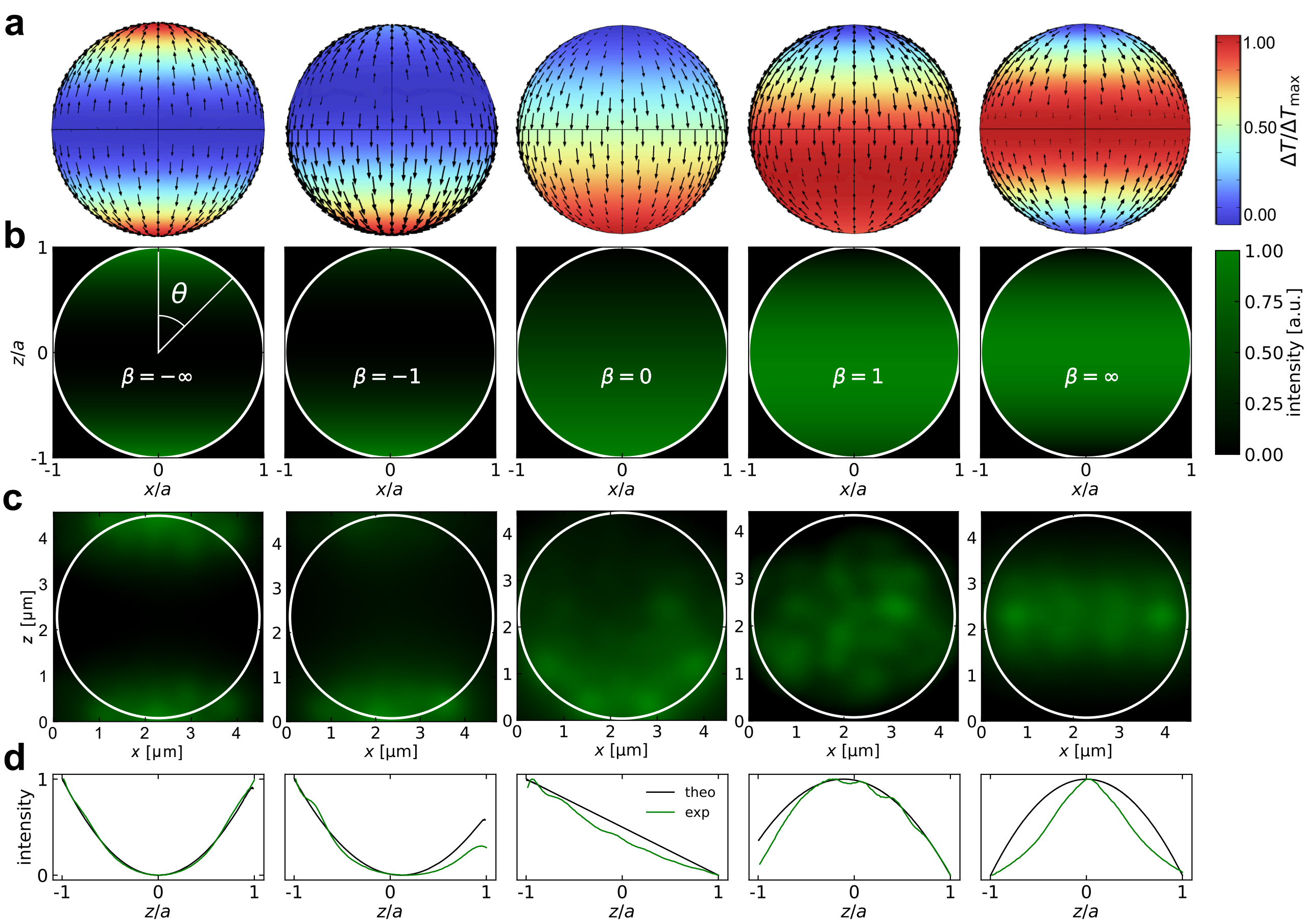

Fig. S3 Boundary flows and intensity profiles for different squirmer parameters. a The simulated boundary flows (black arrows) for different squirmer parameters are determined by the surface temperature distribution which is color coded. **b** Theoretical laser light intensity patterns for different squirmer parameters are computed following Eq. SS36. **c** Microscopy images of the experimental laser fields for different squirmer parameters are shown. They are measured by exploiting the fluorescence of a dye at the wavelength of $\lambda = 523\,\mathrm{nm}$ in the sample plane. **d** The intensity profiles along the z-direction closely align with theoretical predictions indicating precise control over the illumination.

Figure S**3** shows the theoretical and experimental intensity pattern for the negative shaker ($\beta = -\infty$), the pusher ($\beta = \infty$), the neutral squirmer ($\beta = 0$), the puller ($\beta = 1$) and the positive shaker ($\beta = \infty$). The light patterns are recorded by exploiting the fluorescence of the dye (Nile Blue A, Sigma-Aldrich) at a wavelength of $\lambda = 532\,\mathrm{nm}$. The intensity profiles along the z-direction closely align with theoretical predictions indicating precise control over the illumination (cf. Fig. S**3**d).
The simulated boundary flows on the surface of the particle are shown in Fig. S**3**a for different squirmer parameters β. They are determined by the color coded surface temperature profile obtained from a finite element simulation in COMSOL Multiphysics 6.1 by using the heat transfer module and the laminar flow module. Details on the simulation are given in Supplementary Note S5.

S2.4 Refraction of illumination patterns

In the experiment, the laser illuminates the particle from one side, so that only the hemisphere facing the beam is illuminated directly. Light transmitted through the particle is refracted at its interface and also deposits intensity on the shadowed (back) hemisphere. Since the particle diameter ($2a = 4.4\,\mu\mathrm{m}$) corresponds to about eleven optical wavelengths in water, the system lies between the ray- and wave-optics regimes. We therefore

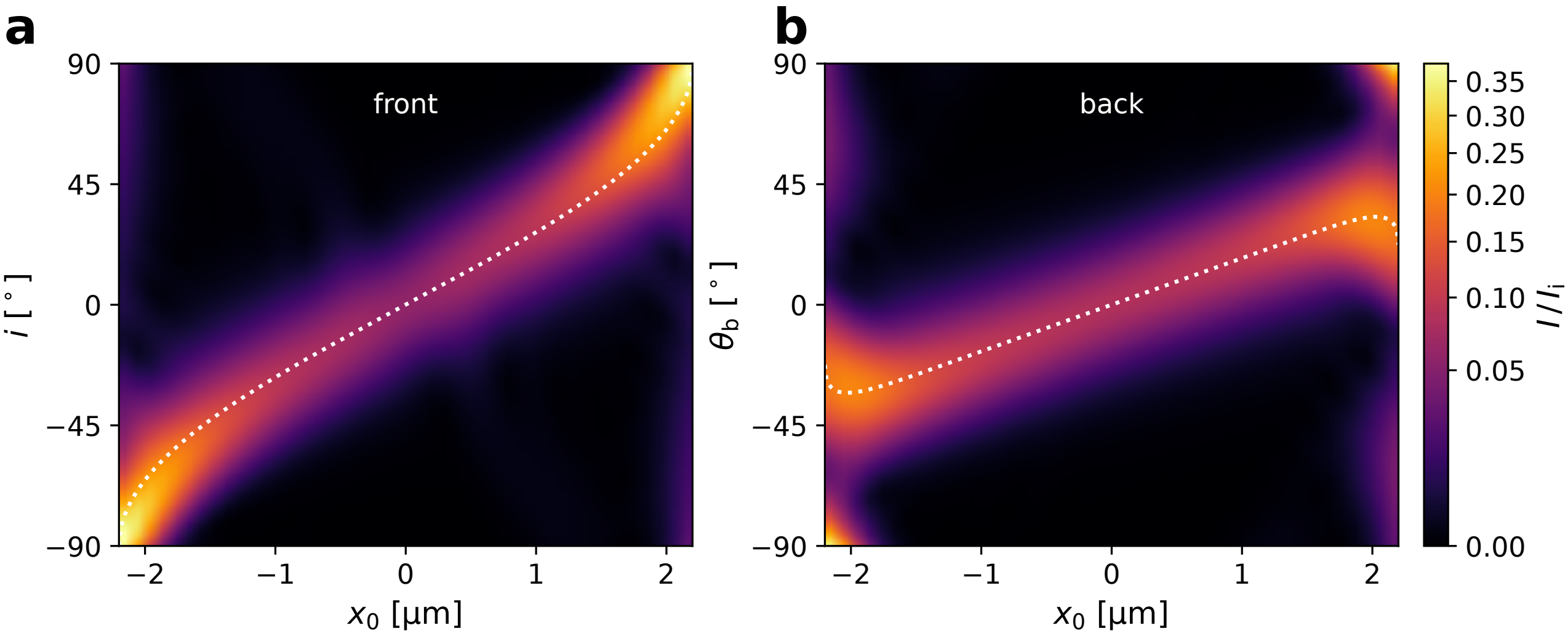

Fig. S4 Wave-optical analysis of the illumination transfer. Generalized Lorenz–Mie theory (GLMT) calculation for a focused Gaussian beam (waist $w_0 = 300\,\text{nm}$ located in the plane of the particle center, $\lambda = 532\,\text{nm}$, linear polarization) propagating along $-z$ and displaced laterally by x_0 along the x-axis from the particle center. **a, b** Surface intensity on the illuminated (front) and shadowed (back) hemisphere as a function of the beam offset x_0 and the position on the surface, given on the front face by the chief ray's angle of incidence i (the polar angle from the front pole, $\sin i = x_0/a$ at the intensity maximum) and on the back face by the polar angle θ_b from the back pole, both measured within the x–z plane and counted positive towards $+x$. Dotted lines show the geometric intersection $i = \arcsin(x_0/a)$ (front) and the chief-ray prediction $\theta_\text{b}(x_0)$ of Eq. (S56) (back). The position of the back-face intensity maximum depends linearly on the beam offset (slope $19.0°/\mu\text{m}$; deviation from a straight line below $0.24°$ for $|x_0| \leq 1.5\,\mu\text{m}$), close to the paraxial chief-ray slope $(2/m-1)/a = 17.3°/\mu\text{m}$. Intensities are normalized to the peak intensity I_i of the incident focused beam at its focus in the absence of the particle.

performed a wave-optical calculation based on generalized Lorenz–Mie theory (GLMT) for a single focused Gaussian beam, the building block of our illumination patterns, and compare it with the ray-optics (chief-ray) prediction (Fig. S**4**).

In the ray picture, the optically denser particle acts as a ball lens: the transmitted rays are focused towards the rear pole, where they form a demagnified image of the front-face illumination, bounded by a fold caustic and a shadow zone towards the equator. The GLMT calculation quantifies this transfer for a beam propagating along $-z$ and displaced by x_0 along the x-axis. The z-axis through the particle center pierces the surface at the front pole (facing the beam) and the back pole, and positions on the two faces are described by the signed polar angle θ, measured from the respective pole within the x–z plane and counted positive towards $+x$ (Fig. S**4**a,b). On the illuminated face, the intensity maximum lies at the geometric intersection $\theta = \arcsin(x_0/a)$, which coincides with the chief ray's angle of incidence i with respect to the local surface normal, $\sin i = x_0/a$. On the back face, the maximum follows the chief ray, which refracts according to Snell's law and reaches the back surface at

$$\theta_{\rm b}(x_0) = 2\arcsin\left(\frac{\sin i}{m}\right) - i \approx \left(\frac{2}{m} - 1\right)\frac{x_0}{a} + \left(\frac{1}{3m^3} - \frac{1}{6}\right)\left(\frac{x_0}{a}\right)^3, \tag{S56}$$

where $m = n_{\rm p}/n_{\rm w} = 1.20$ is the relative refractive index. For this index contrast the cubic coefficient (0.025) is 27 times smaller than the linear one (0.66) and vanishes exactly at $m = 2^{1/3}$, so the particle maps the incident illumination onto its back face almost perfectly linearly. Since $\theta_{\rm b}$ carries the same sign as the entry position, the transmitted light forms an upright image of the front-face pattern, demagnified by $2/m - 1 \approx 0.66$ and confined within $|\theta_{\rm b}| \lesssim 33^\circ$ of the rear pole; the mapping becomes stationary at $x_0 \approx 0.92\,a$ (the fold caustic of the ray picture), beyond which the beam clips the particle rim. The full-wave slope lies about 10 % above the paraxial chief-ray value, reflecting the finite angular spread of the focused beam.

Two consequences follow for the heating pattern. First, because the mapping is linear and upright, the transmitted light preserves the sign and ordering of the imposed intensity gradient, so the low-order heating moments q_0, q_1 and q_2, which determine the temperature profile and the resulting flow field, are not scrambled by refraction. Second, the redistribution is spatially localized (caustic ring and shadow zone) and therefore contributes mainly to high-order Legendre modes of the heat source density. These modes are strongly damped in the temperature field by the prefactors $[l\kappa_{\rm in} + (l+1)\kappa_{\rm out}]^{-1}$ (cf. Eq. (S21)) and are further smoothed by heat diffusion along the particle surface and by the spatially distributed absorption of the gold nanoparticles.

Consistent with this, the measured illumination profiles (Fig. S**3**c,d), the liquid-crystal isotherms (Figs. S**5** and S**7**), and the measured hydrodynamic flow fields, which are well reproduced by simulations assuming the idealized temperature profiles, confirm that refraction does not significantly affect the realized swimming modes.

S3 Experimental measurements of surface temperatures

In order to get experimental insight into the surface temperature of the illuminated microswimmer, we conducted an experiment in which we immersed the microswimmer in a liquid crystal. Exploiting the phase transition of the liquid crystal at the critical temperature of $T_{\mathrm{PT}} = 308\,\mathrm{K}$, we obtain a reference for estimating the surface temperature in the experiment. When the microswimmer is illuminated by laser light, it will heat up and eventually exceed T_{PT} so that an isotropic phase around the heated particle forms. Due to the refractive index change between the nematic and the isotropic phase, the phase boundary can be observed with an optical microscope under dark-field illumination [2].

Figure S**5** shows a series of microscopy images of two heated shaker squirmers (Fig. S**5**a, $\beta = -\infty$ and Fig. S**5**c, $\beta = \infty$) and of a puller (Fig. S**5**e, $\beta = 1$). With increasing laser light power P, the phase boundary increases. Analyzing the shape of the phase boundary proves the asymmetric temperature profile applied on the surface of the swimmer through laser light illumination. Figure S**5**b,d,f shows the analytical derived two-dimensional temperature profile following Eqs. (S53) and (S54) in a logarithmic color scale for $\beta = \pm\infty, 1$. The experimentally measured white isotherms exhibit excellent agreement with the analytical profile providing evidence for the precise illumination control.

S3.1 Measurement principle

For a quantitative estimate of the surface temperature ΔT_{surf}, we established the following measurement principle. The temperature field outside the particle in the quadrupole approximation (cf. Eq. (S21)) equals to

$$T_{\mathrm{out}}(r,\theta) = T_0 + \frac{q_0 a^2}{\kappa_{\mathrm{out}} r} + \frac{q_1 a^3}{(\kappa_{\mathrm{in}} + 2\kappa_{\mathrm{out}})r^2}\cos\theta + \frac{q_2 a^4}{(2\kappa_{\mathrm{in}} + 3\kappa_{\mathrm{out}})r^3}\frac{1}{2}(3\cos^2\theta - 1)\,. \tag{S57}$$

The phase transition boundary occurs where $T_{\mathrm{out}}(r_{\mathrm{exp}}(\theta),\theta) = T_{\mathrm{PT}}$. This defines an angle-dependent radius $r_{\mathrm{exp}}(\theta)$ where the phase transition occurs:

$$T_{\mathrm{PT}} = T_0 + \frac{q_0 a^2}{\kappa_{\mathrm{out}} r_{\mathrm{exp}}(\theta)} + \frac{q_1 a^3}{(\kappa_{\mathrm{in}} + 2\kappa_{\mathrm{out}})r_{\mathrm{exp}}^2(\theta)}\cos\theta + \frac{q_2 a^4}{(2\kappa_{\mathrm{in}} + 3\kappa_{\mathrm{out}})r_{\mathrm{exp}}^3(\theta)}\frac{1}{2}(3\cos^2\theta - 1)\,. \tag{S58}$$

By measuring the shape of this phase transition boundary $r_{\mathrm{exp}}(\theta)$, we can extract information about the temperature field and validate the heat source distribution parameters q_0, q_1, and q_2.

We can rewrite Eq. (S58) to $T_{\mathrm{PT}} - T_0 = q_0 f_0(r_{\mathrm{exp}}(\theta),\theta) + q_1 f_1(r_{\mathrm{exp}}(\theta),\theta) + q_2 f_2(r_{\mathrm{exp}}(\theta),\theta)$ with the functions

$$f_0(r_{\mathrm{exp}}(\theta),\theta) = \frac{a^2}{\kappa_{\mathrm{out}}\, r_{\mathrm{exp}}(\theta)}\,, \tag{S59}$$

$$f_1(r_{\mathrm{exp}}(\theta),\theta) = \frac{a^3\cos\theta}{(\kappa_{\mathrm{in}} + 2\kappa_{\mathrm{out}})\, r_{\mathrm{exp}}(\theta)^2}\,, \tag{S60}$$

$$f_2(r_{\mathrm{exp}}(\theta),\theta) = \frac{a^4}{(2\kappa_{\mathrm{in}} + 3\kappa_{\mathrm{out}})\, r_{\mathrm{exp}}(\theta)^3}\frac{1}{2}\left(3\cos^2\theta - 1\right)\,. \tag{S61}$$

For each radius r_i and angle θ_i , we define a function $A(r_i,\theta_i)$ as

$$A(r_i,\theta_i) = \begin{bmatrix} f_0(r_i,\theta_i) \\ f_1(r_i,\theta_i) \\ f_2(r_i,\theta_i) \end{bmatrix}^{\mathrm{T}}\,. \tag{S62}$$

For obtaining the individual heating contributions q_0, q_1 and q_2 which make the radius deviate from the spherical shape, we thus have to solve the linear system

$$\mathbf{A}\mathbf{q} = \mathbf{b}\,, \tag{S63}$$

with

$$\mathbf{b} = \begin{bmatrix} T_{\mathrm{PT}} - T_0 \\ T_{\mathrm{PT}} - T_0 \\ \vdots \\ T_{\mathrm{PT}} - T_0 \end{bmatrix} \in \mathbb{R}^N \,, \tag{S64}$$

where $\mathbf{A} \in \mathbb{R}^{N\times 3}$ is the matrix with rows $A(r_i, \theta_i)$ and $\mathbf{q} \in \mathbb{R}^3$ contains the unknown coefficients (q_0, q_1, q_2). Since $N > 3$, the system is overdetermined and we solve it numerically:

$$\mathbf{q} = (\mathbf{A}^{\mathrm{T}}\mathbf{A})^{-1}\mathbf{A}^{\mathrm{T}}\mathbf{b} \,. \tag{S65}$$

This approach allows calculation of the monopole (q_0), the dipole (q_1) and quadrupole (q_2) contributions to the heating as a function of the applied laser power for a measured radius $r_{\mathrm{exp}}(\theta)$.

S3.2 Heating contributions and surface temperatures

Figure S**6** shows q_0, q_1 and q_2 as functions of laser power P, extracted from measurements of $r_{\mathrm{exp}}(\theta)$ for the pusher and the neutral squirmer that are presented in the main text and for a puller with $\beta = 1$ (cf. Fig. **7**b). All expansion coefficients increase linearly with laser power P. The dashed lines show fits to the from $q_l = mP$. Inserting the obtained values for q_0, q_1 and q_2 into Eq. (S21) provides the surface temperature for the different swimming modes shown in Fig. S**6**d for a power of $P = 4.35\,\mathrm{mW}$. While the pusher reaches the lowest surface temperatures, with ΔT_{surf} up to 7 K, the neutral squirmer and the puller attain similar higher temperatures, with ΔT_{surf} reaching up to $11 - 13\,\mathrm{K}$. Thermal conductivities of $\kappa_{\mathrm{in}} = 0.03\,\mathrm{W/mK}$ and $\kappa_{\mathrm{out}} = 0.15\,\mathrm{W/mK}$ were used.
Liquid crystal measurements of the positive and negative shaker are presented in Fig. S**7**d-g. The angular variation of $r_{\mathrm{exp}}(\theta)$ confirms the presence of an asymmetric temperature profile induced on the swimmer's surface by the laser illumination further validating our approach to creating controlled swimming modes.
In the experimental system, a nearby wall (cf. Fig. S**7**a) significantly alters the temperature distribution generated by the heated microswimmer [3]. To account for this, numerical simulations were carried out to establish a correction factor linking the temperature increments measured experimentally in 5CB to those expected for a heated particle adjacent to a boundary in water. From these simulation, the relation $\Delta T_{\mathrm{5CB}} = 2.31\,\Delta T_{\mathrm{surf}}$ was obtained with the correction factor of 2.31 representing the numerically derived correction [4].

S4 Particle Image Velocimetry

Particle image velocimetry (PIV) determines the fluid velocity field from the motion of tracer particles recorded in consecutive microscopy images. The method is based on cross-correlation analysis of the tracer intensity patterns, which allows extraction of the average particle displacement between successive frames and thereby the underlying local fluid velocity. PIV is applied to time-resolved microscopy recordings of tracer particles dispersed in the fluid. Since the swimmer was immobilized during the measurements, the generated flow field was assumed to be stationary in time. This enables ensemble averaging over many image pairs to improve the signal-to-noise ratio.
The hydrodynamic flow fields were extracted using a custom-written Python-based PIV routine. For the analysis, each image was subdivided into square interrogation windows of size $w \times w$ px. A window size of $w = 20$ px was used. Neighboring interrogation windows overlapped by $l = 10$ px in order to increase spatial resolution while retaining sufficient tracer statistics. The resulting spacing between adjacent windows was therefore

$$\Delta = w - l \,.$$

The local displacement of tracer particles between two consecutive frames was obtained from the cross-correlation of the corresponding interrogation windows. To remove offsets caused by homogeneous illumination, the mean intensity of each interrogation window was subtracted prior to the analysis,

$$f^*(x,y) = f(x,y) - \langle f \rangle$$
$$g^*(x,y) = g(x,y) - \langle g \rangle \,.$$

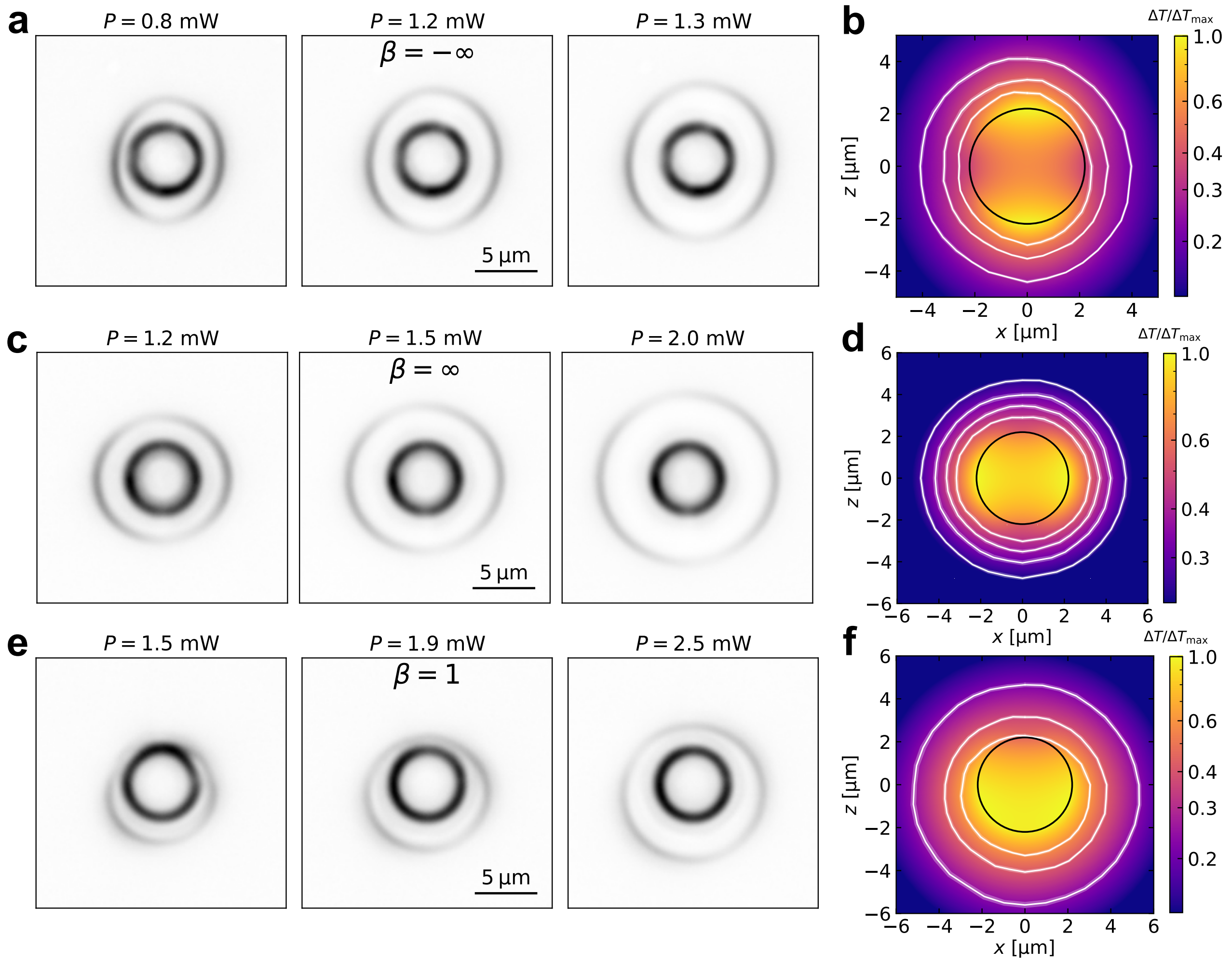

Fig. S5 Liquid crystal phase transitions for different squirmers. a, c, e The series of microscopy images shows the phase boundary of the liquid crystal which increases with increasing laser power P for all squirmer parameters ($\beta = \pm\infty, 1$). The phase boundary is asymmetric around the particle's center following the shape of the illumination profile of the laser light. **b, d, f** The analytical derived two-dimensional temperature field, displayed on a logarithmic color scale, is shown alongside the experimentally measured isotherms, which are indicated in white with shaded regions representing the associated uncertainties. The experimental temperature profile exhibits strong agreement with the theoretical prediction.

The cross-correlation function was then calculated using a Fast Fourier Transform (FFT)-based convolution,

$$C(x, y) = f^* \star g^* \,.$$

The resulting correlation matrix exhibits a peak at the most probable displacement $(x_{\text{peak}}, y_{\text{peak}})$ between the two interrogation windows. The displacement vector components were determined relative to the center position of the correlation matrix,

$$\Delta x = x_{\text{peak}} - x_{\text{cm}}$$
$$\Delta y = y_{\text{peak}} - y_{\text{cm}} \,.$$

Repeating this procedure for all interrogation windows yields the displacement field of the tracer particles between two consecutive frames. Dividing the displacement vectors by the frame interval $\tau = 33\,\text{ms}$ gives the local velocity components $u = \Delta x/\tau$ and $v = \Delta y/\tau$.

Because the swimmer was fixed in space, the induced flow field remained stationary throughout the experiment.

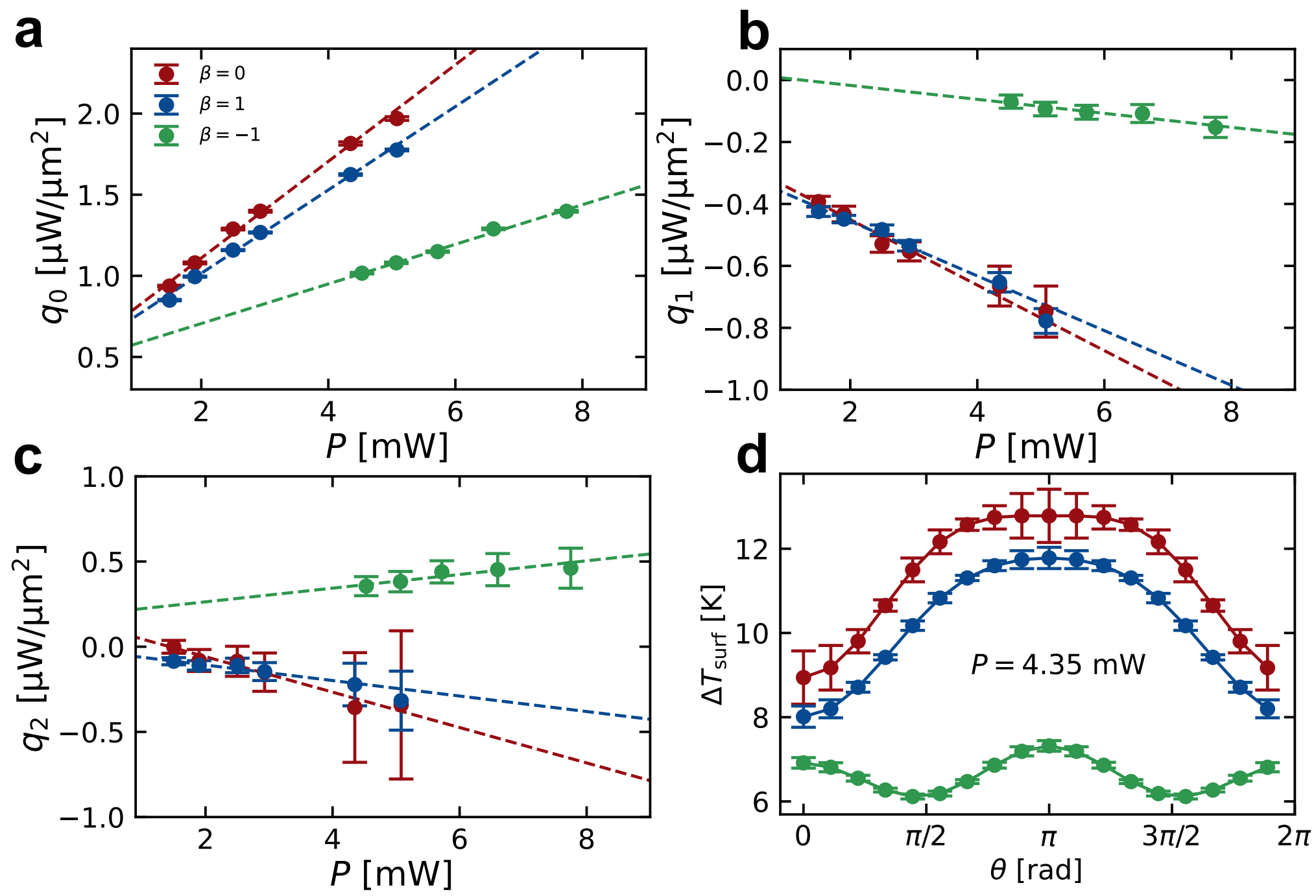

Fig. S6 Heating contributions of squirmers a,b,c Monopole (q_0), dipole (q_1) and quadrupole (q_2) heating contributions as functions of input laser power P for the neutral squirmer ($\beta = 0$), the pusher ($\beta = -1$), and the puller ($\beta = 1$) are extracted from measuring the phase transition radius $r_{\mathrm{exp}}(\theta)$ of an heated microswimmer in a liquid crystal. **d** Using the values for q_0, q_1 and q_2, the surface temperature ΔT_{surf} can be expressed as a function of θ. For the same laser power $P = 4.35\,\mathrm{mW}$, the pusher yields the lowest temperature around $7\,\mathrm{K}$, whereas the puller and neutral squirmer reach higher temperatures of approximately $11 - 13\,\mathrm{K}$.

Velocity fields extracted from multiple consecutive image pairs could therefore be averaged in time to suppress fluctuations arising from Brownian motion of the tracer particles. The averaged velocity field was obtained from

$$\bar{u}(x, y) = \langle u(x, y, t)\rangle_t$$
$$\bar{v}(x, y) = \langle v(x, y, t)\rangle_t\,.$$

The resulting mean velocity components $\bar{u}(x, y)$ and $\bar{v}(x, y)$ were subsequently used to reconstruct and visualize the streamlines of the hydrodynamic flow field.

S5 Simulation of hydrodynamic flow fields

Finite element simulations in COMSOL Multiphysics 6.1 were performed to validate the experimentally measured flow fields and to investigate the influence of confinement and boundary conditions on the hydrodynamic signatures of the swimmers. The heat transfer and laminar flow modules were used to simulate the temperature and flow fields for different squirmer modes.

The simulation domain consists of three cylinders, with two large glass cylinders defining its boundaries (diameter of 40 µm) and a central water domain containing a glass sphere with radius $a = 2.2\,\mathrm{\mu m}$, corresponding to the experimental particle size. Temperature profiles according to Eq. (S36) were imposed at the particle surface for different squirmer parameters. We apply slip boundary conditions with the dimensionless slip coefficient of $\sigma_{\mathrm{T}} = 0.001$ on all boundaries. The resulting velocity fields shown in the main text demonstrate the expected pusher, puller and neutral squirmer modes.

The confinement of the thin liquid film strongly influences the structure of the flow field. To investigate this effect systematically, simulations were performed for different confinement heights h. Figure S**8** shows the flow field of a neutral swimmer. For weak confinement and open boundary conditions ($h = 40\,\mathrm{\mu m}$), the flow field resembles the bulk Stokes flow around a squirmer (Fig. S**8**a,b). As the confinement is increased, hydrodynamic

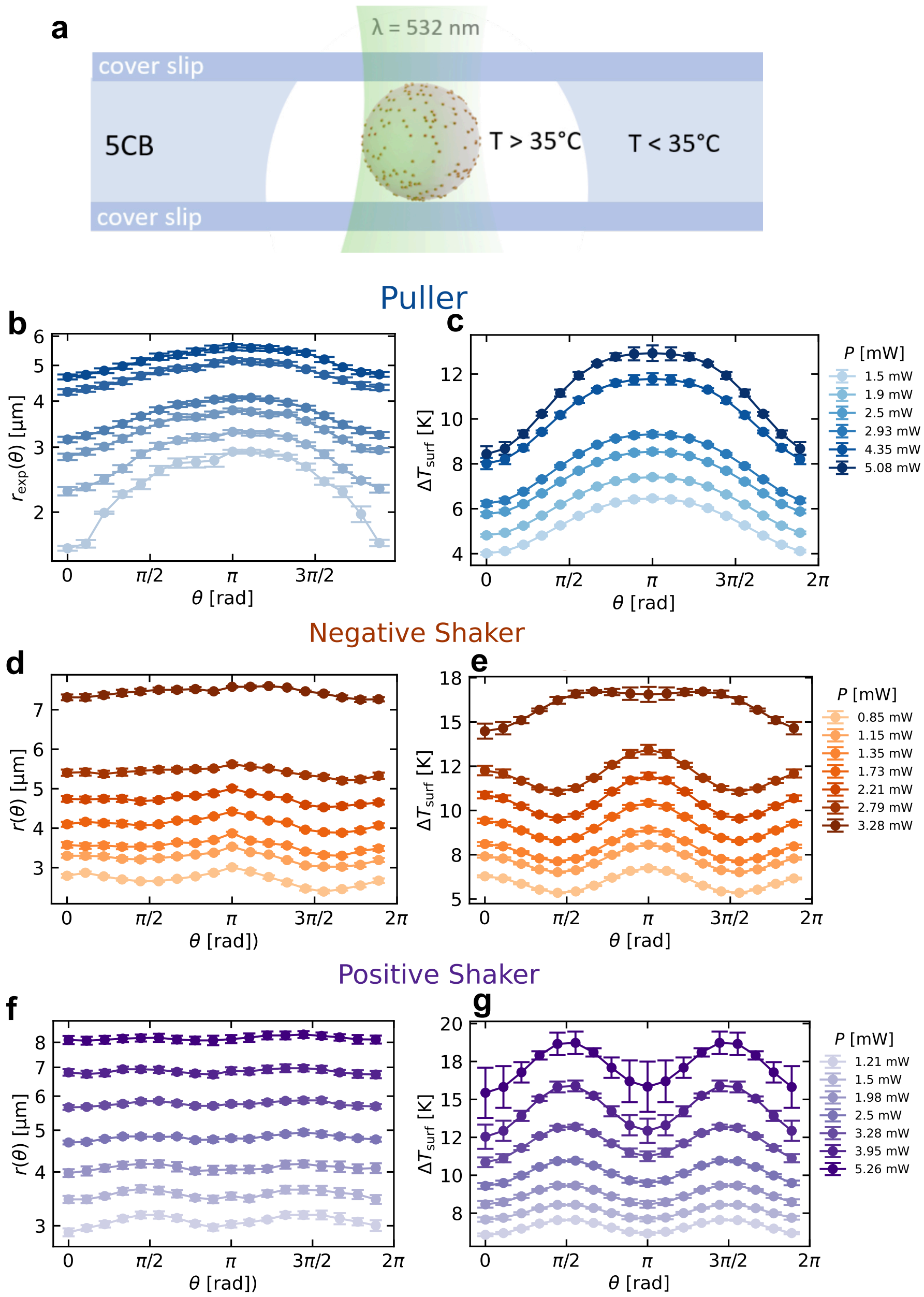

Fig. S7 Measurement of surface temperatures for different squirmers. **a** The microswimmer is immobilized at the microscope slide and immersed in a liquid crystal (5CB). **b,d,f** The angular variation of the measured phase transition radius $r_{\text{exp}}(\theta)$ reflects the asymmetric temperature fields, shown here for the puller and the negative and positive shaker, respectively. **c,e,g** The corresponding surface temperatures as a function of θ confirm the presence of an asymmetric temperature profile induced on the swimmer's surface by the laser illumination further validating our approach to creating controlled swimming modes.

interactions with the walls modify the flow structure as shown in Fig. S**8**c,d.

For the experimentally relevant confinement ($h \approx 5\,\mu\text{m}$), the flow field is substantially altered compared to the bulk case. The neutral swimmer presents a source dipole flow field with two characteristic vortices. When slip boundary conditions at the confining walls are introduced in the simulations, the vertical z-position of the vortices is shifted, further illustrating the sensitivity of the flow field to boundary conditions (see Fig. S**8**d,e).

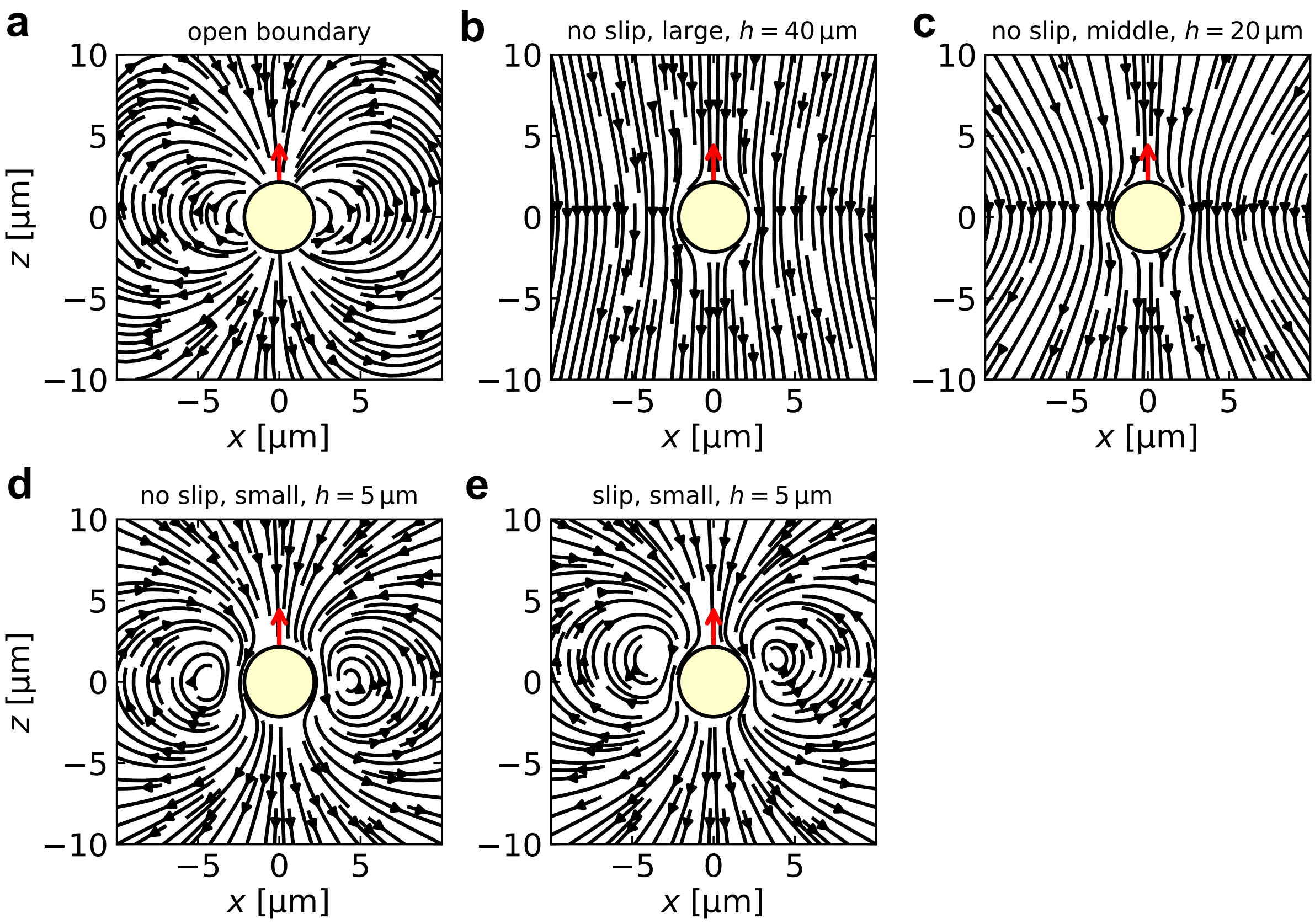

Fig. S8 Simulation of flow fields. **a** Flow field of a neutral squirmer under open boundary conditions. **b-d** With increasing confinement ($h = 40 - 5\,\mu$m), the structure of the flow field changes significantly due to hydrodynamic interactions with the confining walls. **d** In the strongly confined regime, the neutral swimmer exhibits a source dipole like flow field with two characteristic vortices. **e** Introducing slip boundary conditions at the walls shifts the vortices along the z-direction, resulting in a finite x-component of the velocity field at $z = 0$. All flow fields are shown in the laboratory frame of reference.

S6 Swimming efficiency

In the classical definition introduced by Lighthill [1], the efficiency is the ratio between the power required to tow a particle at speed U through a viscous fluid, $P_{\rm out} = 6\pi\eta a U^2$, and the power dissipated by the self-propelled motion. Within the classical squirmer framework, this input power is assumed to be entirely converted into viscous dissipation in the surrounding bulk fluid and can be expressed as $P_{\rm in} \propto \int_V 2\eta \mathbf{E} : \nabla \mathbf{u} dV$ where $\mathbf{E}$ is the strain rate tensor. Applying this definition to the squirmer model yields the velocity-invariant efficiency expression

$$\epsilon_{\rm L} = \frac{P_{out}}{P_{in}} = \frac{F_{\rm stokes} U}{P_{\rm in}} = \frac{1}{2+\beta^2}\,.$$

However, this classical approach neglects additional dissipation mechanisms that arise in phoretic swimmers, particularly within the interfacial boundary layer responsible for slip generation. For phoretic swimmers, the viscous dissipation can be separated into contributions from an outer region ($r > l_{\rm B}$) and an inner interfacial boundary layer ($r < l_{\rm B}$, where $l_{\rm B}$ denotes the boundary layer thickness [5]. Since $l_{\rm B} << a$, the no-slip condition at the particle surface generates very large velocity gradients within the boundary layer, leading to substantial viscous dissipation. It was shown that the dissipation associated with this inner region dominates over the bulk contribution and scales as $a^2 U^2 / l_{\rm B}$ [5].
Moreover, the ongoing conceptual discussion on the thermodynamic efficiency of active thermal machines highlights that coarse-grained efficiency definitions often neglect significant contributions to the total dissipation, and that a proper assessment of efficiency requires detailed knowledge of the underlying propulsion mechanism

[6, 7, 5]. Motivated by these considerations, we exploit the fact that for the thermophoretic particles used in our experiments, the energy influx into the system can be quantified directly. Here, we have thermo-phoretic swimmers with small gold nanoparticles attached to the surface. By illuminating the particle, each nanoparticle creates a temperature gradient on the surface and contributes to the dissipated power. Hence, we define efficiency as

$$\epsilon = \frac{P_{out}}{P_{in}} = \frac{6\pi\eta a U^2}{\int\int_{\text{sphere}} q(\theta) dS}\,, \tag{S66}$$

with $q(\theta)$ being the heat source density over the sphere's surface dS, responsible for generating the slip and thus the activity. The chosen input power P_{in} can be interpreted as an effective energetic cost function that includes all contributions associated with propulsion. For phoretic swimmers, this includes not only viscous dissipation in the bulk fluid but also the energy required to drive surface processes—such as heat generation, solute production, or ion exchange—that sustain the slip velocity.
The input power can be evaluated as

$$P_{\text{in}} = \int\int_{\text{sphere}} q(\theta) dS = 2\pi a^2 \int_0^\pi q(\theta)\sin\theta d\theta\,. \tag{S67}$$

Since $\int_0^\pi P_n(\cos(\theta))\sin\theta d\theta = 2\delta_{0n}$, we obtain from the formal expansion (S10) of the heat flux density

$$P_{\text{in}} = 4\pi a^2 q_0\,. \tag{S68}$$

This expression is valid for arbitrary axially symmetric heating profiles. It yields the swimming efficiency

$$\epsilon = \frac{6\pi\eta a U^2}{4\pi a^2 q_0} = \frac{2\eta(\chi/T_0)^2}{3a(\kappa_{\text{in}} + 2\kappa_{\text{out}})}\frac{q_1^2}{q_0}\,. \tag{S69}$$

S6.1 Constraints to swimming efficiency in the quadrupole approximation

The input power depends entirely on the monopole term q_0, which is determined by the positivity constraint: In the **linear regime** ($q_2 < -q_1/3$), the monopole term is constraint to $q_0 = -q_1 - q_2$ which leads to the input power

$$P_{\text{in,lin}} = 4\pi a^2(-q_1 - q_2) \;\text{ for } q_2 < \frac{-q_1}{3}\,. \tag{S70}$$

In the **non-linear regime** ($q_2 > -q_1/3$), the monopole term is constraint to $q_0 = \frac{q_2}{2} + \frac{q_1^2}{6q_2}$ which leads to the input power

$$P_{\text{in,non-lin}} = 2\pi a^2\left(\frac{q_1^2}{3q_2} + q_2\right) \;\text{ for } q_2 > \frac{-q_1}{3}\,. \tag{S71}$$

Based on the above expressions for the input power and on the swimming speed U from Eq. (S38), the corresponding efficiencies for the two regimes are obtained as

$$\epsilon_{\text{lin}} = \frac{6\pi\eta a\left(\frac{2}{3}\frac{\chi}{T_0}\frac{q_1}{\kappa_{\text{in}}+2\kappa_{\text{out}}}\right)^2}{4\pi a^2(-q_1 - q_2)} = \frac{2\eta\chi^2}{3aT_0^2(\kappa_{\text{in}} + 2\kappa_{\text{out}})^2}\frac{q_1^2}{(-q_1 - q_2)}\,, \tag{S72}$$

$$\epsilon_{\text{non-lin}} = \frac{6\pi\eta a\left(\frac{2}{3}\frac{\chi}{T_0}\frac{q_1}{\kappa_{\text{in}}+2\kappa_{\text{out}}}\right)^2}{2\pi a^2(\frac{q_1^2}{3q_2} + q_2)} = \frac{4\eta\chi^2}{3aT_0^2(\kappa_{\text{in}} + 2\kappa_{\text{out}})^2}\frac{q_1^2}{(\frac{q_1^2}{3q_2} + q_2)}\,. \tag{S73}$$

The expressions indicate that the efficiency depends solely on the dipole (q_1) and quadrupole (q_2) heating contributions. As described in Sec. S3, these quantities can be extracted from the temperature measurements using the phase transition of a liquid crystal.

S6.2 Maximum swimming efficiency in the quadrupole approximation

Figure 4d in the main paper shows the input power in both regimes for a fixed dipole heating value $q_1 = -0.4\,\mu\mathrm{W}\,\mu\mathrm{m}^{-2}$ as a function of the squirmer parameter β. The value of q_1 fixes the swimming speed through Eq. (S38). Let us now maximize the efficiency for a given swimming speed $U \propto -q_1 > 0$, and thus a given output power $P_{\mathrm{out}} = 6\pi\eta a U^2$, in the quadrupole approximation. For a speed fixed U, the efficiency (S69) is maximized by minimizing the overall input power and thus q_0.

In the linear regime ($q_2 < -q_1/3$), $q_0 = -q_1 - q_2$ is for a fixed $q_1 < 0$ maximized by taking largest possible $q_2 = -q_1/3$. This gives the maximum efficency and the corresponding squirmer parameter

$$\epsilon^{\star}_{\mathrm{lin}} = \frac{9}{4}\frac{\eta\chi}{aT_0}\frac{U}{\kappa_{\mathrm{in}} + 2\kappa_{\mathrm{out}}}\,, \tag{S74}$$

$$\beta^{\star}_{\mathrm{lin}} = -\frac{\kappa_{\mathrm{in}} + 2\kappa_{\mathrm{out}}}{2\kappa_{\mathrm{in}} + 3\kappa_{\mathrm{out}}}\,. \tag{S75}$$

In the non-linear regime ($q_2 > -q_1/3$), the minimum input power can be determined by solving $\partial P_{\mathrm{in,non-lin}}/\partial q_2 = \frac{-q_1^2}{3q_2^2} + 1 = 0$ which yields the condition $q_1^2 = 3q_2^2$. Under the conditions $q_1 < 0$ and $q_2 > 0$, this implies that $P_{\mathrm{in,non-lin}}$ reaches a global minimum for $q_2 = -q_1/\sqrt{3}$. The corresponding maximum and squirmer parameter are

$$\epsilon^{\star}_{\mathrm{non-lin}} = \frac{3\sqrt{3}}{2}\frac{\eta\chi}{aT_0}\frac{U}{\kappa_{\mathrm{in}} + 2\kappa_{\mathrm{out}}} = \frac{2}{\sqrt{3}}\epsilon^{\star}_{\mathrm{lin}}\,, \tag{S76}$$

$$\beta^{\star}_{\mathrm{non-lin}} = -\sqrt{3}\frac{\kappa_{\mathrm{in}} + 2\kappa_{\mathrm{out}}}{2\kappa_{\mathrm{in}} + 3\kappa_{\mathrm{out}}} = \sqrt{3}\beta^{\star}_{\mathrm{lin}}\,. \tag{S77}$$

In the quadrupole approximation, the most efficient non-linear swimmer is thus more efficient than the most efficient linear swimmer. Both optimal swimmers are pushers ($\beta < 0$). This is consistent with the temperature measurements (cf. Fig. S**6**d), which show that the pusher exhibits the lowest surface temperature. For typical values of $\kappa_{\mathrm{in}} = 0.03\,\mathrm{W}\,(\mathrm{m\,K})^{-1}$ and $\kappa_{\mathrm{out}} = 0.15\,\mathrm{W}\,(\mathrm{m\,K})^{-1}$, we find $\beta^{\star}_{\mathrm{lin}} = -0.64$ and $\beta^{\star}_{\mathrm{non-lin}} = -1.12$.

S6.3 Optimal heating and maximum efficiency beyond the quadrupole approximation

Let us now find the optimal heating pattern for a given propulsion speed $U \sim -q_1 > 0$ without relying on the quadrupole approximation. Given the axial symmetry of illumination profiles, an arbitrary heat flux density can be constructed from thin circular illumination profiles centered on the propulsion axis. Such thin circular profiles correspond to heat flux density $q_i(\theta)$ given by a delta function $q_i(\theta) = A_i\delta(\cos\theta - \cos\theta_i)$, with θ_i measuring the distance of the circle from the pole of the particle in the propulsion direction, and $A_i \geq 0$ measuring the intensity of the given circular profile. Any axially symmetric heating profile can then be written as

$$q(\theta) = \sum_{i=1}^{N_c} q_i(\theta) = \sum_{i=1}^{N_c} A_i\delta(\cos\theta - \cos\theta_i), \tag{S78}$$

where N_c is the (potentially infinite) number of required circular profiles.

The general heat source expansion is

$$q(\theta) = \sum_{l=0}^{\infty} q_l P_l(\cos\theta)\,. \tag{S79}$$

Using the orthogonality of the Legendre polynomials ($\int_{-1}^{1} P_l(x)P_m(x)dx = \frac{2}{2l+1}\delta_{lm}$) with $x = \cos\theta$ so that $dx = -\sin\theta d\theta$ ($\theta \in [0,\pi]$ and $x \in [-1,1]$), we obtain the expansion coefficients

$$q_l = \frac{2l+1}{2}\int_0^{\pi} q(\theta)P_l(\cos\theta)\sin\theta d\theta\,. \tag{S80}$$

For a thin circular profile $q_i(\theta) = A_i\delta(\cos\theta - \cos\theta_i)$ at arbitrary angle θ_i, the Legendre coefficients are then

$$q_{i;l} = \frac{2l+1}{2} A_i P_l(\cos\theta_i) \tag{S81}$$

and, due to linearity, the Legendre coefficients of the arbitrary axially symmetric profile (S78) are given by

$$q_l = \frac{2l+1}{2} \sum_{i=1}^{N_c} A_i P_l(\cos\theta_i). \tag{S82}$$

As explained at the beginning of this section, the swimming speed $U \sim q_1$, the input power $P_{in} \sim q_0$, and the swimming efficiency $\epsilon \sim U^2/P_{in} \sim q_1^2/q_0$. The Legendre coefficients involved are explicitly given by

$$q_0 = \frac{1}{2}\sum_{i=1}^{N_c} A_i, \tag{S83}$$

$$q_1 = \frac{3}{2}\sum_{i=1}^{N_c} A_i \cos\theta_i. \tag{S84}$$

S6.3.1 Maximum efficiency at fixed output power

Let us first consider the problem discussed above in the quadrupole approximation: the maximum efficiency at fixed speed, which is equivalent to minimum input power q_0 (or minimum dissipated power q_0/T) at fixed output power.
In this case, we need to minimize q_0 for a fixed q_1, with respect to locations and intensities of the individual thin circular profiles, θ_i and A_i. Since $A_i \geq 0$ and $|\cos\theta_i| \leq 1$, it holds

$$|q_1| = \frac{3}{2}\left|\sum_{i=1}^{N_c} A_i \cos\theta_i\right| \leq \frac{3}{2}\sum_{i=1}^{N_c} A_i = 3q_0, \tag{S85}$$

with equality if and only if all heating is localized at $\theta_i = 0$ or $\theta_i = \pi$, which are the optimal heating profiles. Nevertheless, let us now show how to obtain these results using the method of Lagrange multipliers, which can be readily adapted to other constrained optimization problems with a global optimum. Importantly, the present problem can be solved by this method only under the physical constraint $A_i \geq 0$, which guarantees convexity of the admissible set of coefficients A_i under a fixed linear constraint (either q_0 or q_1). Without the positivity constraint, the optimum generically lies on the boundary of the admissible set.
The Lagrange function corresponding to the problem at hand is given by

$$\mathcal{L} = q_0 + \lambda q_1 = \frac{1}{2}\sum_{i=1}^{N_c} A_i + \frac{3}{2}\lambda \sum_{i=1}^{N_c} A_i \cos\theta_i, \tag{S86}$$

where λ is a single Lagrange multiplier corresponding to the condition that q_1 is fixed.
Taking derivatives with respect to θ_i and A_i yields the optimality conditions

$$\frac{\partial\mathcal{L}}{\partial\theta_i} = -\frac{3}{2}\lambda A_i \sin\theta_i = 0, \tag{S87}$$

$$\frac{\partial\mathcal{L}}{\partial A_i} = \frac{1}{2} + \frac{3}{2}\lambda\cos\theta_i = 0. \tag{S88}$$

The first one is fulfilled for $\theta_i = 0$ and $\theta_i = \pi$. The second one requires that all θ_i corresponding to nonzero A_i are identical.
Assuming $q_1 < 0$, corresponding to $U > 0$ as in previous sections, we thus have $\theta_i = \pi$ for all $i = 1, \ldots, N_c$. The condition for a fixed q_1 using the optimal illumination profile gives

$$q_1 = -\frac{3}{2}\sum_{i=1}^{N_c} A_i = -3q_0^*, \tag{S89}$$

where

$$q_0^* = -\frac{q_1}{3} \tag{S90}$$

is the optimal average density of input heating power.

In accord with our conclusion from the equality (S85), the optimal illumination profile that yields maximum efficiency (i.e., minimum input power or dissipation) at fixed output power (swimming speed) is a focused profile that delivers all heating power to the pole of the particle opposite the swimming direction. This is intuitive, as such a profile produces the maximum asymmetry of the temperature field for a given energy input.

The corresponding maximum swimming efficiency and squirmer parameter are

$$\epsilon_\infty^\star = \frac{9}{2}\frac{\eta\chi}{aT_0}\frac{U}{\kappa_{\rm in} + 2\kappa_{\rm out}} = 2\epsilon_{\rm lin}^\star\,, \tag{S91}$$

$$\beta_\infty^\star = -5\frac{\kappa_{\rm in} + 2\kappa_{\rm out}}{2\kappa_{\rm in} + 3\kappa_{\rm out}} = 5\beta_{\rm lin}^\star\,. \tag{S92}$$

The most efficient swimmer is therefore more efficient than the best linear swimmer in the quadrupole approximation by a factor of 2, and than the best non-linear swimmer in the quadrupole approximation by a factor of $\sqrt{3} \approx 1.73$. Its squirmer parameter is substantially more negative than that of both swimmers in the quadrupole approximation—by a factor of five compared to the linear case and by a factor of three compared to the nonlinear case—indicating that it is a markedly stronger pusher.

S6.3.2 Maximum efficiency at fixed input power

The present derivation can be generalized to arbitrary constraints and utility functions. Let us demonstrate how to do it for a problem complementary to the one above. We will now assume that the input power and thus q_0 is fixed, and we will maximize the swimming speed (as well as output power and efficiency) and thus q_1 under this condition.

The corresponding Lagrange function is given by

$$\mathcal{L} = q_1 + \lambda q_0 = \frac{3}{2}\sum_{i=1}^{N_c} A_i\cos\theta_i + \frac{1}{2}\lambda\sum_{i=1}^{N_c} A_i, \tag{S93}$$

where λ is a single Lagrange multiplier corresponding to the condition that q_0 is fixed and $A_i > 0$.

Taking derivatives with respect to θ_i and A_i yields the optimality conditions

$$\frac{\partial\mathcal{L}}{\partial\theta_i} = -\frac{3}{2}A_i\sin\theta_i = 0, \tag{S94}$$

$$\frac{\partial\mathcal{L}}{\partial A_i} = \frac{3}{2}\cos\theta_i + \frac{1}{2}\lambda = 0. \tag{S95}$$

Similarly to above, the first equation implies $\theta_i = 0$ or $\theta_i = \pi$, and the second requires that all θ_i are equal. Assuming again a positive velocity and thus $q_1 < 0$, the optimal illumination profile is thus the same as in the previous case. The minimal value of q_1 corresponding to the maximum speed at fixed q_0 is

$$q_1^* = -\frac{3}{2}\sum_{i=1}^{N_c} A_i = -3q_0. \tag{S96}$$

The corresponding efficiency is again given by Eq. (S91) and the corresponding squirmer parameter is given by Eq. (S92).

S6.3.3 Optimality when a cooling is possible

Allowing for cooling means that, in the language of the decomposition of the illumination profile (S78), some of the coefficients A_i and thus even the input power $\sim q_0$ can be negative. In such a case, the efficiency (S69) is no longer well defined, but one can still consider the above optimization problem in the sense of maximum speed at fixed input power and minimum input power at fixed speed. As discussed in Sec. S6.3.1, the method of Lagrange multipliers cannot be directly applied to solve these constrained optimization problems when A_i

can have both signs. At the same time, one cannot directly use the inequality (S85), because it only holds for $A_i > 0$.

Instead, we now show that the problem of maximizing $|q_1|$ for fixed q_0 is not well posed when A_i can be both positive and negative. Indeed, denoting by A_+ and A_- the sums of the absolute values of all positive and negative coefficients A_i, respectively, we can write

$$q_0 = \frac{1}{2}(A_+ - A_-). \tag{S97}$$

Fixing q_0 hence fixes the difference between A_+ and A_-, but not their magnitudes. The swimming speed ($|q_1|$) is maximized by localizing all heating contributions at one pole of the particle (say $\theta_i = \pi$ for $A_i > 0$) and cooling at the opposite pole ($\theta_i = 0$ for $A_i < 0$). This yields the maximum value of $|q_1|$ over θ_i,

$$|q_1| = \frac{3}{2}(A_+ + A_-). \tag{S98}$$

If we are also allowed to maximize over the intensities A_i, we find that the maximum speed is in this case unbounded, because the constraint in Eq. (S97) can be satisfied even in the limit $A_\pm \to \infty$. This remains true even when $q_0 = 0$.

On the other hand, the problem of minimizing q_0 for a given $|q_1|$ is well posed even in this case. The solution is the same as above, but $A_\pm$ can no longer diverge due to the constraint in Eq. (S98). Hence, the optimal solution is to use only cooling and localize it at one pole of the particle, which yields the most negative input power.

Finally, let us discuss when the squirmer parameter $\beta \sim q_2/q_1$ for an optimal illumination profile vanishes, and thus when a neutral swimmer becomes optimal. From the expression for the Legendre coefficients in Eq. (S82), it follows that

$$q_2 = \frac{5}{2}\sum_{i=1}^{N_c} A_i P_2(\cos\theta_i). \tag{S99}$$

As shown above, optimality requires $\theta_i = \pi$ for $A_i > 0$ and $\theta_i = 0$ for $A_i < 0$. Because even Legendre polynomials satisfy $P_{2n}(x) = P_{2n}(-x)$, this yields

$$q_2 = \frac{5}{2}P_2(\cos\theta_0)\sum_{i}^{N_c} A_i = 5P_2(\cos\theta_0)\, q_0, \tag{S100}$$

and we see that neutral swimmers are optimal only when q_0 vanishes. In this case, not only q_2 but also all higher even coefficients q_{2n} in the expansion vanish.

Supplementary References

[1] Lighthill, M. J. On the squirming motion of nearly spherical deformable bodies through liquids at very small reynolds numbers. *Communications on pure and applied mathematics* **5**, 109–118 (1952).

[2] Horn, R. G. Refractive indices and order parameters of two liquid crystals. *Journal de physique* **39**, 105–109 (1978).

[3] Baffou, G., Quidant, R. & Girard, C. Thermoplasmonics modeling: A green's function approach. *Physical Review B—Condensed Matter and Materials Physics* **82**, 165424 (2010).

[4] Rohde, L., Quinn, D., Paul, D. & Cichos, F. Regulated polarization of active particles in local osmotic flow fields. *Communications Physics* **8**, 213 (2025).

[5] Sabass, B. & Seifert, U. Efficiency of surface-driven motion: Nanoswimmers beat microswimmers. *Physical Review Letters* **105**, 218103 (2010).

[6] Wiese, R., Kroy, K. & Holubec, V. Modeling the efficiency and effective temperature of bacterial heat engines. *Physical Review E* **110**, 064608 (2024).

[7] Bebon, R., Robinson, J. F. & Speck, T. Thermodynamics of active matter: Tracking dissipation across scales. *Physical Review X* **15**, 021050 (2025).
