# Supplementary material for: Programmable hydrodynamics of active particles
Source: Nat Commun. 2026 Sep 2;17:9406. doi: 10.1038/s41467-026-77281-x (PMC13538562; doi:10.1038/s41467-026-77281-x)
Supplement: Supplementary file 2 [file 41467_2026_77281_MOESM2_ESM.pdf]

Description of Additional Supplementary Files

File Name: Supplementary Movie 1

Description: Supplementary Movie 1 shows the trajectory of an active microswimmer with real-time reconfiguration. During continuous motion, the swimmer instantaneously switches between distinct, color-coded swimming modes. We track the swimmer's position in each frame and dynamically reposition and reorient the illumination pattern, enabling us to realize an arbitrary, user-defined trajectory. The scale bar represents 5 μm.

File Name: Supplementary Movie 2

Description: Supplementary Movie 2 shows the trajectory of an active microswimmer with real-time reconfiguration. During continuous motion, the swimmer instantaneously switches between distinct, color-coded swimming modes. We track the swimmer's position in each frame and dynamically reposition and reorient the illumination pattern, enabling us to realize an arbitrary, user-defined trajectory. The scale bar represents 5 μm.
