# Supplementary material for: Programmable hydrodynamics of active particles
Source: Nat Commun. 2026 Sep 2;17:9406. doi: 10.1038/s41467-026-77281-x (PMC13538562; doi:10.1038/s41467-026-77281-x)
Supplement: Supplementary file 3 [file 41467_2026_77281_MOESM5_ESM.pdf]

nature portfolio

Peer Review File

Programmable Hydrodynamics of Active Particles

Corresponding Author: Professor Frank Cichos

This file contains all reviewer reports in order by version, followed by all author rebuttals in order by version.

Version 0:

Reviewer comments:

Reviewer #1

(Remarks to the Author)
The paper is interesting for several reasons. First, it introduces an experimental technique where the thermo-osmotic propulsion of a particle can be arbitrarily controlled and therefore squirmers can be "programmed" as pushers, pullers, etc. This is already a major breakthrough that in principle allows novel experiments studying the interaction of different squirmers with walls or other swimmers. Second, it shows that the efficiency follows different rules if the positivity of the power input is taken into account (or, in general, any constraint or cost function). Although this is shown for thermally driven swimmers, I expect that the same idea will be applicable to other mechanisms, like autophoretic swimmers. The experiments are supported by numerical results and analytical theory. Overall, the paper is well written and in my opinion interesting for a broad experimental and theoretical community. I recommend it for publication in Nature Communications with the following minor comments:

1) The fact that the illumination pattern needs to follow each swimmer after determining its position can be a limitation when studying collective effects in dense swimmer suspensions, particularly in 3D. Is there a perspective of combining absorbers with different wavelength dependence and then modulating the pusher/puller/neutral nature just through the frequency spectrum of illumination?

2) Fig 3c,d,e and text: I do not understand the flow along the x-axis. First, it is not stated whether the flow is measured at z=0, but I will assume so. Second, using a separate normalization for the x and the z component can be misleading if one of them is much smaller than the other. We therefore do not see whether there is indeed "no variation in |β|" (p.8). But, most importantly, a neutral squirmer is expected to have zero velocity in x direction at z=0. The fact that there is some velocity could either show that the squirmer is not precisely neutral, or that some higher squirmer modes are present. This discrepancy is neither pointed out nor explained.

3) The definition of Lighthill's efficiency is somewhat imprecise. In the main text, it is introduced as "the ratio of mechanical output power to ...". For a force-free swimmer, there is no mechanical output power - all work is dissipated in the fluid. Rather, it is the power needed to pull the same body at the same speed by an external force. In SI, section S5, it is stated that "The output power involves the sum of all viscous losses causing the directed motion...", which is also not an accurate description of the term in the numerator.

4) The application of the Lighthill efficiency (Eq. 3) to the thermophoretic swimmer requires some care. The original idea behind this efficiency measure is that the dissipation is proportional to U^2, which makes the efficiency invariant of the velocity. For the swimmers reported here, velocity U is proportional to the power P (Fig. 4c). This means that the efficiency ε is no longer velocity-invariant. Minimizing the dissipation at a given speed or maximizing the speed at a given dissipation will not necessarily lead to the same outcome. As long as all experiments are carried out at the same swimming velocity there will certainly be no problem, but the limitations of the efficiency measure should be mentioned.

Reviewer #2

(Remarks to the Author)
Summary of the paper

The authors demonstrate a thermophoretic microswimmer whose surface heating pattern can be dynamically programmed to

tune its hydrodynamic flow field, and they use this control to study how physical constraints on propulsion affect energetic efficiency. They argue that the optimal swimming mode differs from the prediction of idealized hydrodynamic theory.
In particular, the authors discuss that both biological and synthetic microswimmers move through viscous fluids by pushing and pulling the surrounding liquid, thereby creating characteristic flow patterns. Both types of swimmers are subject to physical constraints that restrict the kinds of flow patterns they can produce. These constraints are not taken into account in hydrodynamic theory, which predicts that neutral swimmers are the most efficient. As the authors note, experiments to date have not been able to systematically test how changing flow type affects efficiency, because existing systems have "prescribed" flow types that cannot be changed once created.
To address this limitation, the authors extend an experimental system previously developed in their lab, consisting of spherical colloids decorated with gold nanoparticles, by adding dynamic control to the external mechanism that drives their propulsion. This allows them to switch between different flow types in real time. They develop a simple theory that predicts the heating pattern required to generate a specific flow field and use it to design the corresponding experimental intensity pattern. After showing that they can generate flow fields that closely match their theoretical predictions and measuring corresponding efficiencies, they find that pushers are more efficient than neutral swimmers. The authors compare this result with ideal hydrodynamic theory and argue that real-world constraints can alter what "optimal" behavior looks like.

General assessment

The authors build on previous work from the Cichos lab (e.g., Refs. 22, 36, 50), in which thermophoretic propulsion was generated by laser-induced heating within a feedback loop that tracked the particle and updated the heating position in real time. In that earlier system, controlling the position of the localized heat source determined the particle's direction of motion. In the present work, the authors introduce dynamic control over the illumination itself, enabling programmable and spatially structured heating patterns across the particle surface and, consequently, real-time control over the generated flow field. They also develop a simple theoretical framework to determine the heating patterns required to produce a desired flow field, and they show good agreement between theoretical and experimental flow fields.
The manuscript is well written and accessible even to non-specialists. The authors present a meaningful and useful extension of their experimental platform that should be of broad interest to the active matter community, particularly to researchers working on active colloids. A notable strength of the work is the ability to switch the hydrodynamic mode in real time for the same particle. This provides a cleaner and more convincing comparison than approaches based on different particle batches, where other properties such as shape, surface chemistry, and propulsion mechanism could vary simultaneously. The ability to switch and monitor modes in real time thus offers a compelling way to isolate the role of hydrodynamic mode itself.
That said, while I appreciate the technical advance and find the work methodologically strong, I have concerns about the interpretation and framing of the results, which in my view are somewhat overstated. In particular, I am not fully convinced by the claims regarding the generality of the findings, the comparison with ideal theory and the definition of the key metric used to define efficiency, as outlined in the major points below.
Thus, although in principle the results are interesting and, in my view, worthy of publication in Nature Communications, I would not recommend publication in the current form. Revisions are needed to clarify the scope and generality of the authors' claims, as well as their choice of efficiency metric and how it relates to the definitions used in the literature against which they compare their results.

Major points

1. Optimality/notion of optimality:
The authors repeatedly make a broad claim about the generality of their result that the "pusher mode is more optimal". For example, on page 3 they write: "We find a striking result: …. This demonstrates that physical constraints on control parameters fundamentally alter performance optima, shifting the optimal mode from neutral to pusher. Importantly, this principle extends beyond thermophoretic swimmers to any system where the propulsion mechanism imposes constraints on achievable surface flows." Based on the evidence presented, this statement appears overstated.
The manuscript's results concern a specific propulsion mechanism (thermophoresis) under the particular constraint of positive-only surface heating. However, "optimal swimming" is not a universal notion; it depends sensitively on imposed constraints, the environment, interactions, and even the chosen definition of efficiency (see below points). See also, e.g., the following references: [Zhu et al., Phys. Fluids (2012), where efficiency globally changes with the fluid, and neutral/puller swimmers remain competitive or better], [Klindt et al., PRE (2015), where biological swimmers do not minimize hydrodynamic dissipation, and efficiency is not the main design principle], [Bailey et al., PRE (2024)], and [Singh et al., Langmuir (2020), where performance is interaction-dependent]. For this reason, swimmer performance cannot be reduced to a single universal optimum, and there is no unique "best swimmer."
This context dependence seems important and is not adequately discussed in the manuscript. The authors should discuss this context-dependency of "optimality" (beyond the discussion already included on page 12 comparing spherical and non-spherical swimmers), and either substantiate their broader claim more carefully or revise it to reflect the more limited scope of their finding.

2. Comparison with hydrodynamic theory:
In addition, the authors repeatedly emphasize that hydrodynamic theory predicts an optimum at $\beta = 0$ (neutral swimmer), whereas their results identify a negative value of β (pusher swimmer) as optimal. While a comparison with hydrodynamic theory may in principle be useful, there appears to be a fundamental difference in how efficiency is defined. In the present work (cf. Eq. (3)), efficiency is defined by comparing the mechanical output to the power absorbed through particle heating (i.e., the total energy input from the propulsion mechanism; that is, efficiency here depends on how energy is supplied). The

conclusion that pushers are more efficient seems to reflect the specifics of the thermophoretic system with positive heating, rather than a universal statement about hydrodynamic optimality or a general hydrodynamic principle.
In particular, the efficiency in Eq. (3) contains the absorbed power (P_{abs}) in the denominator, rather than the viscous dissipation in the fluid used in the hydrodynamic theories cited by the authors. Thus, the efficiencies being compared appear to be fundamentally different. The authors should address this distinction explicitly and discuss how meaningful it is to compare the two optima directly, namely the hydrodynamic optimum at $\beta = 0$ and the negative-β optimum found here.
Indeed, a comparison with a treatment such as that in Ref. 44 seems more natural, since the definition of efficiency is more closely aligned with the one adopted by the authors. As the authors themselves note, Ref. 44 likewise finds that propulsion localized near the pole is the most efficient. In light of this agreement with a more directly comparable classical result, it is not clear how surprising the present finding actually is. Unless the authors can provide a compelling reason why comparison with the hydrodynamic models is more appropriate, the repeated characterization of their result as unexpected should be revised.

3. Further implications of the efficiency definition:
A further issue concerning the definition of efficiency itself, specifically the use of the power absorbed by the particle as the relevant input quantity: as the authors state, the absorbed power varies considerably between modes. Because the efficiency metric combines hydrodynamic drag with mode-dependent energy absorption, the conclusion that the pusher mode is optimal becomes somewhat ambiguous. Is the apparent optimality of pushers due to a more efficient flow field, or does it simply result from lower energy input requirements? In other words, pusher swimmers may appear more optimal not because of hydrodynamics, but because they absorb less energy.
Indeed, Figs. 4d and 4e seem to suggest that pusher swimmers are simply energetically cheaper, in the sense that they provide the most energy-efficient way to achieve a given propulsion speed, or equivalently, require the least energy input to reach the same speed.
As a separate note, this definition appears problematic in the limiting case. Consider a hypothetical particle that absorbs no power at all. Then even a purely Brownian particle with an arbitrarily small but nonzero velocity would formally have infinite efficiency, despite not extracting any usable work from absorbed energy. The authors should therefore justify their choice of efficiency definition more carefully and explain why this apparent pathology does not undermine their analysis.

Additional points

1. Unless I am mistaken, neutral swimmers can rarely be found in nature (microorganisms are not perfectly symmetric, have time dependent strokes, switch between modes). In addition, I am not aware of hydrodynamically neutral colloidal swimmers (artificial swimmers may achieve neutrality as a transient state using external fields). The authors only cite one theoretical article in the Introduction when they introduce the notion of “neutral” swimmer on page 2. Are the authors aware of any other examples of neutral swimmers (either biological or synthetic)? Maybe they would like to comment on that, especially in the context of comparing neutral-like flow in natural and laboratory settings to ideal theory.

2. The authors state in the Introduction that investigating the optimal swimming mode requires real-time control over the surface boundary conditions. The authors may want to explicitly clarify for their readers why real-time control is advantageous for their study.

3. The authors state in the concluding paragraph of the Introduction: “a perspective that opens new directions for exploring morphological intelligence and adaptive strategies in both biological and synthetic active systems.” Can the authors clarify A) what morphological intelligence in synthetic matter is, and B) how this work may aid in exploring that, perhaps via an example?

4. Regarding Fig. 3:
A. The case $\beta = -\infty$ appears to deviate much more strongly from the theoretical curve than the other cases shown, for which the agreement is considerably better. Could the authors comment on the origin of this qualitative discrepancy between experiment and theory? For example, on the experimental curve there does not appear to be a clear maximum at negative x values, in contrast to the theoretical prediction.
B. I found the curves of the simulation results difficult to distinguish in Fig. 3; could the authors improve their visibility, e.g., by increasing the line thickness?
C. In the Main text, it is stated that the “x-component remains symmetric about the x-axis with no variation in $|\beta|$ (Figs. 3c-e), while the z-component shows more complex behavior”. I am not sure I understand this statement: the x-component seems to vary significantly across different values of β, with the maximum being at negative x for negative β but at positive x for zero and positive β, so there clearly seems to be variation with β in both x- and z- component. Relatedly, what is the nature of the transition in the flow field as β is varied continuously from minus one to zero?

5. Since the functional versatility and adaptive behavior presented in this work is imposed/enabled through external, real-time control rather than intrinsic swimmer dynamics, I found different sentences as overstated. For example, on page 12: “This capability enables task optimization for specific environmental challenges …”, “The ability to toggle between these modes on demand facilitates adaptive navigation in complex environments.”, “Beyond efficiency optimization, the demonstrated real-time reconfigurability enables functional versatility: microswimmers can reversibly switch between acting as propellers, pumps, or stealth swimmers.”. It would be helpful if the authors refine these statements to make clear that the reported adaptability does not represent fully autonomous/intrinsic system behavior.

6. Experimental details:
A. The authors use particles decorated with nanoparticles, with approximately 10% surface coverage. How consistent is this

between particles in their batch? And can the authors comment on variations between particles and how this may affect their observed behavior?
B. In the Optical Microscopy part of the Methods section: “Particle image velocimetry (PIV) is used to extract velocities and to calculate streamlines.” The authors do not provide sufficient information on their analysis.
C. The size of the gold nanoparticles is mentioned at the end in the Methods section. The authors may consider adding this information when they first introduce the system on page 3 and/or in the legend of Fig. 1.

7. Data reporting:
A. Reporting Summary is missing. The readers would want to see information on statistics: e.g., how many particles were analyzed etc.
B. An example of insufficient reporting: Legends of Fig. 2 and Fig. 3 and Fig. 4 in the Main text: “.. from multiple measurements”. Can you clarify what multiple measurements means (e.g., from how many particles, or from how many measurements over the same particle)?
C. Will experimental data or codes be deposited in a public repository?

8. In light of the many mentions of “constraint” and “optimal”, a consideration of a more general constrained optimization problem would be interesting. While a full consideration of the general problem might be beyond the scope of this mainly experimental work, the authors could consider adding a brief discussion of how one would approach this problem in a more general theoretical setting and perhaps consider the simplest case (positive power and optimizing efficiency) in the Supplement.

9. Typos:
A. The sentence before Eq. S66 in the Supplement contains a couple of typos.
B. In the first sentence of Sec. S5.3, “relying to” should be “relying on”.

Reviewer #3

(Remarks to the Author)
In this manuscript, the authors develop an experimental approach that allows reversible on-demand switching between different swimming modes (pusher, puller, neutral mode) of thermophoretically-propelled particles. The authors subsequently use this technique to test theoretical predictions on swimming efficiency and find that their experiments contradict idealized hydrodynamic models. They allocate this finding to the requirement that the heat source density must be positive on the surface of the particles. While the work is interesting and has been executed well, its technical nature and most likely limited impact means that it will appeal mostly to a specialized audience. I therefore do not recommend publication in Nature Communications but in a more specialized journal.

Specific Points:

1. The central conclusion of the manuscript relies on a constraint that is specific to thermophoretic propulsion, namely, that the heat source density must remain positive everywhere ($q > 0$). Does this constraint-driven optimization translate to non-thermally driven systems, such as chemically powered colloids? In chemical systems where reactions can act as both sources and sinks (removing the $q>0$ constraint), can alternative physical limitations similarly shift the optimal swimming mode away from the idealized hydrodynamic predictions?
2. The authors note that they suspend swimmers in a thin liquid film. Looking at the methods, I noticed that this refers to a 5um film in which the 4.4um particles are suspended. What is the effect of this confinement on the fluid flow? (How) Do the results extend to thicker samples?
3. The authors report overall swimming efficiencies on the order of 10^{-15} and mention that the majority of the absorbed thermal energy is utilized to maintain the localized temperature gradients, rather than to generate the propulsion. Given this massive baseline energy cost, the authors should address whether the relative difference in absorbed power P_{abs} between the neutral and pusher modes is significant. A brief discussion whether this small difference is relevant to biological microswimmers or the design of synthetic swimmers would be helpful as well.
4. Can the authors elaborate why the assumption that the same illumination pattern is present on both sides of the particles holds? I would think that the incident laser illuminates the particle only on one hemisphere.
5. It might be helpful for the non-expert reader to include a brief explanation how the spatial coefficients of the incident light governs the propulsion force.
6. Does the laser which generates the heat affect the nanoparticles used as tracers for the flow?
7. Some more details on how the PIV was done (software package) would be nice to add.

Reviewer #4

(Remarks to the Author)
I co-reviewed this manuscript with one of the reviewers who provided the listed reports. This is part of the Nature Communications initiative to facilitate training in peer review and to provide appropriate recognition for Early Career Researchers who co-review manuscripts.

Reviewer #5

(Remarks to the Author)

I co-reviewed this manuscript with one of the reviewers who provided the listed reports. This is part of the Nature Communications initiative to facilitate training in peer review and to provide appropriate recognition for Early Career Researchers who co-review manuscripts.

Version 1:

Reviewer comments:

Reviewer #1

(Remarks to the Author)
The comments from my first report are all addressed in a satisfactory manner, I recommend the paper for publication in its current form.

Reviewer #2

(Remarks to the Author)
I co-reviewed this manuscript with one of the reviewers who provided the listed reports. This is part of the Nature Communications initiative to facilitate training in peer review and to provide appropriate recognition for Early Career Researchers who co-review manuscripts.

Reviewer #3

(Remarks to the Author)
I thank the authors for answering my questions and clarifying the points I raised in their reply. I particularly appreciate the new introduction in response to the question from another referee, which makes a clearer case for their work. I agree that in-situ adjustment of the hydrodynamics is novel and potentially exciting, but there are open questions as to whether this potential can be used, for example, at what distances can two particles be individually controlled. Despite many improvements, I still find the manuscript better suited for a more specialized journal.

In response to my earlier comments:

- R.3.1. I appreciate their reply and believe that it would be useful to integrate this clarification in the manuscript.

- R.3.3: As shown in the new simulations, the flow profile changes significantly in confinement, and I find this is not very clearly described in the text.

- R.3.4: Their reply on why they believe that the assumption that the illumination pattern is the same on the front and backside of the particle has as far as I can see not been integrated in the manuscript. I would recommend the authors to do this.

- I would also like to comment on their new outlook. I appreciate their openness that the swimmer's adaptability stems from external control. However, the statement "adaptability, sensing and decision making" can be confused with advanced sensing and adapting of the swimmer to the environment, when I think only the position of the particle is detected and the laser position adapted to it. Moreover, I don't see what the relation is with reference 48 which concerns a macroscopic robot that in no way resembles their work. I also don't understand how they will integrate such these abilities on the robot level. In short, I would tone down this paragraph.

Reviewer #4

(Remarks to the Author)
The revised version of the paper is substantially improved. I find it easier to follow, thanks to the definition of efficiency and range of validity of the results being clearer. Apart from the two minor comments below, I believe that this work is now of sufficient interest and quality to be published in Nature Communications.

Minor comments

1. In Fig. 3, I would suggest using a different color for the experimental results for $\beta = \pm\infty$ and the theoretical curves for $\beta = \pm 6$. Alternatively, the authors could include the theoretical curves for $\beta = \pm\infty$.

2. While I appreciate the added details on the statistical analysis, the information could be made more concrete, e.g., in the Methods section. For example, in the caption of Fig. 4, it is unclear what exactly is meant by "at least 3 different particles". Are the error bars in Fig. 4d larger for positive β than negative β because fewer particles were measured for these parameter values?

Reviewer #5

(Remarks to the Author)
I co-reviewed this manuscript with one of the reviewers who provided the listed reports. This is part of the Nature Communications initiative to facilitate training in peer review and to provide appropriate recognition for Early Career Researchers who co-review manuscripts.

Version 2:

Reviewer comments:

Reviewer #3

(Remarks to the Author)
The authors have addressed my requests satisfactorily.

Reviewer #4

(Remarks to the Author)
The authors have satisfactorily addressed all points, and I recommend the manuscript for publication.

Reviewer #5

(Remarks to the Author)
I co-reviewed this manuscript with one of the reviewers who provided the listed reports. This is part of the Nature Communications initiative to facilitate training in peer review and to provide appropriate recognition for Early Career Researchers who co-review manuscripts.

Open Access This Peer Review File is licensed under a Creative Commons Attribution 4.0 International License, which permits use, sharing, adaptation, distribution and reproduction in any medium or format, as long as you give appropriate credit to the original author(s) and the source, provide a link to the Creative Commons license, and indicate if changes were made.
In cases where reviewers are anonymous, credit should be given to 'Anonymous Referee' and the source.
The images or other third party material in this Peer Review File are included in the article's Creative Commons license, unless indicated otherwise in a credit line to the material. If material is not included in the article's Creative Commons license and your intended use is not permitted by statutory regulation or exceeds the permitted use, you will need to obtain permission directly from the copyright holder.
To view a copy of this license, visit https://creativecommons.org/licenses/by/4.0/

DETAILED RESPONSES TO REVIEWER COMMENTS

Reviewer 1

The paper is interesting for several reasons. First, it introduces an experimental technique where the thermo-osmotic propulsion of a particle can be arbitrarily controlled and therefore squirmers can be "programmed" as pushers, pullers, etc. This is already a major breakthrough that in principle allows novel experiments studying the interaction of different squirmers with walls or other swimmers. Second, it shows that the efficiency follows different rules if the positivity of the power input is taken into account (or, in general, any constraint or cost function). Although this is shown for thermally driven swimmers, I expect that the same idea will be applicable to other mechanisms, like autophoretic swimmers. The experiments are supported by numerical results and analytical theory. Overall, the paper is well written and in my opinion interesting for a broad experimental and theoretical community. I recommend it for publication in Nature Communications with the following minor comments:

Response: We thank the reviewer for a thorough review and perceptive questions, which have guided us in making important improvements to our manuscript.

R1.1 The fact that the illumination pattern needs to follow each swimmer after determining its position can be a limitation when studying collective effects in dense swimmer suspensions, particularly in 3D. Is there a perspective of combining absorbers with different wavelength dependence and then modulating the pusher/puller/neutral nature just through the frequency spectrum of illumination?

Response: We thank the reviewer for this suggestion. We agree that tracking individual swimmers and dynamically adapting the illumination pattern becomes increasingly challenging in dense suspensions and three-dimensional systems. However, extending experiments to 3D is feasible using holographic optical techniques, which enable dynamic light shaping and simultaneous control of multiple swimmers, though at the cost of significantly greater complexity as particle number and density grow.

For dense, three-dimensional suspensions, the laser fields of neighboring particles will overlap, leading to undesired perturbations of their temperature profiles and propulsion behavior.

Employing absorbers with different wavelength-dependent responses could indeed mitigate this issue. Swimmers functionalized with nanoparticles absorbing at distinct wavelengths would enable selective control of their propulsion mode through the illumination spectrum, thereby reducing cross-talk in multi-particle systems. Realizing this approach would require the design of microswimmers with tailored, wavelength-selective absorption properties, which is an interesting avenue for future work. We have added a corresponding sentence to the second-to-last paragraph of the discussion.

R1.2 I do not understand the flow along the x-axis. First, it is not stated whether the flow is measured at $z = 0$, but I will assume so. Second, using a separate normalization for the x and the z component can be misleading if one of them is much smaller than the other. We therefore do not see whether there is indeed "no variation in $|\beta|$" (p.8). But, most importantly, a neutral squirmer is expected to have zero velocity in x direction at $z = 0$. The fact that there is some velocity could either show that the squirmer is not precisely neutral, or that some higher squirmer modes are present. This discrepancy is neither pointed out nor explained.

Response: We thank the reviewer for pointing out this important aspect. First, we clarify that the flow fields shown in Fig. 3c–e are evaluated for the immobilized swimmers at $z = 0$, while Fig. 3f–h correspond to $x = 0$. This is now explicitly stated in the revised manuscript. We agree that using separate normalizations for the x- and z-components can obscure their relative magnitudes. Our intention was to emphasize that the decay of the x-component remains essentially unchanged across different values of $|\beta|$, whereas the z-component varies significantly with $|\beta|$. Figure 1 illustrates this by comparing experimental and simulated velocity components for different squirmer parameters: with increasing pusher or puller strength, the z-component changes noticeably, while the x-component remains nearly identical within each class. The manuscript has been revised accordingly.

Regarding the neutral squirmer, in an unbounded fluid it is indeed expected to exhibit zero x-velocity component at $z = 0$. In our system, however, the swimmer is confined within a thin film ($\approx 5\,\mu\text{m}$) and experiences boundary effects, including hydrodynamic interactions with the walls and thermo-osmotic slip at the glass surfaces. The latter shifts the vortex positions along z, giving rise to a finite x-component at $z = 0$. Figure 2 shows the simulated difference between slip and no-slip boundary conditions. We have added two sentences to the

main text discussing this point, and included additional simulations comparing slip, no-slip, and varying degrees of confinement in SI Section 5.

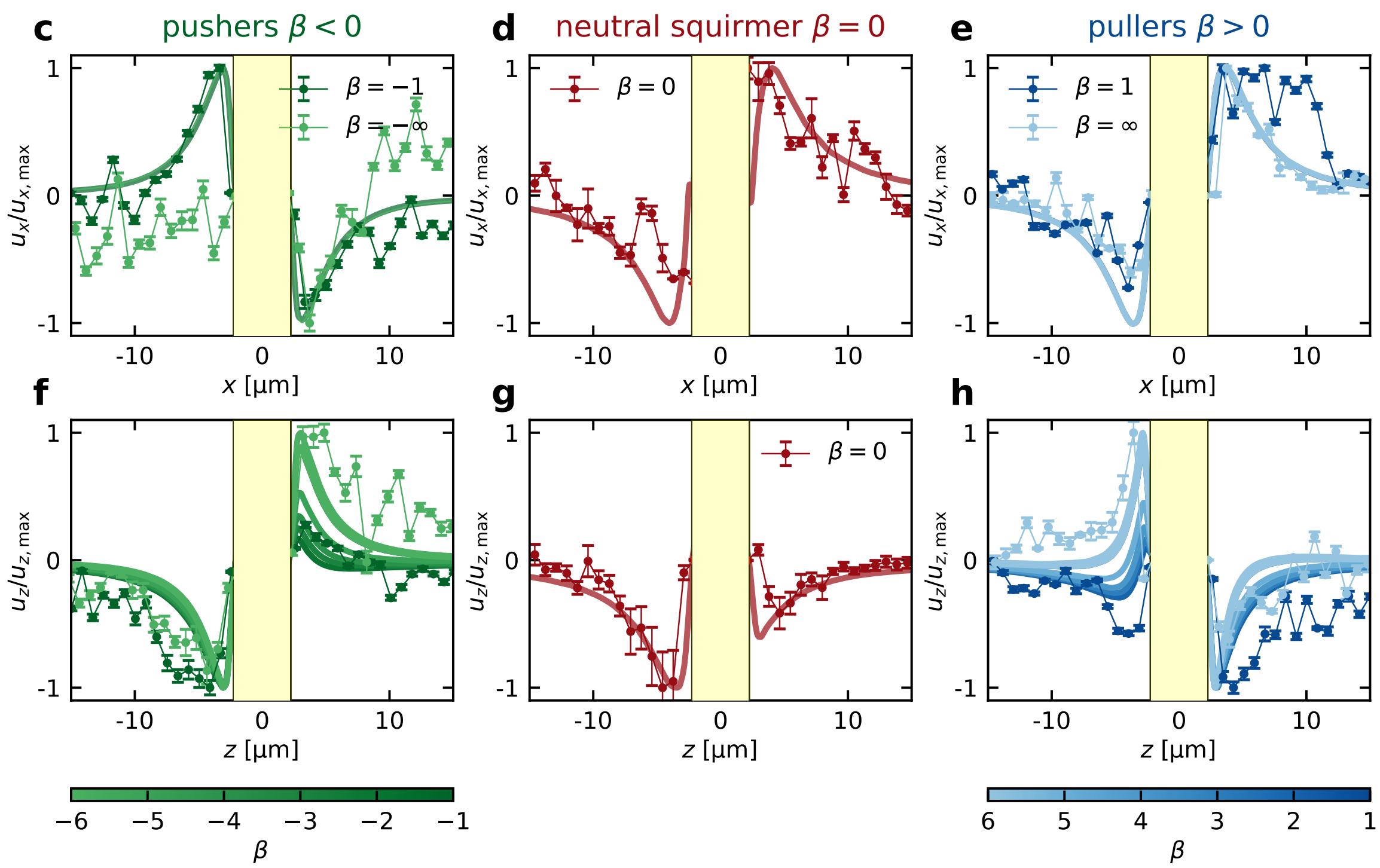

FIG. 1: Velocity components of the flow field along x at $z = 0$ and along z at $x = 0$.

R1.3 The definition of Lighthill's efficiency is somewhat imprecise. In the main text, it is introduced as "the ratio of mechanical output power to ...". For a force-free swimmer, there is no mechanical output power - all work is dissipated in the fluid. Rather, it is the power needed to pull the same body at the same speed by an external force. In SI, section S5, it is stated that "The output power involves the sum of all viscous losses causing the directed motion...", which is also not an accurate description of the term in the numerator.

Response: We thank the reviewer for pointing out this imprecision. The reviewer is correct: for a force-free swimmer there is no mechanical output power, and the numerator $6\pi\eta a U^2$ corresponds instead to the power that would be required to drag a rigid sphere of the same radius through the fluid at the same speed U by an external force. We have corrected the wording in both the main text and Supplementary Note S5 (now Supplementary Note S6) accordingly, and the revised definition now reads $\epsilon = 6\pi\eta a U^2/(4\pi a^2 q_0)$, with the numerator interpreted as the power required to drag a rigid sphere of the same radius at speed U rather than as a mechanical output of the swimmer itself.

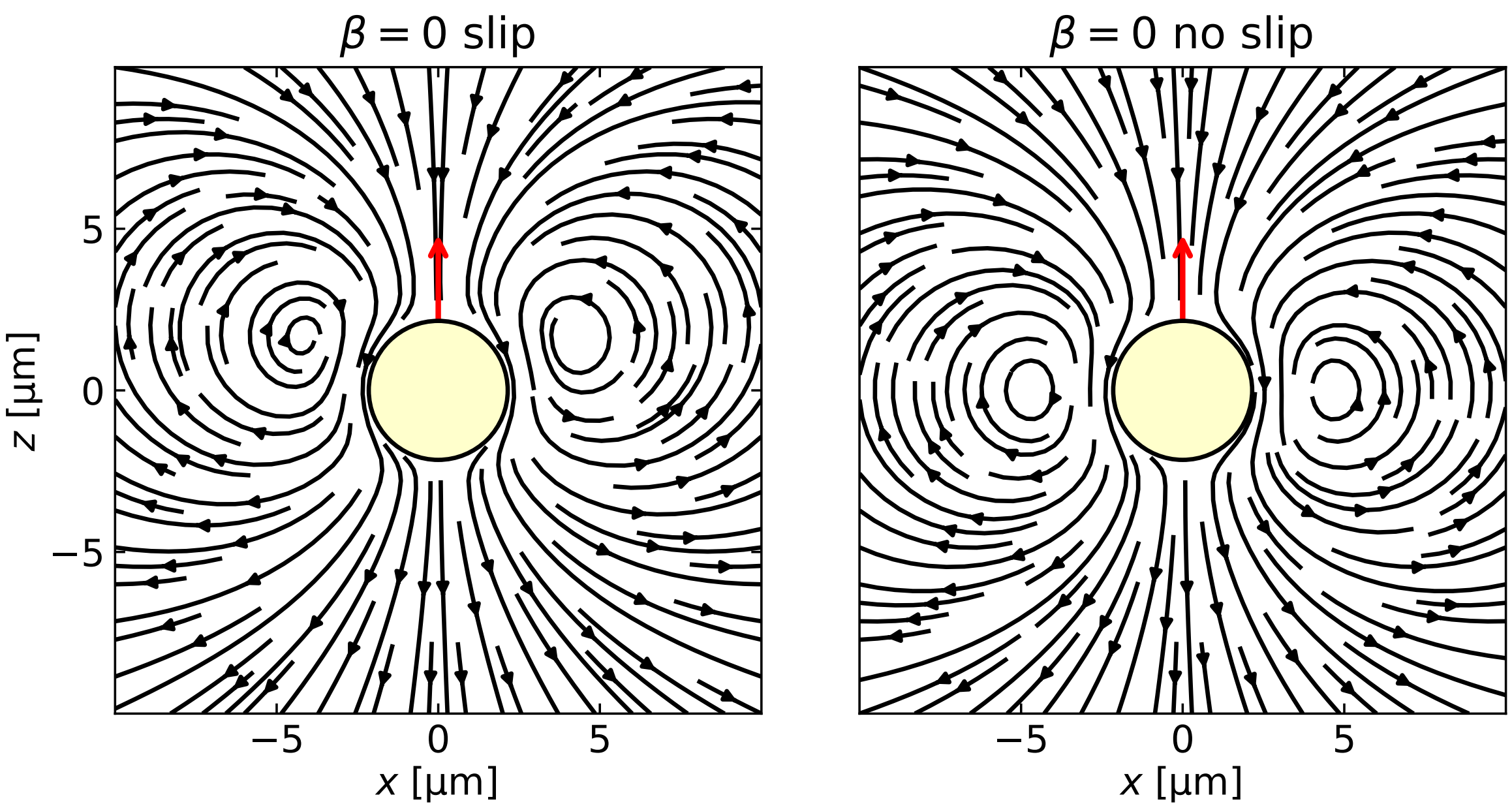

FIG. 2: Streamline plot for the neutral squirmer under confinement with thermoosmotic slip and no slip boundary condition at the substrate walls.

R1.4 The application of the Lighthill efficiency (Eq. 3) to the thermophoretic swimmer requires some care. The original idea behind this efficiency measure is that the dissipation is proportional to U^2, which makes the efficiency invariant of the velocity. For the swimmers reported here, velocity U is proportional to the power P (Fig. 4c). This means that the efficiency ϵ is no longer velocity-invariant. Minimizing the dissipation at a given speed or maximizing the speed at a given dissipation will not necessarily lead to the same outcome. As long as all experiments are carried out at the same swimming velocity there will certainly be no problem, but the limitations of the efficiency measure should be mentioned.

Response: We thank the reviewer for this important observation. The reviewer is correct that, in our system, the positivity constraint $q(\theta) \geq 0$ forces the monopole heating q_0 to scale linearly with the dipole coefficient q_1 (and hence with the swimming speed U). As a consequence, our efficiency

$$\epsilon = \frac{6\pi\eta a U^2}{4\pi a^2 q_0} \tag{1}$$

scales as $\epsilon \propto U$ rather than being speed-invariant as in Lighthill's original construction, where internal dissipation scales as U^2. Nevertheless, Lighthill's efficiency is not considering

any details of the physical mechanisms generating the flow field and is therefore not including the dissipation during flow generation.

All efficiency comparisons in the present work are performed at a fixed swimming speed (fixed $q_1 = -0.4\,\mu W\,\mu m^{-2}$), so that the β-dependence reported at constant U is well-defined, and the identification of $\beta^* \approx -1$ as the optimal swimming mode is unaffected by this issue. We have added a clarifying paragraph in the revised manuscript, explicitly noting that ϵ should be interpreted here as a benchmark figure of merit at the operating velocity, rather than as a velocity-invariant property of the swimming mechanism. Accordingly, the efficiencies reported in Fig. 4e should not be compared across systems operated at different swimming velocities.

Regarding the two optimization problems raised by the reviewer, minimizing dissipation at fixed U versus maximizing U at fixed dissipation, we note that in our experiment the natural operating constraint is the swimming speed, set by q_1. The optimization addressed in the main text therefore corresponds to minimizing P_{in} at fixed U, which is the experimentally relevant problem and yields a δ-like optimal illumination profile at the rear pole. In the revised Supplementary Information (S6) we have additionally solved the dual problem of maximizing U at fixed P_{in} explicitly, and obtain the same optimal profile. The two formulations therefore coincide for our system, even though they are not equivalent in general.

Reviewer 2

Summary of the paper

The authors demonstrate a thermophoretic microswimmer whose surface heating pattern can be dynamically programmed to tune its hydrodynamic flow field, and they use this control to study how physical constraints on propulsion affect energetic efficiency. They argue that the optimal swimming mode differs from the prediction of idealized hydrodynamic theory. In particular, the authors discuss that both biological and synthetic microswimmers move through viscous fluids by pushing and pulling the surrounding liquid, thereby creating characteristic flow patterns. Both types of swimmers are subject to physical constraints that restrict the kinds of flow patterns they can produce. These constraints are not taken into account in hydrodynamic theory, which predicts that neutral swimmers are the most efficient. As the authors note, experiments to date have not been able to systematically test how changing flow type affects efficiency, because existing systems have "prescribed" flow types that cannot be changed once created. To address this limitation, the authors extend an experimental system previously developed in their lab, consisting of spherical colloids decorated with gold nanoparticles, by adding dynamic control to the external mechanism that drives their propulsion. This allows them to switch between different flow types in real time. They develop a simple theory that predicts the heating pattern required to generate a specific flow field and use it to design the corresponding experimental intensity pattern. After showing that they can generate flow fields that closely match their theoretical predictions and measuring corresponding efficiencies, they find that pushers are more efficient than neutral swimmers. The authors compare this result with ideal hydrodynamic theory and argue that real-world constraints can alter what "optimal" behavior looks like.

General assessment

The authors build on previous work from the Cichos lab (e.g., Refs. 22, 36, 50), in which thermophoretic propulsion was generated by laser-induced heating within a feedback loop that tracked the particle and updated the heating position in real time. In that earlier system, controlling the position of the localized heat source determined the particle's direction of motion. In the present work, the authors introduce dynamic control over the illumination itself, enabling programmable and spatially structured heating patterns across the particle

surface and, consequently, real-time control over the generated flow field. They also develop a simple theoretical framework to determine the heating patterns required to produce a desired flow field, and they show good agreement between theoretical and experimental flow fields. The manuscript is well written and accessible even to non-specialists. The authors present a meaningful and useful extension of their experimental platform that should be of broad interest to the active matter community, particularly to researchers working on active colloids. A notable strength of the work is the ability to switch the hydrodynamic mode in real time for the same particle. This provides a cleaner and more convincing comparison than approaches based on different particle batches, where other properties such as shape, surface chemistry, and propulsion mechanism could vary simultaneously. The ability to switch and monitor modes in real time thus offers a compelling way to isolate the role of hydrodynamic mode itself. That said, while I appreciate the technical advance and find the work methodologically strong, I have concerns about the interpretation and framing of the results, which in my view are somewhat overstated. In particular, I am not fully convinced by the claims regarding the generality of the findings, the comparison with ideal theory and the definition of the key metric used to define efficiency, as outlined in the major points below. Thus, although in principle the results are interesting and, in my view, worthy of publication in Nature Communications, I would not recommend publication in the current form. Revisions are needed to clarify the scope and generality of the authors' claims, as well as their choice of efficiency metric and how it relates to the definitions used in the literature against which they compare their results.

Response: We thank the reviewer for carefully evaluating our manuscript. In response, we have substantially revised the introduction to make the scope of our claims explicit (isolated spherical thermophoretic swimmers in Newtonian fluids under the positivity constraint on surface heating) and to clarify the role of real-time control, and we have revised the discussion to address the efficiency-metric and constraint-induced-coupling issues raised in the major points.

Major points

R2.1 Optimality/notion of optimality:

The authors repeatedly make a broad claim about the generality of their result that the

"pusher mode is more optimal". For example, on page 3 they write: "We find a striking result: .... This demonstrates that physical constraints on control parameters fundamentally alter performance optima, shifting the optimal mode from neutral to pusher. Importantly, this principle extends beyond thermophoretic swimmers to any system where the propulsion mechanism imposes constraints on achievable surface flows." Based on the evidence presented, this statement appears overstated.

The manuscript's results concern a specific propulsion mechanism (thermophoresis) under the particular constraint of positive-only surface heating. However, "optimal swimming" is not a universal notion; it depends sensitively on imposed constraints, the environment, interactions, and even the chosen definition of efficiency (see below points). See also, e.g., the following references: [Zhu et al., Phys. Fluids (2012), where efficiency globally changes with the fluid, and neutral/puller swimmers remain competitive or better], [Klindt et al., PRE (2015), where biological swimmers do not minimize hydrodynamic dissipation, and efficiency is not the main design principle], [Bailey et al., PRE (2024)], and [Singh et al., Langmuir (2020), where performance is interaction-dependent]. For this reason, swimmer performance cannot be reduced to a single universal optimum, and there is no unique "best swimmer." This context dependence seems important and is not adequately discussed in the manuscript. The authors should discuss this context-dependency of "optimality" (beyond the discussion already included on page 12 comparing spherical and non-spherical swimmers), and either substantiate their broader claim more carefully or revise it to reflect the more limited scope of their finding.

Response: We thank the reviewer for this comment and agree that the notion of 'optimality' requires careful qualification. Our conclusions are restricted to isolated spherical swimmers in Newtonian fluids under the specific propulsion constraint imposed by thermophoretic self-propulsion with positive-only surface heating. Within this setting, the pusher mode emerges as the most efficient.

We fully agree that swimmer optimality is not universal but depends sensitively on propulsion mechanism, environmental rheology, swimmer interactions, confinement, and the chosen efficiency measure. The references suggested by the reviewer illustrate this clearly: Zhu et al. [47] show that fluid viscoelasticity reshapes the efficiency landscape, with neutral and puller swimmers remaining competitive depending on the fluid relaxation time; Klindt et al. [7] demonstrate that biological swimmers such as *Chlamydomonas* alternate between pusher

and puller phases during the stroke cycle, indicating that biological beating patterns are not optimized for hydrodynamic dissipation but reflect constraints of the underlying propulsion mechanism; Bailey et al. [46] highlight that optimality can be defined with respect to transport or collective objectives that need not coincide with single-particle efficiency; and Singh et al. [31] show that swimmer performance depends on hydrodynamic interactions and cannot be reduced to an isolated-swimmer figure of merit. We note in particular that the efficiency measure used by Zhu et al. [47] accounts only for bulk viscous dissipation and does not include the energetic cost of the propulsion mechanism, further underscoring the reviewer's point that conclusions about optimality depend on how the energetic cost is defined.

Accordingly, we do not claim the existence of a universally 'best' swimmer. The central conclusion of our work concerns the role of propulsion constraints in shaping efficiency: in the unconstrained classical squirmer model the neutral swimmer maximizes the Lighthill efficiency (or even the efficiency as calculated by Sabass and Seifert [44]), whereas once physically motivated constraints on the swimming mechanism are imposed and the true energy input is taken into account, the efficiency optimum can shift toward the pusher mode. The broader principle our work demonstrates is therefore not that pushers are universally optimal, but that propulsion-mechanism constraints can fundamentally reshape the efficiency landscape away from the predictions of unconstrained hydrodynamic theory.

We have revised the introduction and discussion to clarify overstated claims of generality and to make explicit that our results apply to isolated spherical thermophoretic swimmers in Newtonian fluids under the positivity constraint on surface heating. Specifically, the introduction now states that the spherical neutral squirmer is rarely realized in nature and serves as the canonical idealization for theories of efficient propulsion, and that the predicted optimum has been derived under the assumption that surface boundary conditions can be prescribed at will. In the discussion, we appended a context-dependency passage to the paragraph beginning "This finding suggests a broader principle: physical realizability constraints may universally shift optimal swimming modes...," acknowledging that swimmer optimality is shaped by the propulsion mechanism, environmental rheology, swimmer interactions, confinement, and the chosen efficiency measure, and citing the references suggested by the reviewer.

R2.2 In addition, the authors repeatedly emphasize that hydrodynamic theory predicts an optimum at $\beta = 0$ (neutral swimmer), whereas their results identify a negative value of β (pusher swimmer) as optimal. While a comparison with hydrodynamic theory may in principle be useful, there appears to be a fundamental difference in how efficiency is defined. In the present work (cf. Eq. (3)), efficiency is defined by comparing the mechanical output to the power absorbed through particle heating (i.e., the total energy input from the propulsion mechanism; that is, efficiency here depends on how energy is supplied). The conclusion that pushers are more efficient seems to reflect the specifics of the thermophoretic system with positive heating, rather than a universal statement about hydrodynamic optimality or a general hydrodynamic principle.

In particular, the efficiency in Eq. (3) contains the absorbed power (P_{abs}) in the denominator, rather than the viscous dissipation in the fluid used in the hydrodynamic theories cited by the authors. Thus, the efficiencies being compared appear to be fundamentally different. The authors should address this distinction explicitly and discuss how meaningful it is to compare the two optima directly, namely the hydrodynamic optimum at $\beta = 0$ and the negative-β optimum found here.

Indeed, a comparison with a treatment such as that in Ref. 44 seems more natural, since the definition of efficiency is more closely aligned with the one adopted by the authors. As the authors themselves note, Ref. 44 likewise finds that propulsion localized near the pole is the most efficient. In light of this agreement with a more directly comparable classical result, it is not clear how surprising the present finding actually is. Unless the authors can provide a compelling reason why comparison with the hydrodynamic models is more appropriate, the repeated characterization of their result as unexpected should be revised.

Response: We thank the reviewer for raising this issue. The reviewer is correct that the efficiency in Eq. (3) and the classical Lighthill efficiency are not directly comparable. The Lighthill efficiency uses bulk viscous dissipation in the fluid as the energetic cost. Sabass & Seifert [44] subsequently analyzed the full hydrodynamic dissipation for surface-driven swimmers and showed, via a boundary-layer decomposition, that for micron-scale phoretic swimmers the dissipation is dominated by the interfacial boundary layer where the quasi-slip is generated. Our efficiency goes one step further. The denominator $P_{\text{in}} = 4\pi a^2 q_0$ is the total absorbed power, which by energy conservation captures the sum of all dissipative costs of propulsion, namely bulk viscous dissipation, interfacial boundary-layer dissipation, and

thermal relaxation to the bath. A similar total-input perspective underlies the efficiency definition of Kreissl et al. [now Ref. 45] , who relate the input power to the chemical reaction rate sustaining the slip. We have made this distinction between the different efficiency definitions explicit in the revised manuscript, now present Kreissl et al. [now Ref. 45] as the appropriate classical comparator alongside the squirmer model, and revised the corresponding passages to avoid direct numerical comparison with the unconstrained $\beta = 0$ result.

We would, however, respectfully argue that our finding is not made unsurprising by the Kreissl result. Kreissl et al. [now Ref. 45] prove an upper bound on the efficiency functional that is saturated only by δ-localized surface activity at the poles, via a mathematical inequality on the Legendre projection of the surface activity. Their analysis does not address the physical origin of the optimum or the role of the positivity constraint in coupling the activity multipoles. In particular, they treat the positivity condition $f_J \geq 0$ as a model-specific feature, rather than as the physical mechanism that shifts the efficiency optimum. They also note that experimental realization of polar-reactive particles remains an open problem.

The central physical insight of our work is that the optimum is a consequence of constraint-induced coupling. Under the positivity requirement on the surface activity, all higher multipoles become coupled to the monopole, so that any propulsive mode (q_1) or mode-selecting mode (q_2) inevitably incurs a monopolar energetic cost (q_0). This coupling shifts the efficiency optimum away from the unconstrained $\beta = 0$ neutral mode toward the pusher regime. The same logic generalizes to any phoretic propulsion mechanism subject to a positivity constraint on the surface activity, such as catalytic reactions producing a single species or surface heating without cooling. We now state explicitly the corollary that the constraint, and hence the shift of the optimum, can be lifted by allowing sign-changing surface activity (heating and cooling, or sources and sinks for the same chemical species, as in self-electrophoresis). In such systems the unconstrained $\beta = 0$ optimum should be recovered. Our experiments provide a direct demonstration of a constraint-shifted optimum in a swimming system.

We have revised the manuscript to (i) make the distinction between the different efficiency definitions explicit and present Kreissl et al. [now Ref. 45] as the appropriate classical comparator, (ii) reframe the contribution as the identification and experimental verification of constraint-induced coupling as the mechanism shifting the efficiency optimum, and (iii) state explicitly the corollary that sign-changing surface activity would lift the constraint and recover the unconstrained optimum. Specifically, the corollary and the term *constraint-induced*

coupling are now stated in the discussion immediately after the sentence "This coupling is absent in idealized models where surface slip velocities can be prescribed arbitrarily."

R2.3 A further issue concerning the definition of efficiency itself, specifically the use of the power absorbed by the particle as the relevant input quantity: as the authors state, the absorbed power varies considerably between modes. Because the efficiency metric combines hydrodynamic drag with mode-dependent energy absorption, the conclusion that the pusher mode is optimal becomes somewhat ambiguous. Is the apparent optimality of pushers due to a more efficient flow field, or does it simply result from lower energy input requirements? In other words, pusher swimmers may appear more optimal not because of hydrodynamics, but because they absorb less energy.

Indeed, Figs. 4d and 4e seem to suggest that pusher swimmers are simply energetically cheaper, in the sense that they provide the most energy-efficient way to achieve a given propulsion speed, or equivalently, require the least energy input to reach the same speed. As a separate note, this definition appears problematic in the limiting case. Consider a hypothetical particle that absorbs no power at all. Then even a purely Brownian particle with an arbitrarily small but nonzero velocity would formally have infinite efficiency, despite not extracting any usable work from absorbed energy. The authors should therefore justify their choice of efficiency definition more carefully and explain why this apparent pathology does not undermine their analysis.

Response: We thank the reviewer for raising this point and agree that the framing requires clarification. In our framework, the observed optimum at $\beta \approx -1$ is not a statement that the pusher flow field is hydrodynamically optimal. Rather, it reflects the fact that the pusher minimizes the monopole heating contribution q_0, which is a part of the absorbed power that does *not* contribute to propulsion. At fixed swimming speed (fixed source dipole contribution $q_1 = -0.4\,\mu\mathrm{W}\,\mu\mathrm{m}^{-2}$), the input power

$$P_{\text{in}} = \iint_{\text{sphere}} q(\theta)\, dS = 4\pi a^2 q_0$$

attains its minimum at $\beta \approx -1$ (Fig. 4d). Physically, the pusher mode is the energetically least expensive way to generate a prescribed propulsion velocity, because it minimizes the energy dissipated into background heating that does not drive the slip. This is the meaning of “optimal” in our context: most effective extraction of directed motion from the absorbed

power, not optimal hydrodynamic flow structure.

This interpretation is independently supported by the temperature measurements in Fig. S5d. At the same laser power (4.35 mW), the pusher exhibits the smallest surface temperature increase, whereas neutral and puller-like swimmers reach substantially higher temperatures, confirming directly that the pusher minimizes the monopole heating contribution and reduces non-propulsive energy expenditure.

The physical origin of this minimization is the constraint-induced coupling discussed in our response to R2.2: under the positivity requirement $q(\theta) \geq 0$, the monopole q_0 is forced to track the higher multipoles, and the pusher configuration is the one that minimizes this coupling at fixed propulsive contribution. The framework therefore predicts a specific, mechanistic origin for the efficiency advantage of pushers, rather than appealing to a generic property of pusher flow fields.

Concerning the limiting case of a purely Brownian particle, we note that the apparent pathology arises from a category mismatch rather than from the efficiency definition itself. Our efficiency compares the Stokes-drag power $6\pi\eta a U^2$ associated with the *directed* (time-averaged) swimming velocity to the sustained power input that drives the surface activity. A purely Brownian particle has zero directed velocity ($\langle \mathbf{U} \rangle = 0$); its instantaneous thermal fluctuations are not work extracted from a driving source but rather equilibrium exchange with the bath, and on time averages relevant to swimming efficiency, both numerator and denominator vanish. The same indeterminate form arises for any efficiency definition applied to a passive system in equilibrium. The efficiency in Eq. (3) is defined and meaningful for actively driven swimmers that continuously consume energy to maintain self-generated surface gradients and sustained directed motion; in this regime, both the numerator and the denominator are well-defined and positive.

We have revised the manuscript to clarify the meaning of "optimal" in our context, to state explicitly that the pusher optimum reflects minimization of non-propulsive energetic cost rather than hydrodynamic flow-field optimality, and to make the domain of applicability of Eq. (3) (actively driven swimmers) explicit.

Additional points

A2.1 Unless I am mistaken, neutral swimmers can rarely be found in nature (microorganisms are not perfectly symmetric, have time dependent strokes, switch between modes). In addition, I am not aware of hydrodynamically neutral colloidal swimmers (artificial swimmers may achieve neutrality as a transient state using external fields). The authors only cite one theoretical article in the Introduction when they introduce the notion of "neutral" swimmer on page 2. Are the authors aware of any other examples of neutral swimmers (either biological or synthetic)? Maybe they would like to comment on that, especially in the context of comparing neutral-like flow in natural and laboratory settings to ideal theory.
Response: We thank the reviewer for this question and agree that neutral squirmers appear to be rather uncommon in nature. Most biological microswimmers exhibit geometric asymmetries, time-dependent stroke cycles, or dynamically changing flow fields, such that they cannot generally be classified as perfectly neutral squirmers in the idealized theoretical sense.

Nevertheless, there are several studies in which microorganisms have been approximated as nearly neutral squirmers. For example, Zayed et al. [9] modeled *Paramecium* as a neutral swimmer. Since *Paramecium* propels itself through the coordinated beating of many cilia that generate metachronal waves across the entire surface, its propulsion can effectively be represented by any slip velocity. In this context, Osterman et al. [10] found that a slip profile corresponding to $\beta = 0$ maximizes the hydrodynamic efficiency for ciliary propulsion. Another example is provided by Drescher et al. [11], who observed that *Volvox* exhibits approximately neutral squirmer characteristics in the context of hydrodynamic interactions near surfaces.

At the same time, the relatively small number of experimentally observed neutral squirmers suggests that biological systems are not necessarily optimized solely with respect to hydrodynamic propulsion efficiency. In living systems, the relevant optimization principles are likely far more complex and may involve robustness, maneuverability, sensing, nutrient uptake, synchronization, or metabolic constraints. The corresponding optimization functions are generally unknown.

In this context, we believe that our experimental platform is particularly valuable because it allows the controlled realization of neutral, puller-like, and pusher-like swimming modes within the same physical system. This makes it possible to experimentally investigate how propulsion constraints influence the resulting flow fields and efficiencies, and to directly

compare idealized theoretical optima with experimentally realizable swimming modes. We have revised the manuscript accordingly.

A2.2 The authors state in the Introduction that investigating the optimal swimming mode requires real-time control over the surface boundary conditions. The authors may want to explicitly clarify for their readers why real-time control is advantageous for their study.

Response: Due to the control of the illumination pattern at video rate (which we regard as real-time) and thus the surface quasi-slip velocity, we can switch swimming modes for the same particle on demand. This approach allows us to isolate the role of the hydrodynamic swimming mode itself, which makes it a more robust basis for comparison than other methods, where parameters such as particle type, shape, surface chemistry, or even the propulsion mechanism must be altered. The introduction was revised to clarify the role of a real-time control.

A2.3 The authors state in the concluding paragraph of the Introduction: "a perspective that opens new directions for exploring morphological intelligence and adaptive strategies in both biological and synthetic active systems." Can the authors clarify A) what morphological intelligence in synthetic matter is, and B) how this work may aid in exploring that, perhaps via an example?

Response: In synthetic active matter, morphological intelligence refers to the ability of a system to encode functionality ("learning") through interactions with its physical structure or geometry in an environment. In our context, morphological intelligence refers to the idea that the effective morphology of the swimmer is given by the surface flow it generates, rather than by its fixed geometric shape. This flow field determines how the particle interacts with its surroundings and thus encodes its functional behavior. In this context, morphological intelligence therefore describes how functionality can emerge from controlling this hydrodynamic morphology, without changing the particle's material structure.

In our work, we realize this concept by controlling the illumination pattern, which directly sets the surface slip velocity and thereby the resulting flow field. This enables programmable switching between different hydrodynamic morphologies (pusher, puller, neutral swimmers). Each of these modes leads to distinct transport and interaction properties.

For example, a particle could adapt its swimming mode depending on its task or envi-

ronment: adopting a mode that maximizes propulsion speed in homogeneous regions, and switching to a mode with stronger flow-mediated interactions or higher energy efficiencies when searching for or collecting targets. Residori et al. (arXiv:2405.12381) showed that swimmer navigation in heterogeneous environments strongly depends on both the swimming mode and the obstacle packing fraction. In particular, puller- and pusher-like swimmers can become trapped within obstacle arrays, while changes in the hydrodynamic flow field may allow them to escape again. In this sense, functionality is achieved not by changing the particle's material properties, but by reconfiguring its effective hydrodynamic signature providing a minimal platform to explore adaptive strategies in synthetic active systems.

We have also relocated the paragraph that originally introduced the morphological intelligence concept from the end of the Introduction to the closing paragraph of the Discussion, where it now functions as an outlook and is merged with the existing concluding statement on evolutionary and mechanism-based optimization. We feel this placement gives the concept a more natural setting, as it builds directly on the experimental and theoretical findings developed throughout the paper.

A2.4 Regarding Fig. 3:

A. The case $\beta = -\infty$ appears to deviate much more strongly from the theoretical curve than the other cases shown, for which the agreement is considerably better. Could the authors comment on the origin of this qualitative discrepancy between experiment and theory? For example, on the experimental curve there does not appear to be a clear maximum at negative x values, in contrast to the theoretical prediction.

Response: We thank the reviewer for pointing out this discrepancy. We agree that the case $\beta = -\infty$ exhibits a noticeably larger deviation from the simulated results compared to the other swimming modes, for which the agreement between experiment and simulation is considerably better.

The main reason is the experimental difficulty of accurately resolving the pure force-dipole flow field associated with the shakers. In the limit $\beta = -\infty$, the source dipole vanishes, such that propulsion is absent and the flow is dominated by the quadrupole contribution corresponding to a pusher shaker. Experimentally, this state is sensitive to small residual contributions from lower-order modes. In the experiment, even weak imperfections in the

illumination pattern or small residual source-dipole contributions can distort the ideal force-dipole structure and modify the resulting flow field.

The flow field of the shaker contains four vortices that must be resolved experimentally on length scales of approximately 1 µm. The upper vortex region at negative x-values is not sufficiently sampled in the measurements, which explains why the experimental curve does not exhibit a clearly resolved maximum in this region, in contrast to the simulated curves. By comparison, the positive x-direction is captured much more reliably, such that the experimentally observed flow field still clearly demonstrates the realization of a pusher shaker.

We have added a corresponding discussion to the manuscript to clarify the origin and limitations of the observed discrepancy.

B. I found the curves of the simulation results difficult to distinguish in Fig. 3; could the authors improve their visibility, e.g., by increasing the line thickness?

Response: We have increased the line thickness for better visibility.

C. In the Main text, it is stated that the "x-component remains symmetric about the x-axis with no variation in $|\beta|$ (Figs. 3c-e), while the z-component shows more complex behavior". I am not sure I understand this statement: the x-component seems to vary significantly across different values of β, with the maximum being at negative x for negative β but at positive x for zero and positive β, so there clearly seems to be variation with β in both x- and z- component. Relatedly, what is the nature of the transition in the flow field as β is varied continuously from minus one to zero?

Response: We thank the reviewer for asking this important question. Please see our answer to questions 2 of reviewer 1, R1.2, and the attached Figure 1.

A2.5 Since the functional versatility and adaptive behavior presented in this work is imposed/enabled through external, real-time control rather than intrinsic swimmer dynamics, I found different sentences as overstated. For example, on page 12: "This capability enables task optimization for specific environmental challenges ...", "The ability to toggle between these modes on demand facilitates adaptive navigation in complex environments.", "Beyond efficiency optimization, the demonstrated real-time reconfigurability enables functional ver-

satility: microswimmers can reversibly switch between acting as propellers, pumps, or stealth swimmers.". It would be helpful if the authors refine these statements to make clear that the reported adaptability does not represent fully autonomous/intrinsic system behavior.

Response: We thank the reviewer for this important point. We agree that the control loop in our current implementation, sensing, decision-making, and pattern generation, is realized externally through the microscope and spatial light modulator, and that this is a distinguishing technical feature compared with fully on-board, autonomous biological control.

We respectfully note, however, that adaptive behavior is most commonly defined functionally, as the modification of an agent's behavior in response to environmental conditions, and need not require that the underlying computation reside inside the agent. The broader concept of morphological/embodied intelligence we invoke in the discussion explicitly questions this internal/external boundary, since adaptive function in many biological and synthetic systems is distributed across the agent, its body, and its environment (e.g., stigmergic colonies, swarms, niche-constructing organisms).

We have nonetheless revised the contested passages to be precise about the locus of the control loop, distinguishing between the adaptive functionality the platform demonstrates (real-time switching between hydrodynamic modes in response to changing conditions) and the externally implemented control loop that realizes it. Fully integrated, on-particle sensing and feedback remain an important direction for future synthetic agents.

A2.6 Experimental details:

A. The authors use particles decorated with nanoparticles, with approximately 10% surface coverage. How consistent is this between particles in their batch? And can the authors comment on variations between particles and how this may affect their observed behavior?

Response: The microswimmers used in our experiments are commercially obtained particles, and we therefore expect the nanoparticle surface coverage to be reasonably homogeneous across the batch. This is consistent with previous experimental studies employing the same particles. Fränzl et al. [25] provide SEM images of representative particles (Fig.S1), from which the surface coverage of 10% was estimated.

Since it is experimentally not feasible to characterize every individual particle in the batch, we addressed possible particle-to-particle variations statistically by repeating all measurements with multiple particles (typically 5-6 or more for each condition). Any significant

variation in nanoparticle coverage would directly influence the local heating and therefore modify the surface temperature distribution and propulsion characteristics.

To assess the reproducibility quantitatively, the 5CB measurements were performed for at least 5 different particles for each squirmer parameter β. Variations in surface coverage would be visible as noticeable differences in the measured phase-transition radii. However, as shown by the relatively small experimental uncertainties in the Fig.2 of the manuscript and Fig. S5 and S6 in the SI, the measured surface temperature increments are highly reproducible. This indicates that particle-to-particle variations in the effective surface coverage are small and do not affect the observed swimmer behavior.

B. In the Optical Microscopy part of the Methods section: "Particle image velocimetry (PIV) is used to extract velocities and to calculate streamlines." The authors do not provide sufficient information on their analysis.

Response: Particle image velocimetry (PIV) was applied to time-resolved microscopy recordings of tracer particles dispersed in the fluid. The method relies on analyzing the displacement of tracer particles between consecutive image frames in order to reconstruct the underlying flow field. For this purpose, we used a custom-written Python-based PIV routine.

Each microscopy image was subdivided into square interrogation windows, and the local tracer displacement between two consecutive frames was determined using cross-correlation of the recorded intensities. The cross-correlation function was computed using a Fast Fourier Transform (FFT)-based convolution algorithm. The resulting correlation matrix exhibits a peak corresponding to the most probable particle displacement within each interrogation region, from which the local flow velocity was extracted.

To reduce fluctuations arising from Brownian diffusion of the tracer particles and experimental noise, displacement fields obtained from multiple consecutive image pairs were averaged before calculating the final velocity fields and streamlines.

We have included these methodological details, together with the relevant PIV analysis parameters, in the Supplementary Information S4.

C. The size of the gold nanoparticles is mentioned at the end in the Methods section. The authors may consider adding this information when they first introduce the system on page

3 and/or in the legend of Fig. 1.

Response: We thank the reviewer for this suggestion. We have added the information about the size of the gold nanoparticles at the beginning of the manuscript.

A2.7 Data reporting:

A. Reporting Summary is missing. The readers would want to see information on statistics: e.g., how many particles were analyzed etc.

B. An example of insufficient reporting: Legends of Fig. 2 and Fig. 3 and Fig. 4 in the Main text: ".. from multiple measurements". Can you clarify what multiple measurements means (e.g., from how many particles, or from how many measurements over the same particle)?

Response: We thank the reviewer for pointing this out and agree that the statistical information and reporting details should be stated more explicitly in the manuscript and Reporting Summary.

For the flow field measurements shown in Fig. 3, each squirmer parameter was measured using 5–11 different particles. For every individual measurement, tracer trajectories were recorded over approximately 4000 consecutive images. With an inverse frame time of 33 ms, this corresponds to a recording duration of about 132 s (≈ 2.2 min) per measurement.

For the measurements of moving particles, data were recorded for 3 different particles for each combination of squirmer parameter β and laser power. Each measurement consisted of approximately $5,000 - 10,000$ recorded images, corresponding to trajectory durations of approximately $3 - 6$ min per measurement.

For the temperature measurements, each squirmer parameter was characterized using 5 different particles.

We have included this information explicitly in the figure captions and the Methods section to improve the clarity and reproducibility of the manuscript.

C. Will experimental data or codes be deposited in a public repository?

Response: The experimental data and codes will be deposited in a public repository.

A2.8 In light of the many mentions of "constraint" and "optimal", a consideration of a more general constrained optimization problem would be interesting. While a full consideration

of the general problem might be beyond the scope of this mainly experimental work, the authors could consider adding a brief discussion of how one would approach this problem in a more general theoretical setting and perhaps consider the simplest case (positive power and optimizing efficiency) in the Supplement.

Response: We thank the reviewer for this suggestion. In the revised Supplementary Note S6.3 we now treat the constrained optimization problem more generally: starting from the positivity constraint $q(\theta) \geq 0$ on the surface activity, we show by variational analysis that the efficiency is maximized when the activity collapses onto a delta-like distribution at the rear pole, and we discuss how the same line of reasoning extends to other phoretic mechanisms with different constraints (for example net-zero or fixed-flux constraints). This places our finding within a broader constrained-optimization framework.

A2.9 Typos:

A. The sentence before Eq. S66 in the Supplement contains a couple of typos.

B. In the first sentence of Sec. S5.3, "relying to" should be "relying on".

Response: We thank the reviewer for pointing out these typos. We have corrected all of them.

Reviewer 3

In this manuscript, the authors develop an experimental approach that allows reversible on-demand switching between different swimming modes (pusher, puller, neutral mode) of thermophoretically-propelled particles. The authors subsequently use this technique to test theoretical predictions on swimming efficiency and find that their experiments contradict idealized hydrodynamic models. They allocate this finding to the requirement that the heat source density must be positive on the surface of the particles. While the work is interesting and has been executed well, its technical nature and most likely limited impact means that it will appeal mostly to a specialized audience. I therefore do not recommend publication in Nature Communications but in a more specialized journal.

Response: We thank the reviewer for the favorable assessment of the technical execution, but respectfully disagree with the scope and impact assessment.

Our work combines three contributions: (i) the first synthetic microswimmer whose hydrodynamic mode is reconfigurable in real time, giving experimental access to the full squirmer spectrum within a single particle; (ii) the identification of a general physical mechanism, constraint-induced coupling, by which positivity constraints on the surface activity couple all higher multipoles to the monopole and shift the efficiency optimum away from unconstrained hydrodynamic predictions; and (iii) the first experimental falsification of the classical neutral-squirmer optimum, a cornerstone prediction of active matter theory since Lighthill, showing that the optimum is set by mechanism-specific realizability constraints rather than by hydrodynamic dissipation alone. The mechanism applies to any phoretic propulsion system with a positivity-constrained surface activity and predicts a testable corollary, that sign-changing activity (heating and cooling, or sources and sinks for the same species) lifts the constraint and recovers the unconstrained optimum.

The platform and the principle are relevant well beyond thermophoretic swimmers, to communities working on collective dynamics, biological locomotion, and active matter more broadly. The platform makes immediately accessible a class of experiments that have until now remained purely theoretical, including the response of single swimmers to changes in their hydrodynamic mode under confinement, the collective behavior of mixed pusher-puller suspensions, and the role of swimming-mode reconfiguration in chemotactic and rheotactic navigation. We note that this assessment is in tension with Reviewer 1, who described

the work as "a major breakthrough that in principle allows novel experiments studying the interaction of different squirmers with walls or other swimmers," and Reviewer 2, who engaged at length with the conceptual implications of our efficiency finding. To make the scope explicit in the manuscript, we have revised the introduction to delimit the regime in which our claims apply (acknowledging that the canonical neutral-squirmer optimum has been derived under the assumption of arbitrary surface boundary conditions), reframed the closing paragraph of the discussion within the broader concept of hydrodynamic morphological intelligence, and added the corollary about sign-changing surface activity to the discussion (immediately after "This coupling is absent in idealized models where surface slip velocities can be prescribed arbitrarily"). The reviewer's specific points are addressed below.

Specific Points:

R3.1 The central conclusion of the manuscript relies on a constraint that is specific to thermophoretic propulsion, namely, that the heat source density must remain positive everywhere (q > 0). Does this constraint-driven optimization translate to non-thermally driven systems, such as chemically powered colloids? In chemical systems where reactions can act as both sources and sinks (removing the q>0 constraint), can alternative physical limitations similarly shift the optimal swimming mode away from the idealized hydrodynamic predictions?

Response: The specific constraint considered in our work, $q(\theta) > 0$, is particular to thermophoretic propulsion, where the local heat source density cannot become negative. However, the underlying constraint principle is expected to apply more generally to phoretic swimmers. Any physical constraint that restricts the surface activity or prevents arbitrary realization of slip velocities can modify the resulting efficiency landscape and thereby shift the optimal swimming mode away from the idealized predictions of unconstrained squirmer theory. In this sense, the central conclusion of our work is not tied specifically to thermophoretic propulsion, but rather to the role of propulsion-induced constraints which are generically present in phoretic swimming mechanisms.

For chemically powered colloids, local reaction rates may act both as sources and sinks, such that the positivity constraint $q(\theta) > 0$ no longer applies. Nevertheless, these systems are subject to different physicochemical constraints. For example, in Kreissl et al. [now

Ref. 45], charge conservation associated with solute production imposes a global constraint on the surface reactivity distribution. The authors showed that this constraint alters the optimal activity profile and leads to an optimal swimming state with reactivity localized near the poles.

We therefore expect that phoretic propulsion mechanisms generally introduce different constraints on the generated fluid flows and that these constraints fundamentally influence which swimming modes become optimal under experimentally realizable conditions.

R3.2 The authors note that they suspend swimmers in a thin liquid film. Looking at the methods, I noticed that this refers to a 5um film in which the 4.4um particles are suspended. What is the effect of this confinement on the fluid flow? (How) Do the results extend to thicker samples?

Response: We thank the reviewer for this question. The strong confinement provided by the thin liquid film (thickness $h \approx 5\,\mu\mathrm{m}$) is indeed relevant for the structure of the hydrodynamic flow field.

To address this, we performed numerical simulations of the flow field for varying confinement heights, as shown in Fig. 3. Using the neutral swimmer as a representative example, we find that the qualitative structure of the flow field depends on the degree of confinement. For open boundary conditions and weak confinements (large $h = 40\,\mu\mathrm{m}$), the flow resembles the bulk Stokes drag field around a particle. As confinement increases, the flow is progressively altered by the presence of the walls.

In the strongly confined regime corresponding to our experimental conditions ($h = 5\,\mu\mathrm{m}$), the flow field is significantly modified. We observe a source dipole-like flow field with two characteristic vortices. When slip boundary conditions at the confining walls are introduced in the simulations, the vertical z-position of the vortices is shifted, further illustrating the sensitivity of the flow field to boundary conditions.

Despite these changes in the flow field, the key result of our experimental study—the relative comparison between different squirmer modes under identical confinement—remains qualitatively unchanged regardless of the degree of confinement. All swimmer types are affected by confinement in a consistent manner, and the classification into neutral, pusher, and puller modes is preserved. Consequently, the conclusion that the pusher is the most efficient at transforming heat influx into output power also remains valid. Nevertheless,

we expect that measurements performed in thicker samples would yield better quantitative agreement with our analytical theory, which is derived for thick samples (bulk conditions). We have revised the manuscript and the Supplementary Information (S5) to include these discussions on confinement effects and the influence of slip boundary conditions on the measured flow fields.

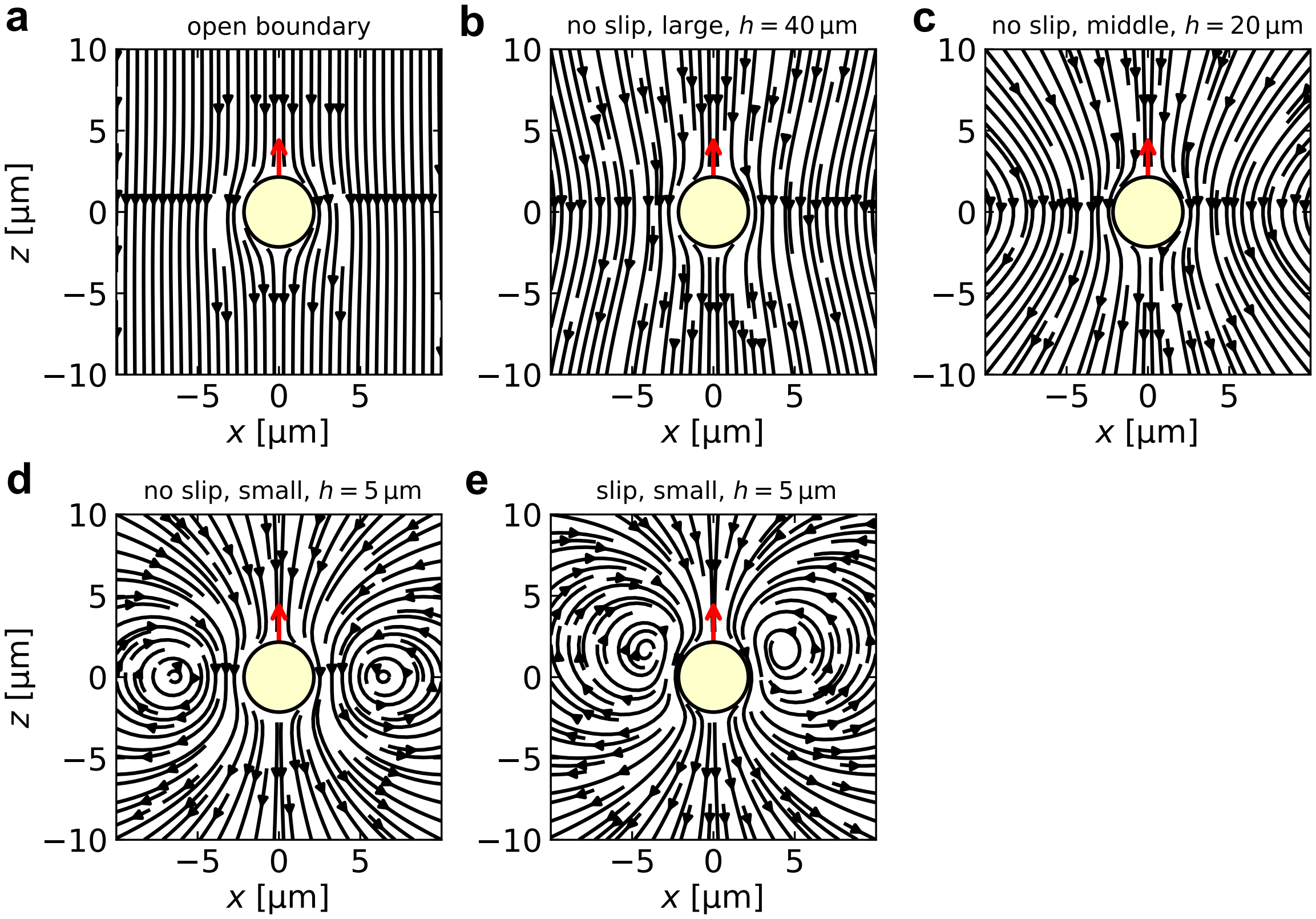

FIG. 3: **Simulation of flow fields. a** Flow field of a neutral squirmer under open boundary conditions. **b-d** With increasing confinement ($h = 40 - 5\,\mu$m), the structure of the flow field changes significantly due to hydrodynamic interactions with the confining walls. **d** In the strongly confined regime, the neutral swimmer exhibits a source dipole like flow field with two characteristic vortices. **e** Introducing slip boundary conditions at the walls shifts the vortices along the z-direction, resulting in a finite x-component of the velocity field at $z = 0$.

R3.3 The authors report overall swimming efficiencies on the order of 10^{-15} and mention that the majority of the absorbed thermal energy is utilized to maintain the localized temperature gradients, rather than to generate the propulsion. Given this massive baseline

energy cost, the authors should address whether the relative difference in absorbed power P_{abs} between the neutral and pusher modes is significant. A brief discussion whether this small difference is relevant to biological microswimmers or the design of synthetic swimmers would be helpful as well.

Response: We thank the reviewer for this important point. While the absolute swimming efficiencies are indeed very small ($\sim 10^{-15}$), the relative differences in absorbed power between the different swimming modes are nevertheless substantial and experimentally measurable. Supplementary Fig.S5 provides a quantitative decomposition of the absorbed power into monopole, dipole, and quadrupole contributions as a function of laser power. For example, for a laser power of 5 mW, the monopole contribution of the neutral squirmer is approximatly $q_0 = 2\,\mu W\,\mu m^{-2}$ whereas for the pusher mode it is reduced to $q_0 = 0.8\,\mu W\,\mu m^{-2}$. This corresponds to a reduction of roughly 60% in the monopole heating contribution. Since the monopole primarily reflects homogeneous background heating that does not contribute to propulsion, this reduction represents a significant decrease in energetically wasted power. The observed differences in swimming efficiency therefore arise from clearly distinguishable differences in the required energetic input for maintaining the propulsion mechanism.

This finding is further supported by the 5CB temperature measurements shown in Fig S5d and S6. For the same laser power ($P = 4.35$ mW) the pusher exhibits the lowest surface temperature increase of approximately 7 K, whereas neutral and puller-like swimmers reach substantially higher temperatures of about $11 - 13$ K. Thus, the latter modes require approximately $60 - 85\%$ larger temperature increases to generate the same propulsion velocity. These measurements directly demonstrate that the relative differences in absorbed power are experimentally significant.

At the same time, we agree that the absolute efficiency values should not be interpreted as biologically optimized efficiencies. In our system, the dominant energetic cost originates from maintaining localized temperature fields. We therefore view our results primarily as a proof of principle demonstrating how propulsion constraints and energy allocation shape swimming modes and their relative efficiencies. The ability to control and minimize non-propulsive dissipation pathways may become relevant for the design of synthetic microswimmers operating under limited energy budgets or in collective active-matter settings.

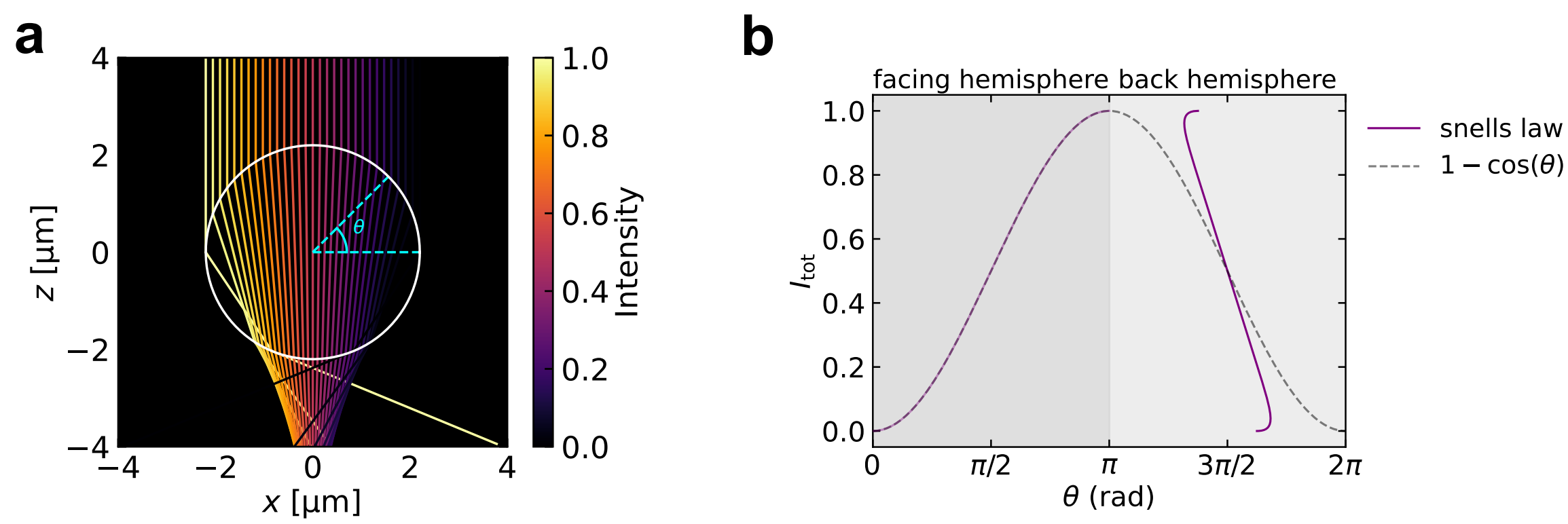

FIG. 4: **Refraction of illumination pattern inside the microswimmer. a** Ray-tracing simulation of the incident laser illumination for the neutral squirmer mode. The color map represents the imposed linear intensity profile, while the lines indicate representative light rays propagating through the particle and refracted according to Snell's law at the particle interface. The refractive indices used in the simulation are $n = 1.6$ for the melamine formaldehyde particle and $n = 1.33$ for water. **b** Surface intensity profile $I(\theta)$ obtained from the ray tracing along the particle surface compared to the theoretical neutral squirmer profile proportional to $1 - \cos\theta$.

R3.4 Can the authors elaborate why the assumption that the same illumination pattern is present on both sides of the particles holds? I would think that the incident laser illuminates the particle only on one hemisphere.

Response: In the experiments, the laser illuminates the particle from one side, while in the simulations a full three-dimensional temperature field is applied around the particle. In principle, optical refraction inside the particle can modify the illumination pattern between the front and back hemispheres. To assess this effect, we simulated representative light rays propagating through the particle using Snell's law, as shown in Fig. 4. For the ray tracing, we considered the illumination profile of the neutral squirmer, corresponding to a linear intensity profile, which is illustrated by the color code in Fig. 4a. The refractive indices were chosen as $n = 1.6$ fro the melamine formaldehyde particle and $n = 1.33$ for the surrounding water. The ray tracing demonstrates that the illumination pattern on the back hemisphere is indeed slightly modified due to refraction (see Fig. 4b), but the overall spatial structure of the imposed illumination profile remains well preserved across the particle surface. Importantly, the experimentally measured hydrodynamic flow fields are reproduced well

by the numerical simulations assuming the idealized three-dimensional temperature profile. This indicates that possible asymmetries induced by refraction are sufficiently small and do not significantly affect the resulting hydrodynamic flow fields within the experimental accuracy of our study.

R3.5 It might be helpful for the non-expert reader to include a brief explanation how the spatial coefficients of the incident light governs the propulsion force.

Response: In our system, propulsion is generated by thermo-osmotic boundary flows along the particle surface. These flows are induced by temperature gradients, which arise from a spatially varying surface heat source density $q(\theta)$. The local heat generation is determined by the absorbed laser intensity of the gold nanoparticles decorating the particle surface. Consequently, by controlling the spatial laser intensity profile, we directly control the surface temperature field and thereby the induced slip flow responsible for propulsion.

Due to the axial symmetry of the system, the heat source density can be expanded in Legendre polynomials. Restricting the expansion to second order yields

$$q(\theta) = q_0 + q_1 \cos\theta + q_2 \frac{1}{2}(3\cos^2\theta - 1)\,.$$

Since the absorbed laser intensity is proportional to the local heat source density, the coefficients q_0, q_1 and q_2 directly determine the geometry of the applied illumination pattern.

Using the relation $\cos\theta = z/a$, where z denotes the coordinate along the propulsion axis and a the particle radius, the heat profile can equivalently be written as

$$q(z) = q_0 + q_1 \frac{z}{a} + q_2 (3\frac{z^2}{a^2} - 1)\,.$$

As an illustrative example, for a neutral swimmer one has $q_2 = 0$, such that the illumination profile becomes linear along the propulsion axis,

$$q(z) = q_0 + q_1 \frac{z}{a}\,.$$

The coefficients q_0, q_1 and q_2 are directly related to the squirmer modes B_1 and B_2, as derived in detail in the Supplementary Information. In particular, $q_1 \propto B_1$, meaning that the dipole heating mode determines the propulsion velocity, while $q_2 \propto B_2$, corresponding to the force-dipole contribution that governs the far-field hydrodynamic signature. By tuning the ratio $q_2/|q_1|$, i.e. by changing the geometry of the illumination pattern, we can therefore realize different swimmer types characterized by the squirmer parameter

$$\beta = \frac{B_2}{|B_1|} \propto \frac{q_2}{|q_1|}\,.$$

R3.6 Does the laser which generates the heat affect the nanoparticles used as tracers for the flow?

Response: The illumination pattern generated by the spatial light modulator is spatially confined and designed to match the size of the microswimmer, as illustrated in Figs. 1 and 2 of the manuscript. As a result, the gold nanoparticles used as tracer particles are located outside the illuminated region and therefore are not exposed to the heating laser.

Consequently, the tracer particles are not affected by optical heating or radiation forces induced by the laser itself. Instead, they primarily follow the fluid flow generated by the swimmer, which allows them to serve as reliable probes for the PIV measurements.

R3.7 Some more details on how the PIV was done (software package) would be nice to add.

Response: Please see the answer to A2.6.B . We will include the methodological details, together with the relevant PIV analysis parameters, in the Supplementary Information S4.

Reviewer 4

I co-reviewed this manuscript with one of the reviewers who provided the listed reports. This is part of the Nature Communications initiative to facilitate training in peer review and to provide appropriate recognition for Early Career Researchers who co-review manuscripts.

Response: We thank the reviewer for reading and co-reviewing the manuscript.

Reviewer 5

I co-reviewed this manuscript with one of the reviewers who provided the listed reports. This is part of the Nature Communications initiative to facilitate training in peer review and to provide appropriate recognition for Early Career Researchers who co-review manuscripts.

Response: We thank the reviewer for reading and co-reviewing the manuscript.

DETAILED RESPONSES TO REVIEWER COMMENTS

Reviewer 1

The comments from my first report are all addressed in a satisfactory manner, I recommend the paper for publication in its current form.

Response: We thank the reviewer for the careful evaluation of our revised manuscript and for the positive assessment. We are pleased that the revisions have satisfactorily addressed the concerns raised in the first review and appreciate the recommendation for publication.

Reviewer 2

I co-reviewed this manuscript with one of the reviewers who provided the listed reports. This is part of the Nature Communications initiative to facilitate training in peer review and to provide appropriate recognition for Early Career Researchers who co-review manuscripts.

Response: Thank you for co-reviewing our manuscript.

Reviewer 3

I thank the authors for answering my questions and clarifying the points I raised in their reply. I particularly appreciate the new introduction in response to the question from another referee, which makes a clearer case for their work. I agree that in-situ adjustment of the hydrodynamics is novel and potentially exciting, but there are open questions as to whether this potential can be used, for example, at what distances can two particles be individually controlled. Despite many improvements, I still find the manuscript better suited for a more specialized journal.

In response to my earlier comments:

- R.3.1. I appreciate their reply and believe that it would be useful to integrate this clarification in the manuscript.

Response: Thank you for this suggestion. We added the main points in the discussion of the manuscript.

- R.3.3: As shown in the new simulations, the flow profile changes significantly in confinement, and I find this is not very clearly described in the text.

Response: We thank the Reviewer for this comment. We agree that the substantial effect of confinement on the measured flow profiles deserves a more prominent and explicit presentation than the original text gave it. The effect is discussed on p. 8, where we note that immobilization and the substrate introduce an additional Stokeslet contribution and that the thin-film geometry and wall thermo-osmotic flows reshape the field relative to the unbounded squirmer. Following the Reviewer's suggestion, we have expanded this passage to (i) state the three confinement mechanisms separately, (ii) describe their observable consequences for the velocity profiles, and (iii) point the reader directly to the comparison between the confined simulation (Fig. 3b) and the idealized unbounded case (Supplementary Fig. S7), where the magnitude of the change is most apparent.

- R.3.4: Their reply on why they believe that the assumption that the illumination pattern is the same on the front and backside of the particle has as far as I can see not been integrated in the manuscript. I would recommend the authors to do this.

Response: Thank you very much again for this recommendation. We have meanwhile put additional effort into exploring this issue quantitatively. This is documented in the new Supplementary Note S2.4, which is now also cited in the main text. In Supplementary Note S2.4, we compare the intensity patterns that a single Gaussian beam, focused to the sample plane containing the particle center, generates on the front and on the back side of the particle. The calculation has been carried out with a generalized Lorenz–Mie theory (GLMT) code for off-axis Gaussian beam scattering developed in our group. The results confirm the ray-optics picture in first approximation: the angular position of the transmitted intensity maximum on the back side scales linearly with the lateral displacement of the beam. The illumination pattern on the back side is thus not identical to that on the front side — the transmitted light forms an upright, demagnified image of the front-side pattern around the rear pole — but the linear and sign-preserving mapping retains the symmetry and the ordering of the imposed intensity profile. As detailed in Supplementary Note S2.4, the low-order heating moments, which determine the temperature profile and thus the swimming mode, are therefore unaffected, and residual sharp features are smoothed out by heat conduction.

This is also consistent with our liquid-crystal thermometry (Supplementary Note S3), where the measured isotherms agree with the analytical temperature profiles.

- I would also like to comment on their new outlook. I appreciate their openness that the swimmer's adaptability stems from external control. However, the statement "adaptability, sensing and decision making" can be confused with advanced sensing and adapting of the swimmer to the environment, when I think only the position of the particle is detected and the laser position adapted to it. Moreover, I don't see what the relation is with reference 48 which concerns a macroscopic robot that in no way resembles their work. I also don't understand how they will integrate such these abilities on the robot level. In short, I would tone down this paragraph.

Response: We thank the Reviewer for this comment. We agree that, at the level of the particle, the present system performs only position detection followed by operator-selected mode switching, and we have revised the paragraph so that the claims made for the results reported here are restricted accordingly. We have removed the reference to the macroscopic robot [previously 48], which we agree does not bear on the microscopic system studied here directly. We have also rephrased the outlook to make clear that the control loop is closed externally through the microscope and spatial light modulator rather than on board the particle, and we now point to bringing the hydrodynamic mode under the same closed-loop, reinforcement-learning control recently demonstrated for this thermoplasmonic swimmer platform (Paul et al.) as a direction for future work, without implying that environmental sensing or autonomous decision-making is demonstrated by the present results.

Reviewer 4

The revised version of the paper is substantially improved. I find it easier to follow, thanks to the definition of efficiency and range of validity of the results being clearer. Apart from the two minor comments below, I believe that this work is now of sufficient interest and quality to be published in Nature Communications.

Response: We thank the reviewer for the positive assessment of our revised manuscript and are pleased that the concerns raised in the first round of review have been satisfactorily

addressed. We appreciate the recommendation for publication.

Minor comments

1. In Fig. 3, I would suggest using a different color for the experimental results for $\beta = \pm\infty$ and the theoretical curves for $\beta = \pm 6$. Alternatively, the authors could include the theoretical curves for $\beta = \pm\infty$.

Response: Thank you for this suggestion. We changed the color for $\beta = \pm\infty$ to black and also included the theoretical curves for $\beta = \pm\infty$.

2. While I appreciate the added details on the statistical analysis, the information could be made more concrete, e.g., in the Methods section. For example, in the caption of Fig. 4, it is unclear what exactly is meant by "at least 3 different particles". Are the error bars in Fig. 4d larger for positive β than negative β because fewer particles were measured for these parameter values?

Response: We thank the reviewer for this suggestion. We have clarified the number of particles used for each dataset in the Methods section. Specifically, for the swimming speed measurements, data were recorded from five different particles for the neutral squirmer at each laser power and from three different particles for both the pusher and puller modes at each laser power. Each measurement consisted of approximately $5,000 - 10,000$ recorded images, corresponding to trajectory durations of $3 - 6$ min.

The temperature measurements underlying Fig. 4d were performed on five different particles for each squirmer parameter. The error bars therefore reflect the variability of the measured temperature profiles, rather than differences in the number of particles analyzed. We have clarified the corresponding statistics in the Methods section.

Reviewer 5

I co-reviewed this manuscript with one of the reviewers who provided the listed reports. This is part of the Nature Communications initiative to facilitate training in peer review and to provide appropriate recognition for Early Career Researchers who co-review manuscripts.

Response: Thank you for co-reviewing our manuscript.

DETAILED RESPONSES TO REVIEWER COMMENTS

Reviewer 3

The authors have addressed my requests satisfactorily.

Response: We thank the reviewer for their positive assessment and are pleased that our revisions have addressed all of their concerns.

Reviewer 4

The authors have satisfactorily addressed all points, and I recommend the manuscript for publication.

Response: We sincerely thank the reviewer for their positive evaluation of our revised manuscript and for recommending it for publication.

Reviewer 5

I co-reviewed this manuscript with one of the reviewers who provided the listed reports. This is part of the Nature Communications initiative to facilitate training in peer review and to provide appropriate recognition for Early Career Researchers who co-review manuscripts.

Response: Thank you very much for co-reviewing our manuscript.
